# Building a DNA barcode library for the freshwater fishes of Bangladesh

**DOI:** 10.1038/s41598-019-45379-6

**Published:** 2019-06-28

**Authors:** Md. Mizanur Rahman, Michael Norén, Abdur Rob Mollah, Sven O. Kullander

**Affiliations:** 1 University of Dhaka, Department of Zoology, Dhaka, Dhaka, 1000 Bangladesh; 2 Swedish Museum of Natural History, Department of Zoology, SE-104 05 Stockholm, Sweden

**Keywords:** Biodiversity, Zoology

## Abstract

We sequenced the standard DNA barcode gene fragment in 694 newly collected specimens, representing 243 species level Operational Barcode Units (OBUs) of freshwater fishes from Bangladesh. We produced *coi* sequences for 149 out of the 237 species already recorded from Bangladesh. Another 83 species sequenced were not previously recorded for the country, and include about 30 undescribed or potentially undescribed species. Several of the taxa that we could not sample represent erroneous records for the country, or sporadic occurrences. Species identifications were classified at confidence levels 1(best) to 3 (worst). We propose the new term Operational Barcode Unit (OBU) to simplify references to would-be DNA barcode sequences and sequence clusters. We found one case where there were two mitochondrial lineages present in the same species, several cases of cryptic species, one case of introgression, one species yielding a pseudogene to standard barcoding primers, and several cases of taxonomic uncertainty and need for taxonomic revision. Large scale national level DNA barcode prospecting in high diversity regions may suffer from lack of taxonomic expertise that cripples the result. Consequently, DNA barcoding should be performed in the context of taxonomic revision, and have a defined, competent end-user.

## Introduction

Fish and fisheries play an important role in Bangladesh’s economy, nutrition and culture. With 47 609 km^2^ of inland water bodies, it is the third inland fish producing country in the world after China and India^1^. The annual fish production from inland waters is estimated to be 3,496,958 mt, of which 1,163,606 mt comes from inland open waters^1^. Fish is second staple food in Bangladesh and alone supplements about 60% of animal protein in the daily dietary requirement^2^. Open water fishery is still the primary source of food fishes for the larger population. The Hilsa *(Tenualosa ilisha*) capture fishery alone contributes about 12% of the fish production^1^ and provides outcome for 2.5 million people^2^.

Bangladesh sits in between the biologically rich and diverse Indo-Burma and Eastern Himalaya regions, and is traversed by three of Asia’s largest rivers, the Ganga, Brahmaputra, and Meghna, which reach the Bay of Bengal in Bangladesh. Nonetheless, Bangladesh has one of the most incompletely known national freshwater fish faunas in Asia. As with other tropical countries, diversity estimates for Bangladeshi freshwater fishes are uncertain. Estimates of freshwater fish species vary from 237^3^ to 247^4^, 260^5^, or 267^6^, but those numbers include migrating and estuarine species; an estimate of riverine species only gives 104 species^5^. From several books and review papers on Bangladeshi fishes^4,6,7^, it is evident, however, that the taxonomy used is outdated and not harmonized with the taxonomy employed globally or even in neighbour countries. Apparently, considering the economic importance of inland fishery and the expected richness of the fish fauna, and in the absence of an expert based taxonomy, DNA barcoding may be an important component in biological conservation and management of biodiversity and fishery of Bangladeshi freshwater fishes.

DNA barcoding is a tool based on the observation and premise that each species is genetically distinct — has a unique DNA. Unique sequences of DNA from expert-identified specimens enable construction of a library of species-specific DNA sequences, “DNA barcodes”, against which unidentified samples can be matched^8,9^. DNA barcodes are useful for identification of both fresh specimens and market products such as frozen fish^10,11^. The standard barcode sequence, a fragment of the mitochondrial *cytochrome c subunit I* gene (*coi*), is also frequently used in phylogeographic and phylogenetic analyses^12,13^, but concerns have also been raised that the utility of barcoding has been overstated and that dependency on single markers may lead to deficient taxonomy^14,15^.

Here, we present the results and lessons from sequencing freshwater fishes collected in markets and natural habitats in Bangladesh, 2014–2016, in a study strictly aimed at a complete *coi-*based DNA barcode reference library of Bangladeshi freshwater fish species, introduce a new term for species level DNA sequence clusters interpreted as candidates for DNA barcodes: the Operational Barcode Unit (OBU).

## Results

**Figure 1:**
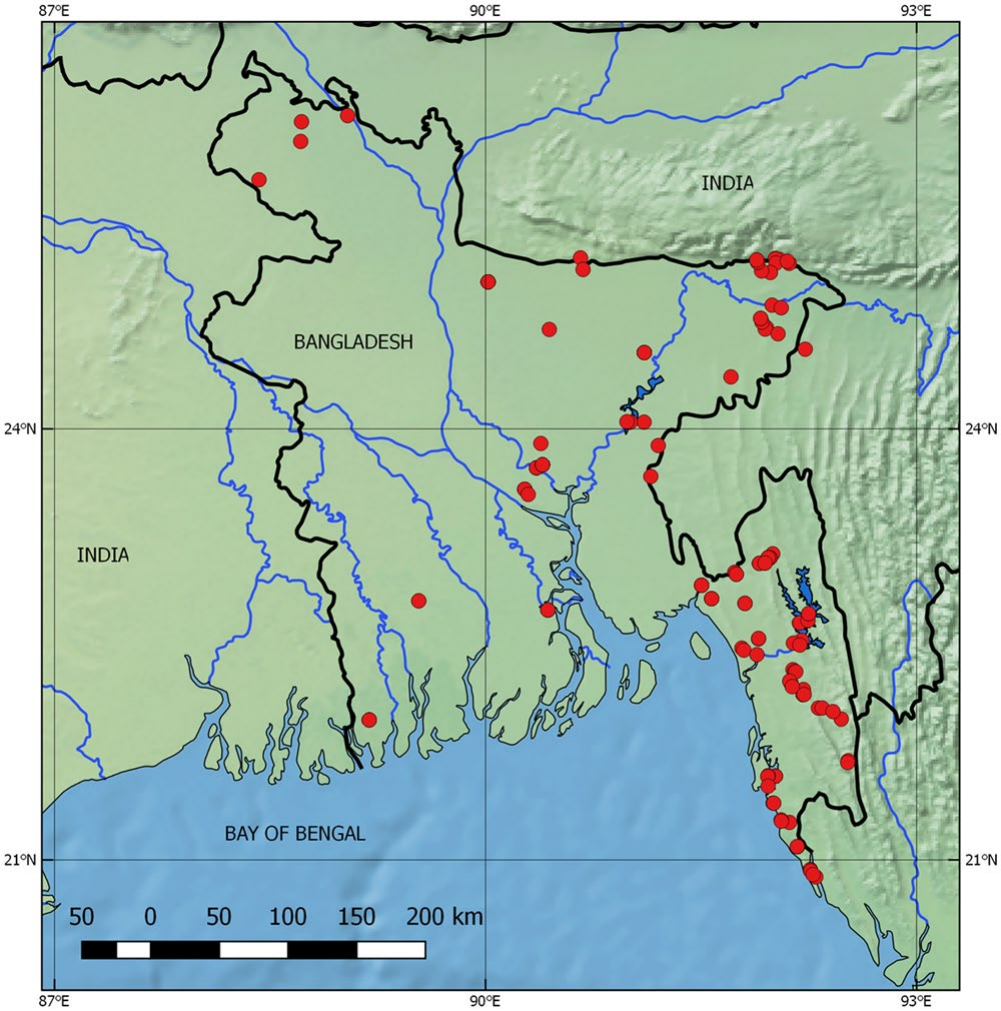
Map of collecting sites in Bangladesh, 2014–2016. A symbol may cover more than one collecting site. The map was constructed in QGIS 2.18.1 (QGIS Development Team, 2016. QGIS Geographic Information System. Open Source Geospatial Foundation. http://qgis.org), using free vector and raster map data made available by Natural Earth (http://naturalearthdata.com). All maps are in the public domain (http://www.naturalearthdata.com/about/terms-of-use/).

**Figure 2:**
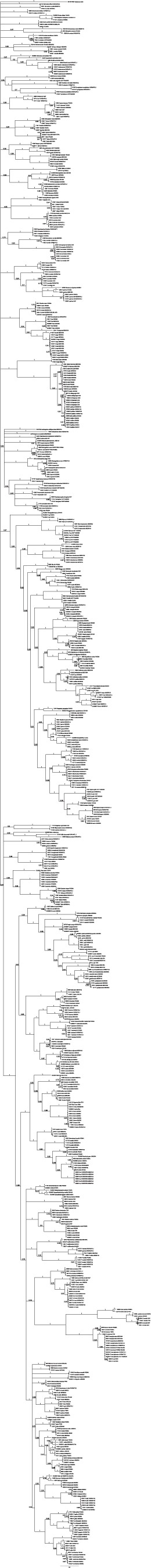
Phylogram of relationships of Bangladeshi fish species, based on Bayesian analysis of the standard DNA barcode fragment of the mitochondrial *coi* gene. The scale bar indicates expected number of substitutions per site. Terminals are identified by NRM or DU tissue sample collection identifiers (metadata, including GenBank Accession Numbers, in Supplementary Table S2).

**Figure 3:**
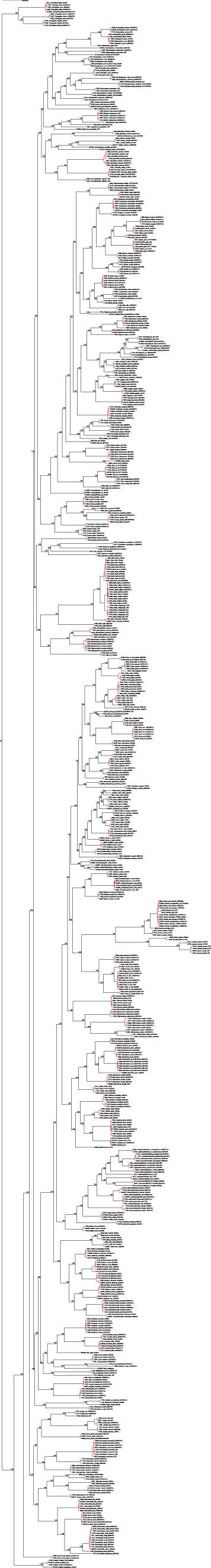
mPTP phylogram of Bangladeshi freshwater fish species based on the standard barcode fragment of the mitochondrial *coi* gene. The scale bar indicates expected number of substitutions per site. The clusters marked with red were resolved as species by the mPTP analysis. Terminals are identified by NRM or DU tissue sample collection identifiers (metadata, including GenBank Accession Numbers, in Supplementary Table S2).

Fish specimens were obtained from 150 collecting events and additional ad hoc sampling, including both markets and natural habitats in Bangladesh (Fig. 1). Phylogenetic and mPTP trees based on the Bayesian tree are shown in Figs 2 and 3. OBUs/species distinguished by the mPTP analysis are listed in Table S1. Metadata for sequenced specimens are summarized in Table S2.

The mPTP analysis found 243 OBUs (Table S1), representing 53 families. Most of these OBUs relate to single species verified by morphological examination. Using a scale 3 (lowest) to 1 (highest), the species determination expertise levels for the vouchers were estimated to be 3 in 82 OBUs, 2 in 17, and 1 in 46; 99 determinations were classed as unqualified.

Correcting for disagreement with the trees (Figs 2 and 3), and consideration of morphological analyses, suggests recognition of 237 candidate species. The majority of the OBUs are uncontroversial, representing well-known, common species. There was no indication of contamination. Five species descriptions were already published based on the present material^16–20^.

Two species failed sequencing (*Taenioides cirratus*, *Johnius coitor*). One probable nuclear pseudogene (NUMT) was found in *Pseudapocryptes elongatus* (OBU 14). One species had two mitochondrial lineages without morphological or nuclear DNA divergence (*Brachygobius nunus* OBU 10–11), and one species had two distinct populations separated by highly divergent mitochondrial and nuclear sequences but without detected morphological divergence (*Danio rerio* OBUs 160–161). Introgression was found in *Devario anomalus* (OBUs 152–153). Other OBUs may lack morphological support, as detailed in the discussion.

Several cases of putative cryptic species were recorded, as detailed below. Here we consider as putative cryptic species OBUs that are genetically distinct, but not reciprocally diagnosable morphologically. Several of the putative cryptic OBU pairs consist of one OBU in the Meghna River drainage and another in the Karnafuli and Sangu River drainages.

## Discussion

OBUs in the Bayesian tree (Fig. 2) and the mPTP analysis (Fig. 3, Table S1) were monophyletic at higher levels, except that the trees came out unresolved or with OBUs in unexpected position, as an artefact of the wide sampling (spanning the entire Actinopterygii) with several long branches without close relationship to other taxa in the tree. This affects, e.g., *Psilorhynchus (*Psilorhynchidae), nested within the Cyprinidae, and *Butis humeralis* (Eleotridae), within the Gobiidae). The objective of the tree-based analysis is, however, not phylogenetic relationships but species delimitation by branch clusters. The mPTP analysis reported 243 “species” but there are discordances with the tree. The “species” in the mPTP output includes potential or probable cases of excess taxonomic splitting and lumping, but also a number of ghost OBUs. In the following, OBU numbers are based on the mPTP species numbers from the analysis based on the Bayesian tree.

To investigate the influence of the choice of method for creating the phylogenetic tree used as indata for the mPTP species delimitation analysis, we used two different Maximum Likelihood (ML) software, RAxML and PHYML, to create Single Most Likely Trees, and analysed those as for the Bayesian tree. All three methods of analysis resulted in the same OBUs, with the exception of *Rasbora rasbora* (OBU 149) and *Microphis cuncalus* (OBU 25), which were recovered as single OBUs from the BI tree, but as two OBUs each from the ML trees. The genetic variation within the two putative species was intermediate between typical intra- and inter-specific variation, the two *M. cuncalus* (from Padma and Karnafuli) and three *R. rasbora* (from Padma and Meghna) sequences having an uncorrected furthest pairwise-p distance of 1.7% and 1.2%, respectively. Morphological examination did not find support for recognizing more than single species within OBUs 25 and 149.

A GenBank BLAST search of OBUs 4–6 identified them all as *Glossogobius giuris*, but they represent three genetically and morphologically distinct species. The common *G. giuris* morphology was represented by OBU 5, whereas OBU 4 is more similar to *G. aureus* from Hong Kong. OBU 6 remains unidentified. Our identification of OBU 4 is highly tentative.

OBUs 10 and 11 represent a single species, *Brachygobius nunus*. Morphologically identified specimens of *Brachygobius nunus* from the Turag River had distinctly different *coi* sequences; i.e., there were two mitochondrial lineages within a single population of *B. nunus*.

Species of *Pseudolaguvia*, OBUs 57–62, showed up as six distinct lineages in the mPTP tree (Fig. 3), but got split into seven species (Table S1), which is probably unwarranted. Unfortunately, it was not straightforward to identify some of the included lineages with named species.

Three specimens of *Pseudapocryptes elongatus* (OBU 14) were sequenced, and all three sequences had a unique 1 basepair (thymine, T) insertion at position 622 in the complete *coi* sequence (corresponding to position 6142 in the reference genome of *Parapocryptes serperaster*, GenBank accession number NC_029455). This insertion creates a TAG stop-codon and frame shift, which would render the *coi* protein nonfunctional, strongly suggesting that the sequences are not of mitochondrial *coi*, but of a nuclear pseudogene (NUMT). Resequencing the specimens with different primers produced the same result. There are 12 *coi* sequences of *P. elongatus* deposited at GenBank, but only two, AF391394 and KT124739, are long enough to include the insertion. AF391394 is identical to our sequences, with the exception that there is no T insertion at position 622. KT124739 contains nine differences to our sequences, out of which four are unique insertions, but also the T insertion at position 622. It is possible that our sequences are a chimera of the *coi* gene and a nuclear pseudogene, although nothing suggesting this is apparent in the raw data. However, this is the sequence one will obtain from Bangladeshi *P. elongatu*s when using standard barcode primers. It is unique for the species, serving as a DNA barcode.

Two specimens identified morphologically as *Chaca chaca* were analyzed, and turn out as distinct OBUs, 81 and 82. If representing distinct species, it cannot be decided at this time which one, if either, is the *Chaca chaca* of Hamilton (1822). There is one other potentially valid species described from Bengal, *Chaca lophioides* Cuvier & Valenciennes, 1832. Likewise, OBU 155–156 separates two specimens of *Chela cachius*, but there is no morphological support for this.

OBU 32 represents *Trichopsis vittata*. It is the first record of a feral population of an introduced species originating in South East Asia, which is not also an aquaculture species and the barcode is specific for the aquarium population derived from a specific region in Thailand^21^.

OBUs 83–85 represent three distinct species of *Olyra*, of which OBU 84 identified as *Olyra longicaudata*. Remaining two OBUs require more analysis before a final determination can be made.

OBUs 101–102 refer to two species of *Badis*, which are nearly indistinguishable morphologically, but have complementary geographical distribution and distinct *coi* sequences and were considered as cryptic sister species^20^.

OBUs 109–111 refer to *Pethia guganio*, a little studied small cyprinid species in the Ganga and Brahmaputra basins, but also present in the Karnafuli and Sangu drainages. The mPTP analysis recovered three OBUs initially identified as one species. *Pethia guganio* is probably a species complex that cannot be resolved taxonomically here. OBU 109, however, was distinguished by the presence of barbels, not otherwise known from *P. guganio*.

OBUs 126, *Osteobrama cotio*, and 127, *O*. cf. *cotio*, differed in *coi* and *mt-rnr2* sequences^22^. Samples from the Karnafuli and Sangu drainages were slightly different from those from the Meghna drainage, including published sequences from the Barak River. No morphological differences were found and they might be cryptic species^22^. Because *O. cotio* is a widespread species, it should be re-analyzed with inclusion of representation of the entire geographical distribution^23^. The mPTP analysis here recognizes the Meghna and Karnafuli + Sangu OBUs as two species, for which there is no morphological support.

OBU 136 was identified morphologically as *Labeo rohita*, but disagrees with the other *coi* sequence of *L. rohita*, and is identical with GenBank sequences identified as *Labeo dyocheilus* and *L. pangusia*, with a considerably different morphology. It may be a case of introgression in aquaculture conditions, but *L. dyocheilus* and *L pangusia* are large mountain species, unlikely candidates for hybridization in aquaculture.

OBU 152 is complicated, as it includes specimens of *Devario anomalus* with *D. aequipinnatus* mitochondrial genome^18^. *Devario coxi* is here nested with *D. aequipinnatus* from which it was diagnosed morphologically and by mitochondrial and nuclear genes^18^. Intra-OBU variation suggests inclusion of three distinct haplogroups.

OBUs 160–161 represent distinct OBUs, morphologically identified as *Danio rerio*. The two *D. rerio* mitotypes were already reported^23^ based on mitochondrial *cytochrome b* sequences and SNPs. Intraspecific variation in *D. rerio* needs additional analysis, but at this time there is no information to support species distinction.

OBUs 164–165, 171, 181–183, 187–88, 191–192 may represent cryptic species, or overlooked distinct species, or even populations of the same species excessively split by mPTP, but no indication was found of species differences.

OBU 177 contains a single small specimen from the Sangu drainage, similar to *Barilius barila* in the Meghna and Padma drainages, but assessment of its taxonomic status requires revision of *Barilius*.

OBU 185 represents a morphologically distinct species of *Lepidocephalichthys*.

OBU 202 represents two morphologically distinct, but similar species of *Schistura, S. sijuensis* from the Garo Hills, and a possibly undescribed species from the Sangu River. It seems likely that these two taxa may be sister species.

OBUs 186 and 189–190 represent *Lepidocephalichthys guntea* morphologically, but separate in three distinct OBUs with tentative morphological support. OBU 189 probably represents the true *L. guntea*.

OBU 202 combines two morphologically distinct taxa incompletely separated in two drainages.

OBUs 222–226 suggest (Table S1) five species of *Oryzias*, including *O. dancena*, but the tree indicates only four (Fig. 2). Only two species of *Oryzias* have been reported from Bangladesh^24^ and the *Oryzias* sequences maybe excessively split in the mPTP analysis.

The identification of OBU 240 presents a nomenclatural problem. Authorship and name of this species is uncertain. It was described as a new species, *Parambassis ornatus* by Geetakumari & Vishwanath, 2012^25^, and also, but with a different holotype, as *P. bistigmata* by Geetakumari, 2012^26^. The latter was published in May, 2012, but the date of publication of Geetakumari & Vishwanath’s book^25^ was not stated in the publication itself, only the year. The valid name, authorship and date needs more research.

All OBUs reported here, occur in freshwater, and the majority, 2001 (Table S1), may be considered to be exclusively or predominantly freshwater species. Classification by salinity level, however, is not straightforward. Species of the Gobiidae and Eleotridae*, Oryzias, Hyporhamphus limbatus*, may be common far inland, but may also be present in coastal waters and estuaries. The major component of the Bangladeshi fish fauna, however, is ostariophysan, and as such only contains freshwater species.

We obtained *coi* sequences from 149 out of the 237 freshwater fish species already recorded from Bangladesh^3^, including some euryhaline or estuarine species that are common in inland waters; and 116 out of 162 classed as strictly freshwater. Of the 237, 31 species represented taxa erroneously recorded for the country^27^, or sporadic occurrences, and several more may be considered to be marine rather than freshwater^5^. Nine of our sequenced species represented the 12 species of aquaculture or aquarium releases recorded from natural habitats. We further obtained 83 OBUs representing species that were not previously recorded for the country, including an estimated about 30 species that may be undescribed or not yet identified. Calculations of “unknowns” are uncertain insofar as they may represent previously misidentified species or synonyms to be re-validated.

The present study is weak in coverage in the southwestern and western parts of Bangladesh, principally the Sundarbans and a portion of the Ganga River and tributaries. Expectations of additional taxa from that area, are low, however. The coverage of the Chittagong Division, including Karnafuli and Sangu Rivers and the Cox’s Bazar region provides for considerable new ocurrences and new species^16,18–20^, but also strong affinity to the adjacent western Rakhine in Myanmar, e.g., in the shared distribution of *Laubuka tenella*^19^. Most of the samples, from the Meghna, Jamuna and Padma tributaries, reflect a common fish fauna with adjacent India, the samples from the Pyain River, draining the Garo Hills, provide a distinct representation belonging to the Eastern Himalaya Region^28^. Numerous fish species have been described from the Barak River in Manipur^29^, draining to the Surma and Meghna Rivers in Bangladesh. That fauna is not reflected in our material from Bangladesh, however. Also, the taxa shared with the Kaladan River^30^ are only species with very wide distribution in Bangladesh and India.

The above examples demonstrate that DNA barcoding is not trivial. Contamination, introgression, multiple mitochondrial DNA lineages, cryptic species, introductions and NUMTs do occur and may be difficult to detect. The examples in our data set are very limited, however. To this comes lack of modern revisions, and shortage of taxonomic expertise for qualified identification of voucher specimens. Very few of the South Asian fish genera or species groups have been revised by specialists, e.g., *Psilorhynchus*^31^, *Lepidocephalichthys*^32^, *Oryzias*^24^, *Paracanthocobitis*^33,34^, and *Sperata*^35^.

Our results provide relevant aspects on large-scale/country-wide DNA barcoding, end-use of barcodes, and limits of application of the DNA barcode concept, as detailed below.

The original expectations of DNA Barcoding were relatively modest: establishment of a database of distinct vouchered reference sequences against which unidentified sequences could be compared, and which would provide a name (identification) of the organism from which the unidentified sample was taken^8,15^. *coi*, however, has assumed additional roles, namely for building phylogenetic trees, and for species discovery^15^. Studies using DNA barcodes as a tool for species discovery^15,36^ were called “molecular parataxonomy” by Collins & Cruickshank^15^, but are perhaps better referred to as barcode or DNA prospecting. The principle of species recognition in DNA prospecting is based on the barcode gap convention^8,15^, and not on a species concept or a prior hypothesis of diagnostic characters or phylogenetic relationships^8^. The barcode gap, however, is a distance measure that only shows probability of reproductive isolation between two a priori determined samples. Consequently, we prefer the term suggested here, Operational Barcode Unit (OBU), which is akin to the ‘species-like units’ of Collins & Cruickshank^15^, for raw standard barcode *coi* sequences, with the caveat that identity may be the result of introgression. Dissimilar *coi* sequences indicate reduced interbreeding, which to differing degrees may also occur between demes or populations, not only between species, considered as independent evolutionary lineages^37^.

OBUs can be referred to without the need for relation to a particular species, but need specialist verification to serve as markers for species. The expectation of species delimitation methods is that they should provide automatic species recognition^8,37^, but a *coi* sequence distinct from others or not, is not a species marker, but only exhibiting levels of sequence divergence that uggestive species status^38^. As exemplified in our trees, units delineated by mPTP may conflict with species identification in several aspects, primarily dependent on the expertise of the determiner, but also reflecting introgression and sibling species, species concepts^37^, and availability of a taxonomic platform, requiring taxonomic validation using independent criteria.

DNA barcoding projects frequently report cryptic species as well as discovery of previously unrecognized species in fishes^39,40^. Commonly, the analysis stops there, and the recognition of cryptic species in DNA barcoding remains a by-product of molecular prospecting^39^. Putative cryptic species may represent widely different evolutionary processes, however^41^. Species may be considered cryptic because of insufficient morphological analysis, i.e., a shortcoming of morphological analysis and thus a temporary state of identification (provisionally cryptic^42^). Mitochondrial genome substitution due to hybridization in a particular population may also be mistaken for cryptic species. Consequently, “cryptic OBUs” require taxonomic assessment, and evolutionary analysis, and in the meantime remain ghost OBUs. Whereas many of our sequences cannot be linked to a particular species, only few cases of sibling or cryptic species were demonstrated in cyprinids and badids so far^18,20,22^, and the unidentified OBUs should not be assumed to represent cryptic species or undescribed species in the absence of taxonomic analysis. Nation-wide or regional barcoding surveys, have been proposed to accelerate DNA barcode coverage^43^, and some have been attempted, such as the one reported here. Another project was started in 2013 to barcode Swedish vertebrates^44^. Despite Sweden’s relatively species-poor and fully known vertebrate fauna, this barcode project does still not contain all species of vertebrates in Sweden (78.1% barcoded), or even fishes (72% barcoded)^44^. It seems unlikely that similar projects in species-rich tropical countries will be successful in achieving completeness. DNA barcodes are taxonomic statements. Each OBU translated to a species name expresses information that requires taxonomic expertise, sequencing and sampling skills, permanent voucher repositories, and constant revision of the taxonomic background. It also takes competence in using a proposed barcode, because similar OBUs may have different evolutionary explanations. We therefore consider that DNA barcoding should be done on a taxonomic, and not a geographical, or national basis.

Fish DNA barcodes serve a purpose, e.g., in the fishery industry for control of product authenticity and origin and for clearing potentially harmful imports, as explicit in several projects^10,11,43,45,46^. Hence, trade and consumption species are priority targets, and the fishery and biohazard agencies are most likely the institutions that have the means to sample and blast sequences against openly available digital sequence providers such as BOLD and GenBank, but which do not maintain a private DNA barcode library. In the context of such use, the overall critical factor is that users have an unknown tissue to check against a barcode library. The role of the end user is commonly underestimated or ignored in attempts at DNA barcoding, which then becomes barcode or DNA prospecting without any explicit end user.

This might mean less stress on taxonomic hair-splitting over the taxonomy of small-size fish species, which is relevant for many of the species in our analysis. In southern Asia, however small-size fishes are a significant component of the nutrition^47^, and identification protocols for small-size fish species may be relevant for monitoring purposes.

In conclusion, we have lifted some of the curtain to Bangladeshi fish *coi* sequences in the present report, but also exposed some of the challenges and critical aspects of DNA barcoding from a taxonomic perspective. It suggests that the decisive component of DNA barcoding, are end-user capacity, and taxonomic revision dealing with identification, nomenclature, species delimitation, introgression, multiple mitochondrial lineages, and more, We have been successful in generating numerous *coi* sequences and correlate them with species, but it turns out that was only the start of the barcoding effort.

## Methods

### Sampling

Fish **s**pecimens were collected using a 5 mm mesh size beach seine, or, for commercial species, by purchase directly from fishermen or at local fish markets. Specimens were obtained from 150 collecting events and additional ad hoc sampling including both markets and natural habitats (Fig. 1). The result was 3278 whole specimens yielding 2514 tissue samples (whole specimens or fin clips) with considerable redundance. Based on morphological criteria and representation of different river basins, 697 samples from Bangladesh were selected for sequencing. *coi* sequencing failed in two the two specimens svailable of *Johnius coitor* (Sciaenidae) and the single specimen of *Taenioides cirratus* Gobiidae), resulting in 692 specimens for analysis.

Whole fish or fin clips were stored in 95% ethanol at −80 °C. Fin-clipped whole specimens and excess specimens for morphological analyses were fixed in 10% formalin and eventually transferred to 70% ethanol for permanent storage. Tissue samples and voucher specimens were deposited in the fish collection of the Swedish Museum of Natural History (NRM), and the Department of Zoology, University of Dhaka (DU).

### Taxonomic identification

Specimens were sorted and identified morphologically before sequencing. Identification was made by using comparative material identified by experts in the NRM collection, specialist publications, and monographs. Several experts assisted in determinations based on high-resolution photographs, or with comments on taxonomic status.

*The FISH-BOL collaborator’s* protocol^9^ provides a graded classification of determination quality, which we employ here, with the modification that we report confidence per specimens in the respective OBU, and not per OBU or individual sequence, and disregard their levels 4–5 which do not produce a species determination at all.

“Level 1: highly reliable identification-specimen identified by (1) an internationally recognized authority of the group, or (2) a specialist that is presently studying or has reviewed the group in the region in question”.

“Level 2: identification made with high degree of confidence at all levels-specimen identified by a trained identifier who had prior knowledge of the group in the region or used available literature to identify the specimen”.

Level 3: (modified) “identification made with high confidence to genus but less so to species—specimen identified by (1) a trained identifier who was confident of its generic placement but did not substantiate their species identification using the literature”.

### Dataset limitation

The dataset reported is limited to the data assembled by the project, 2014–2016.

### DNA barcode sequencing

Approximately 1 mm^3^ tissue was taken from the alcohol preserved fish or fin clip, and DNA extracted using either a Kingfisher Duo (Thermo Scientific) DNA extraction robot, with recommended protocol, or DNEasy Blood & Tissue Kit (QIAGEN) spin columns, with recommended protocol.

The standard barcode sequence, 655 bp from the 5′ end of *coi*, was sequenced with the primers Fish-F1, Fish-F2, Fish-R1, Fish-R2^48^

PCR was performed using illustra PuReTaq RTG PCR Beads (GE Healthcare), with 2 µL DNA extract, 0.5 µL of each primer and adding water for a 25 µL reaction PCR cycling was: 94 °C 5′, 35 * (54 °C 30″, 72 °C 30″), 72 °C 7′. In a few cases where molecular and morphological analyses conflicted (i.e., where there was reason to suspect hybridization or that the *coi* sequence was a nuclear pseudogene), an additional mitochondrial gene (*mt-rnr2*) and a nuclear gene *rag1* exon 3) were sequenced.

*mt-rnr2* was sequenced using the primers 16S_arLm2 (CCTCGCCTGTTTACCAAAAACA) and 16S_brHm (CTCCGGTCTGAACTCAGATCACGT), with PCR cycling as for COI except with 57 °C annealing temperature. *rag1* exon 3 was sequenced using the primers R1_Dan1f (TGGCCATAAGGGTMAACAC) and R1_4078r (TGAGCCTCCATGAACTTCTGAAGRTAYTT), with PCR cycling as for *coi*.

The PCR product was cleaned by adding 5 μL of a mix of 20% Exonuclease I (EXO) and 80% FastAP Thermosensitive Alkaline Phosphatase (Fermentas) to each 25 μl reaction, and incubated at 37 °C for 30 minutes, then heated to 80 °C for 15 minutes.

The cleaned PCR product was sent to Macrogen (Amstelween, The Netherlands) for sequencing. Reads were assembled and proofread in Geneious version 10^49^, and a GenBank BLAST (megablast) search of the GenBank *nr* database was used as a first screening for misidentified or contaminated sequences. To detect and visualize contaminations and problems with identification, sequences were aligned in Geneious and phylogenetic hypotheses were constructed in MrBayes version 3.2^50^, with the following settings: GTR + I + G model, 15 million generations, with the first 25% discarded as burn-in, then sampled every 1000 generations. Convergence was checked with Tracer^51^.

Sequences which passed quality control were uploaded to the Barcode of Life Database (BOLD^52^ and published to GenBank^53^. A total of 694 barcode-compliant sequences were produced on material from Bangladesh. An additional two sequences from Bangladeshi specimen sequences were not barcode compliant, due to being pseudogenes (NUMTs).

### Species delimitation

OBU detection and delimitation by multi-rate Poisson Tree Process method was investigated based on the Bayesian tree, using mPTP version^54^, with the following settings: 15 separate runs, minimum branch length estimated from alignment (0.0053), one lambda for the coalescent, starting with random species assignments, 40 million generations, the first 20 milllion generations discarded as burn-in, then sampled every 50 thousand generations. Convergence was checked by examining mPTP’s log files.

Single Most Likely Trees were constructed using the RAxML^55^ and PHYML^56^ plug-ins for Geneious. RAxML analysis was performed with GTR + G model, rapid hill climbing algorithm, seven starting trees, parsimony random seed 1, and Maximum Likelihood search convergence criterion. PHYML analysis was performed with GTR + I + G model, proportion of invariable sites and gamma estimated in analysis, NNI topology search. The mPTP analysis was performed as for the Bayesian.

### Terminology

Gene names and symbols follow ZFIN nomenclature conventions^57^, except that we use here the universally common synonym *coi* for the *mt-co1*). We reserve the term DNA barcode for a ~655 bp Folmer region *coi* sequence from a vouchered specimen identified as a particular species, i.e., quality levels 1–3^9^. We use barcoding/barcoded for the process/accomplishment of establishing a DNA barcode for a particular species. We use the short *coi* for the standard DNA barcode part of the *mt-co1* gene unless stated otherwise. We do not consider *coi* sequences as necessarily representing species or even taxonomic units. Each such sequence needs further analysis to assess it status. We propose the neutral term OBU (Operational Barcode Unit), as descriptor of presumed species level terminals found in an analysis of *coi* using multiple sequences, or a single unidentified specimen. Ideally, an OBU will be found eventually to represent a distinct species, and its sequence serve as a DNA barcode. We designate as ghost OBU a distinct standard DNA barcode sequence, or group of standard DNA barcode sequences, that cannot be identified as pertaining to any known species. Ghost OBUs may represent unidentified species, new species, NUMTs, within-species haplogroups, or bad sequence reads.

### Ethics statement

Specimens were already available in museum collections, purchased from fishermen or at markets; or collected in the wild using a beach seine or hand net and euthanized through immersion in buffered tricaine-methanesulphonate (MS 222) until cessation of opercular movements plus an additional 30 minutes, following the protocol in permits from the Swedish Environmental Protection Agency (dnr 412-7233-08 Nv) and the Stockholm Ethical Committee of the Swedish Board of Agriculture (dnr N 85/15). Collecting in Bangladesh was conducted under a permit to the University of Dhaka, and approval of the Ethical Review Committee, Faculty of Biological Sciences, University of Dhaka.

## Supplementary information

Building a DNA barcode library for the freshwater fishes of Bangladesh

High Resolution Figure 2

High Resolution Figure 3

## Data Availability

All sequence and associated voucher data are available from BOLD and GenBank. Voucher metadata are available in Supplementary Information.
