# Publisher Correction: Building a DNA barcode library for the freshwater fishes of Bangladesh

**DOI:** 10.1038/s41598-020-58810-0

**Published:** 2020-01-29

**Authors:** Md. Mizanur Rahman, Michael Norén, Abdur Rob Mollah, Sven O. Kullander

**Affiliations:** 1 University of Dhaka, Department of Zoology, Dhaka, Dhaka 1000 Bangladesh; 2 Swedish Museum of Natural History, Department of Zoology, SE-104 05, Stockholm, Sweden

Correction to: *Scientific Reports* 10.1038/s41598-019-45379-6, published online 28 June 2019

This Article contains low resolution versions of Figures 2 and 3. High resolution versions have now been added as Supplementary Information files that accompany the Article.

