# Supplementary material for: Building a DNA barcode library for the freshwater fishes of Bangladesh
Source: Sci Rep. 2019 Jun 28;9:9382. doi: 10.1038/s41598-019-45379-6 (PMC6599201; doi:10.1038/s41598-019-45379-6)
Supplement: Supplementary file 1 — Building a DNA barcode library for the freshwater fishes of Bangladesh [file 41598_2019_45379_MOESM1_ESM.pdf]

SUPPLEMENTARY MATERIAL

Building a DNA barcode library for the freshwater fishes of Bangladesh

Md. Mizanur Rahman[1], Michael Norén[2], Abdur Rob Mollah[1], Sven O. Kullander[2]*
[1]University of Dhaka, Department of Zoology, Dhaka, Dhaka-1000, Bangladesh
[2]Swedish Museum of Natural History, Department of Zoology, SE-104 05 Stockholm, Sweden
*Corresponding author

Table S1. Operational Barcode Units (OBUs detected by mPTP analysis, ordered by OBU number. N is number of samples. ID level is level of confidence in species determination (1 highest; 0 none). Habitat abbreviations: Br, brackish; Cata, catadromous; Fresh, freshwater; M, marine.

OBU	Family	Taxonomic correlate	N	ID level	Native	Habitat
1	Osphromenidae	*Trichogaster lalius*	5	1	YES	Fresh
2	Osphromenidae	*Trichogaster chuna*	3	1	YES	Fresh
3	Osphromenidae	*Trichogaster fasciata*	3	1	YES	Fresh
4	Gobiidae	*Glossogobius aureus*	3	3	YES	Fresh
5	Gobiidae	*Glossogobius giuris* (1)	5	2	YES	Fresh_Br
6	Gobiidae	*Glossogobius giuris* (2)	1	0	YES	Fresh_Br
7	Gobiidae	*Acentrogobius viridipunctatus*	1	0	YES	Br_M
8	Gobiidae	*Stigmatogobius sadanundio*	2	3	YES	Br__M
9	Eleotridae	*Butis humeralis*	1	3	YES	Br__M
10	Gobiidae	*Brachygobius nunus*	3	3	YES	Br__M
11	Gobiidae	*Brachygobius nunus* Mitotype 2	2	3	YES	Br__M
12	Gobiidae	*Apocryptes bato*	4	3	YES	Br__M
13	Gobiidae	*Odontamblyopus rubicundus*	2	3	YES	Br__M

14	Gobiidae	*Pseudapocryptes elongatus*	3	3	YES	Br__M
15	Gobiidae	*Parapocryptes serperaster*	2	3	YES	Br__M
16	Gobiidae	*Boleophthalmus boddarti*	2	3	YES	Br__M
17	Gobiidae	*Periophthalmus barbarus*	2	3	YES	Br__M
18	Gobiidae	*Awaous grammepomus*	3	3	YES	Br__M
19	Gobiidae	*Gobiopterus chuno*	1	3	YES	Br__M
20	Eleotridae	*Eleotris melanosoma*	5	3	YES	Br__M
21	Eleotridae	*Eleotris lutea*	1	3	YES	Br__M
22	Eleotridae	*Bunaka gyrinoides*	1	3	YES	Br__M
23	Eleotridae	*Hypseleotris cyprinoides*	1	3	YES	Br__M
24	Eleotridae	*Ophiocara porocephala*	1	3	YES	Br__M
25	Syngnathidae	*Microphis cuncalus*	2	3	YES	Fresh
26	Syngnathidae	*Ichthyocampus carce*	1	3	YES	Fresh
27	Tetraodontidae	*Leiodon cutcutia*	3	2	YES	Fresh
28	Tetraodontidae	*Takifugu oblongus*	1	3	YES	Br_M
29	Tetraodontidae	*Dichotomyctere fluviatilis*	1	3	YES	Br_M
30	Poeciliidae	*Gambusia affinis*	1	2	YES	Fresh
31	Aplocheilidae	*Aplocheilus panchax*	7	2	YES	Fresh
32	Osphromenidae	*Trichopsis vittata*	5	1	NO	Fresh
33	Gobiidae	*Pseudogobiopsis oligactis*	2	3	YES	Br_M
34	Heteropneustidae	*Heteropneustes fossilis*	4	3	YES	Fresh
35	Clariidae	*Clarias magur*	3	3	YES	Fresh
36	Horabagridae	*Pachypterus atherinoides*	6	3	YES	Fresh
37	Bagridae	*Hemibagrus menoda*	2	3	YES	Fresh
38	Bagridae	*Mystus tengara*	4	3	YES	Fresh
39	Bagridae	*Mystus carcio*	4	3	YES	Fresh
40	Bagridae	*Mystus bleekeri*	2	3	YES	Fresh
41	Bagridae	*Mystus gulio*	6	3	YES	Fresh
42	Bagridae	*Mystus cavasius*	3	3	YES	Fresh
43	Bagridae	*Sperata aorella*	3	3	YES	Fresh
44	Bagridae	*Sperata seenghala*	1	3	YES	Fresh

45	Bagridae	*Sperata aor*	3	3	YES	Fresh
46	Ailiidae	*Clupisoma garua*	6	3	YES	Fresh
47	Ailiidae	*Eutropiichthys vacha*	4	3	YES	Fresh
48	Ailiidae	*Eutropiichthys murius*	1	3	YES	Fresh
49	Ailiidae	*Silonia silondia*	2	3	YES	Fresh
50	Ailiidae	*Ailia coila*	3	3	YES	Fresh
51	Pangasiidae	*Pangasius pangasius*	1	3	YES	Fresh
52	Pangasiidae	*Pangasianodon hypophthalmus*	1	3	NO	Fresh
53	Sisoridae	*Glyptothorax telchitta*	5	3	YES	Fresh
54	Sisoridae	*Glyptothorax botius*	1	3	YES	Fresh
55	Sisoridae	*Glyptothorax* cf *trilineata*	2	0	YES	Fresh
56	Sisoridae	*Bagarius bagarius*	1	0	YES	Fresh
57	Sisoridae	*Pseudolaguvia* sp3	2	0	YES	Fresh
58	Sisoridae	*Pseudolaguvia ribeiroi*	2	0	YES	Fresh
59	Sisoridae	*Pseudolaguvia* sp4	1	0	YES	Fresh
60	Sisoridae	*Pseudolaguvia* sp1	1	0	YES	Fresh
61	Sisoridae	*Pseudolaguvia shawi*	1	1	YES	Fresh
62	Sisoridae	*Pseudolaguvia* sp	2	0	YES	Fresh
63	Sisoridae	*Pseudolaguvia fucosa*	1	1	YES	Fresh
64	Sisoridae	*Erethistes jerdoni*	1	3	YES	Fresh
65	Sisoridae	*Erethistes pusillus*	4	3	YES	Fresh
66	Sisoridae	*Erethistoides* sp	2	0	YES	Fresh
67	Sisoridae	*Nangra assamensis*	1	1	YES	Fresh
68	Sisoridae	*Nangra bucculenta*	1	3	YES	Fresh
69	Sisoridae	*Gagata cenia*	4	3	YES	Fresh
70	Sisoridae	*Gagata gagata*	3	3	YES	Fresh
71	Sisoridae	*Gogangra laevis*	5	1	YES	Fresh
72	Amblycipitidae	*Amblyceps mangois*	6	3	YES	Fresh
73	Siluridae	*Ompok bimaculatus*	2	3	YES	Fresh
74	Siluridae	*Ompok pabo*	2	3	YES	Fresh
75	Siluridae	*Ompok pabda*	2	3	YES	Fresh

76	Siluridae	*Pterocryptis barakensis*	2	0	YES	Fresh
77	Siluridae	*Wallago attu*	1	3	YES	Fresh
78	Ritaidae	*Rita rita*	2	3	YES	Fresh
79	Bagridae	*Batasio batasio*	3	3	YES	Fresh
80	Bagridae	*Rama chandramara*	4	3	YES	Fresh
81	Chacidae	*Chaca chaca* (1)	1	0	YES	Fresh
82	Chacidae	*Chaca chaca* (2)	1	0	YES	Fresh
83	Bagridae	*Olyra* sp	6	0	YES	Fresh
84	Bagridae	*Olyra longicaudata*	3	1	YES	Fresh
85	Bagridae	*Olyra* sp1	2	0	YES	Fresh
86	Loricariidae	*Pterygoplichthys* sp	3	3	NO	Fresh
87	Channidae	*Channa punctata*	5	2	YES	Fresh
88	Channidae	*Channa gachua* (1)	1	0	YES	Fresh
89	Channidae	*Channa gachua* (2)	1	0	YES	Fresh
90	Channidae	*Channa* sp cf *gachua*	1	0	YES	Fresh
91	Channidae	*Channa gachua* (3)	4	0	YES	Fresh
92	Channidae	*Channa marulius*	2	1	YES	Fresh
93	Channidae	*Channa striata* (1)	2	0	YES	Fresh
94	Channidae	*Channa striata* (2)	2	0	YES	Fresh
95	Nandidae	*Nandus nandus*	2	1	YES	Fresh
96	Cynoglossidae	*Cynoglossus cynoglossus*	2	0	YES	Br_M
97	Polynemidae	*Polynemus paradiseus*	2	1	YES	Br_M
98	Latidae	*Lates calcarifer*	1	0	YES	Br_M
99	Soleidae	*Brachirus orientalis*	2	0	YES	Br_M
100	Badidae	*Badis badis*	11	1	YES	Fresh
101	Badidae	*Badis pallidus*	10	1	YES	Fresh
102	Badidae	*Badis chittagongis*	6	1	YES	Fresh
103	Badidae	*Badis rhabdotus*	2	1	YES	Fresh
104	Badidae	*Dario kajal*	2	1	YES	Fresh
105	Mastacembelidae	*Macrognathus pancalus*	3	2	YES	Fresh
106	Mastacembelidae	*Macrognathus aral*	2	2	YES	Fresh

107	Mastacembelidae	*Mastacembelus armatus*	6	0	YES	Fresh
108	Chaudhuriidae	*Pillaia* sp	2	0	YES	Fresh
109	Cyprinidae	*Pethia* sp aff *guganio*	2	0	YES	Fresh
110	Cyprinidae	*Pethia guganio*	4	3	YES	Fresh
111	Cyprinidae	*Pethia guganio*	1	3	YES	Fresh
112	Cyprinidae	*Pethia gelius*	4	1	YES	Fresh
113	Cyprinidae	*Pethia conchonius*	6	1	YES	Fresh
114	Cyprinidae	*Pethia rutila*	4	0	YES	Fresh
115	Cyprinidae	*Pethia ticto*	1	1	YES	Fresh
116	Cyprinidae	*Pethia phutunio*	2	1	YES	Fresh
117	Cyprinidae	*Garra* sp SHERPUR	1	0	YES	Fresh
118	Cyprinidae	*Garra* sp cf *koladynensis*	1	0	YES	Fresh
119	Cyprinidae	*Garra* sp MEGHNA	1	0	YES	Fresh
120	Cyprinidae	*Garra vittatula*	4	2	YES	Fresh
121	Cyprinidae	*Garra mini*	6	1	YES	Fresh
122	Cyprinidae	*Garra dampaensis*	3	0	YES	Fresh
123	Cyprinidae	*Puntius terio*	3	1	YES	Fresh
124	Cyprinidae	*Puntius chola*	2	1	YES	Fresh
125	Cyprinidae	*Puntius sophore*	4	1	YES	Fresh
126	Cyprinidae	*Osteobrama* cf *cotio*	2	0	YES	Fresh
127	Cyprinidae	*Osteobrama cotio*	2	1	YES	Fresh
128	Cyprinidae	*Systomus sarana*	2	3	YES	Fresh
129	Cyprinidae	*Barbonymus gonionotus*	2	1	NO	Fresh
130	Cyprinidae	*Oreichthys cosuatis*	2	1	YES	Fresh
131	Cyprinidae	*Labeo rohita*	1	1	YES	Fresh
132	Cyprinidae	*Labeo catla*	1	1	YES	Fresh
133	Cyprinidae	*Labeo angra*	1	3	YES	Fresh
134	Cyprinidae	*Labeo calbasu*	3	2	YES	Fresh
135	Cyprinidae	*Labeo gonius*	2	2	YES	Fresh
136	Cyprinidae	Labeonini	1	0	YES	Fresh
137	Cyprinidae	*Cirrhinus cirrhosus*	3	1	YES	Fresh

138	Cyprinidae	*Labeo boggut*	3	3	YES	Fresh
139	Cyprinidae	*Labeo boga*	2	3	YES	Fresh
140	Cyprinidae	*Gymnostomus ariza*	4	1	YES	Fresh
141	Cyprinidae	*Cyprinus carpio*	2	3	NO	Fresh
142	Cyprinidae	*Tariqilabeo latius*	3	3	YES	Fresh
143	Cyprinidae	*Neolissochilus stracheyi*	3	3	YES	Fresh
144	Cyprinidae	*Chagunius chagunio*	2	2	YES	Fresh
145	Cyprinidae	*Ctenopharyngodon idella*	2	0	NO	Fresh
146	Cyprinidae	*Mylopharyngodon piceus*	1	0	NO	Fresh
147	Cyprinidae	*Hypophthalmichthys molitrix*	1	0	NO	Fresh
148	Cyprinidae	*Hypophthalmichthys nobilis*	1	0	NO	Fresh
149	Cyprinidae	*Rasbora rasbora*	3	1	YES	Fresh
150	Cyprinidae	*Amblypharyngodon mola*	6	1	YES	Fresh
151	Cyprinidae	*Rasbora daniconius*	4	1	YES	Fresh
152	Cyprinidae	*Devario aequipinnatus*	12	1	YES	Fresh
153	Cyprinidae	*Devario anomalus*	1	1	YES	Fresh
154	Cyprinidae	*Devario devario*	3	1	YES	Fresh
155	Cyprinidae	*Chela cachius* (1)	1	0	YES	Fresh
156	Cyprinidae	*Chela cachius* (2)	1	0	YES	Fresh
157	Cyprinidae	*Laubuka laubuca*	2	1	YES	Fresh
158	Cyprinidae	*Laubuka tenella*	4	1	YES	Fresh
159	Cyprinidae	*Danio annulosus*	4	1	YES	Fresh
160	Cyprinidae	*Danio rerio*	3	1	YES	Fresh
161	Cyprinidae	*Danio* cf *rerio*	9	0	YES	Fresh
162	Cyprinidae	*Danio* sp BANGLADESH	2	0	YES	Fresh
163	Cyprinidae	*Esomus danrica*	6	1	YES	Fresh
164	Psilorhynchidae	*Psilorhynchus nudithoracius*	4	0	YES	Fresh
165	Psilorhynchidae	*Psilorhynchus sucatio* (1)	1	0	YES	Fresh
166	Psilorhynchidae	*Psilorhynchus sucatio* (2)	4	0	YES	Fresh
167	Psilorhynchidae	*Psilorhynchus rahmani*	2	2	YES	Fresh
168	Psilorhynchidae	*Psilorhynchus balitora*	2	0	YES	Fresh

169	Cyprinidae	*Salmostoma sardinella*	3	3	YES	Fresh
170	Cyprinidae	*Salmostoma phulo*	6	3	YES	Fresh
171	Cyprinidae	*Salmostoma* sp KUSHIYARA	6	0	YES	Fresh
172	Cyprinidae	*Securicula gora*	1	3	YES	Fresh
173	Cyprinidae	*Salmostoma bacaila*	5	3	YES	Fresh
174	Cyprinidae	*Cabdio morar*	4	1	YES	Fresh
175	Cyprinidae	*Opsarius bendelisis*	6	3	YES	Fresh
176	Cyprinidae	*Barilius barila* (1)	4	3	YES	Fresh
177	Cyprinidae	*Barilius barila (2)*	1	3	YES	Fresh
178	Cyprinidae	*Opsarius barna*	8	3	YES	Fresh
179	Cyprinidae	*Opsarius tileo*	4	3	YES	Fresh
180	Cyprinidae	*Raiamas bola*	1	3	YES	Fresh
181	Cobitidae	*Lepidocephalichthys* sp	2	0	YES	Fresh
182	Cobitidae	*Lepidocephalichthys goalparensis*	4	0	YES	Fresh
183	Cobitidae	*Lepidocephalichthys caudofurcatus*	3	0	YES	Fresh
184	Cobitidae	*Lepidocephalichthys irroratus*	2	0	YES	Fresh
185	Cobitidae	*Lepidocephalichthys* sp1	1	0	YES	Fresh
186	Cobitidae	*Lepidocephalicthys* sp3	2	0	YES	Fresh
187	Cobitidae	*Lepidocephalichthys annandalei* (1)	2	0	YES	Fresh
188	Cobitidae	*Lepidocephalichthys annandalei* (2)	1	0	YES	Fresh
189	Cobitidae	*Lepidocephalichthys guntea*	3	0	YES	Fresh
190	Cobitidae	*Lepidocephalichthys sp2*	5	0	YES	Fresh
191	Cobitidae	*Neoeucirrhichthys maydelli* (1)	1	0	YES	Fresh
192	Cobitidae	*Neoeucirrhichthys maydelli* (2)	2	0	YES	Fresh
193	Cobitidae	*Pangio pangia*	4	0	YES	Fresh
194	Cobitidae	*Cantophrys gongota*	2	2	YES	Fresh
195	Balitoridae	*Balitora brucei*	3	2	YES	Fresh
196	Botiidae	*Botia rostrata*	2	2	YES	Fresh
197	Botiidae	*Botia lohachata*	2	2	YES	Fresh
198	Botiidae	*Botia dario*	3	2	YES	Fresh
199	Nemacheilidae	*Schistura scaturigina*	5	0	YES	Fresh

200	Nemacheilidae	*Schistura corica*	5	0	YES	Fresh
201	Nemacheilidae	*Schistura aff corica*	1	0	YES	Fresh
202	Nemacheilidae	*Schistura sijuensis*	5	0	YES	Fresh
203	Nemacheilidae	*Schistura savona*	5	3	YES	Fresh
204	Nemacheilidae	*Schistura* sp1	2	0	YES	Fresh
205	Nemacheilidae	*Paracanthocobitis abutwebi*	6	3	YES	Fresh
206	Nemacheilidae	*Paracanthocobitis mackenziei*	2	3	YES	Fresh
207	Clupeidae	*Gonialosa manmina*	7	0	YES	Br_M
208	Clupeidae	*Gudusia chapra*	3	0	YES	Br_M
209	Clupeidae	*Tenualosa ilisha*	5	0	YES	Br_M
210	Clupeidae	*Hilsa kelee*	1	0	YES	Br_M
211	Clupeidae	*Corica soborna*	4	0	YES	Br_M
212	Engraulididae	*Coilia dussumieri*	1	0	YES	Br_M
213	Engraulididae	*Setipinna phasa*	1	0	YES	Br_M
214	Pristigasteridae	*Ilisha melastoma*	1	0	YES	Br_M
215	Sillaginidae	*Sillaginopsis domina*	2	0	YES	Br_M
216	Megalopidae	*Megalops cyprinoides*	1	1	YES	Br_M
217	Anguillidae	*Anguilla bengalensis*	5	0	YES	Cata
218	Ophichthyidae	*Pisodonophis boro*	5	0	YES	Fresh
219	Notopteridae	*Notopterus notopterus*	2	1	YES	Fresh
220	Notopteridae	*Chitala chitala*	1	0	YES	Fresh
221	Synbranchidae	*Monopterus cuchia*	2	0	YES	Fresh
222	Adrianichthyidae	*Oryzias* sp1	2	0	YES	Fresh
223	Adrianichthyidae	*Oryzias* sp4	1	0	YES	Fresh
224	Adrianichthyidae	*Oryzias* sp3	2	0	YES	Fresh
225	Adrianichthyidae	*Oryzias* sp2	2	0	YES	Fresh
226	Adrianichthyidae	*Oryzias dancena*	3	0	YES	Fresh
227	Gerreidae	*Gerres setifer*	3	0	YES	Br_M
228	Zenarchopteridae	*Dermogenys burmanica*	8	0	YES	Fresh
229	Belonidae	*Xenentodon cancila*	3	3	YES	Fresh
230	Hemiramphidae	*Hyporhamphus limbatus*	4	0	YES	Br_M

231	Mugilidae	*Liza melanoptera*	1	0	YES	Br_M
232	Mugilidae	*Planiliza parsia*	2	0	YES	Br_M
233	Mugilidae	*Minimugil cascasia*	2	0	YES	Br_M
234	Mugilidae	*Rhinomugil corsula*	4	0	YES	Br_M
235	Terapontidae	*Terapon jarbua*	2	0	YES	Br_M
236	Ambassidae	*Parambassis lala*	6	0	YES	Fresh
237	Ambassidae	*Chanda nama*	6	0	YES	Fresh
238	Ambassidae	*Parambassis ranga*	11	0	YES	Fresh
239	Ambassidae	*Parambassis baculis*	1	0	YES	Fresh
240	Ambassidae	*Parambassis bistigmata*	1	0	YES	Fresh
241	Cichlidae	*Oreochromis niloticus*	2	3	NO	Fresh
242	Anabantidae	*Anabas cobojius*	3	0	YES	Fresh
243	Anabantidae	*Anabas testudineus*	1	0	YES	Fresh

TABLE S2. Voucher metadata as catalogued in NRM and DU. Vouchers catalogued in NRM may have been or will be transferred to DU (usually with 9000 numbers).
Identifications and metadata are subject to revision and may change.

OBU	Family	Taxonomic correlate	GenBank Accn N	Repository	Catalogue No	Identified by	Tissue No	Division	Locality	Drainage	Latitude	Longitude	Date
1	Osphromemidae	*Trichogaster lalius*	MK572623	DU	6205	Rahman, MM	6205	Mymenshing	Sherpur market	Jamuna	25.0229	90.01728	2015-03-21
1	Osphronemidae	*Trichogaster lalius*	MK572624	NRM	66166	Kullander, SO	11962	Dhaka	Meghna River at Ashuganj Point	Meghna	24.0463	91.00562	20 Mar 2016
1	Osphronemidae	*Trichopsis lalius*	MK572625	NRM	66752	Kullander, SO	10377	Chittagong	Roadside ditch in Sultanpur	Karnafuli	22.5422	91.8997	29 Nov 2014
1	Osphronemidae	*Trichogaster lalius*	MK572627	NRM	66595	Kullander, SO	9851	Dhaka	Fish market in Shonbari, Sreenagar	Padma	23.544	90.29635	2 Dec 2014
1	Osphronemidae	*Trichogaster lalius*	MK572626	NRM	66647	Kullander, SO	9938	Chittagong	Borokal, small stream drainging pool below Shuvolong waterfall	Karnafuli	22.7143	92.2477	28 Nov 2014
2	Osphronemidae	*Trichogaster chuna*	MK572618	NRM	66763	Kullander, SO	10385	Chittagong	Roadside ditch in Sultanpur	Karnafuli	22.5422	91.8997	29 Nov 2014
2	Osphronemidae	*Trichogaster chuna*	MK572619	NRM	66762	Kullander, SO	10384	Chittagong	Roadside ditch in Sultanpur	Karnafuli	22.5422	91.8997	29 Nov 2014
2	Osphronemidae	*Trichogaster chuna*	MK572617	NRM	66182	Kullander, SO	11892	Sylhet	Fenchuganj, roadside ditch in Garuli floodplain, 4 km south of Sylhet	Meghn	24.6945	91.94553	22 Mar 2016
3	Osphronemidae	*Trichogaster fasciata*	MK572622	NRM	68318	Kullander, SO	12205	Sylhet	Kushiyara River drainage, Chatul floodplain, E of Golapganj	Meghna	24.843	92.05717	23 Mar 2016
3	Osphronemidae	*Trichogaster fasciata*	MK572620	NRM	66445	Kullander, SO	11164	Chittagong	Roadside ditch in Sultanpur	Karnafuli	22.5422	91.8997	29 Nov 2014
3	Osphronemidae	*Trichogaster fasciata*	MK572621	NRM	67019	Kullander, SO	11056	Dhaka	Local fish market in Dhaka				1 Dec 2014
4	Gobiidae	*Glossogobius aureus*	MK572222	DU	9036	Kullander, SO	9036	Mymenshing	Batpara point Jamuna River	Jamuna			
4	Gobiidae	*Glossogobius aureus*	MK572220	NRM	67954	Kullander, SO	11274	Chittagong	Donumia Stream, 10 km north of Teknaf town, 70 km south of Cox's Bazar	Naf	20.9233	92.26298	9 May 2015
4	Gobiidae	*Glossogobius aureus*	MK572221	NRM	67042	Kullander, SO	10268	Chittagong	Halda River at Madinagut Point	Karnafuli	22.4291	91.88801	30 Nov 2014
5	Gobiidae	*Glossogobius giuris*	MK572224	NRM	67814	Kullander, SO	10826	Dhaka	Padma River near Srinagar	Padma			2 Dec 2014
5	Gobiidae	*Glossogobius giuris*	MK572228	NRM	69253	Kullander, SO	11545	Chittagong	Piyain River at Jaflong	Meghna	25.1844	92.01642	24 Mar 2016
5	Gobiidae	*Glossogobius giuris*	MK572227	NRM	66198	Kullander, SO	11948	Sylhet	River at Bisnakandi, coming from Meghalaya, tributary to Meghna River	Meghna	25.1592	91.89299	25 Mar 2016
5	Gobiidae	*Glossogobius giuris*	MK572225	NRM	66692	Kullander, SO	9954	Dhaka	Turag River at Kamarpare, near Dhaka city	Padma	23.8985	90.38438	1 Dec 2014
5	Gobiidae	*Glossogobius giuris*	MK572226	NRM	66567	Kullander, SO	9787	Chittagong	Woagga Stream, 4 km W of Kaptai National Park	Karnafuli	22.5091	92.14182	28 Nov 2014
6	Gobiidae	*Glossogobius giuris*	MK572223	NRM	67413	Kullander, SO	10752	Chittagong	Barochora stream, 4 km south of Cox's Bazar		21.3959	92.00372	8 May 2015
7	Gobiidae	*Acentrogobius viridipunctatus*	MK572007	DU	6128	Norén, M	6128	Chittagong	Hatia	Meghna	21.5833	92.01666	2015-03-02
8	Gobiidae	*Stigmatogobius sadanundio*	MK572604	NRM	67043	Kullander, SO	10267	Chittagong	Halda River at Madinagut Point	Karnafuli	22.4291	91.88801	30 Nov 2014
8	Gobiidae	*Stigmatogobius sadanundio*	MK572605	NRM	67199	Kullander, SO	10556	Chittagong	Lower part of small stream tributary to Naf River, about 3 km north of Teknaf	Naf	20.8986	92.27865	10 May 2015
9	Eleotridae	*Butis humeralis*	MK572083	DU	6123	Kullander, SO	6123	Khulna	Shamnagar, Sundarban	Sundarban	21.9748	89.19022	2014-12-14
10	Gobiidae	*Brachygobius nunus*	MK572078	NRM	66992	Kullander, SO	10095	Dhaka	Padma River near Srinagar	Padma			2 Dec 2014
10	Gobiidae	*Brachygobius nunus*	MK572079	NRM	66554	Kullander, SO	9777	Chittagong	Halda River at Madinagut Point	Karnafuli	22.4291	91.88801	30 Nov 2014
10	Gobiidae	*Brachygobius nunus*	MK572077	NRM	66556	Kullander, SO	9779	Chittagong	Halda River at Madinagut Point	Karnafuli	22.4291	91.88801	30 Nov 2014
11	Gobiidae	*Brachygobius nunus*	MK572081	NRM	66699	Kullander, SO	9944	Dhaka	Turag River at Kamarpare, near Dhaka city	Padma	23.8985	90.38438	1 Dec 2014
11	Gobiidae	*Brachygobius nunus*	MK572080	NRM	66700	Kullander, SO	9945	Dhaka	Turag River at Kamarpare, near Dhaka city	Padma	23.8985	90.38438	1 Dec 2014
12	Gobiidae	*Apocryptes bato*	MK572040	NRM	68033	Kullander, SO	11279	Chittagong	Maheshkhali Island, Khaler Uttarkul, Barachora Stream	Bara Matamuhu	21.5141	91.96584	11 May 2015
12	Gobiidae	*Apocryptes bato*	MK572042	NRM	66870	Kullander, SO	10158	Chittagong	Halda River at Madinagut Point	Karnafuli	22.4291	91.88801	30 Nov 2014
12	Gobiidae	*Apocryptes bato*	MK572041	NRM	66573	Kullander, SO	9782	Chittagong	Woagga Stream, 4 km W of Kaptai National Park	Karnafuli	22.5091	92.14182	28 Nov 2014
12	Gobiidae	*Apocryptes bato*	MK572039	NRM	67539	Kullander, SO	11152	Dhaka	Fish market in Shonbari, Sreenagar	Padma	23.544	90.29635	2 Dec 2014
13	Gobiidae	*Odontamblyopus rubicundus*	MK572370	NRM	67028	Kullander, SO	10283	Chittagong	Halda River at Madinagut Point	Karnafuli	22.4291	91.88801	30 Nov 2014
13	Gobiidae	*Odontamblyopus rubicundus*	MK572371	NRM	67029	Kullander, SO	10169	Chittagong	Halda River at Madinagut Point	Karnafuli	22.4291	91.88801	30 Nov 2014
14	Gobiidae	*Pseudapocryptes elongatus*	MK592410	NRM	67543	Kullander, SO	11016	Chittagong	Fishery Ghat, fish landing in Chittagong				30 Nov 2014
14	Gobiidae	*Pseudapocryptes elongatus*	MK592409	NRM	67191	Norén, M	10530	Chittagong	Lower part of small stream tributary to Naf River, about 3 km north of Teknaf	Naf	20.8986	92.27865	10 May 2015
14	Gobiidae	*Pseudapocryptes elongatus*	MK592408	NRM	67543	Kullander, SO	11015	Chittagong	Fishery Ghat, fish landing in Chittagong				30 Nov 2014
15	Gobiidae	*Parapocryptes serperaster*	MK572460	NRM	68093	Kullander, SO	11266	Chittagong	Palongkhali fish market				9 May 2015
15	Gobiidae	*Parapocryptes serperaster*	MK572459	NRM	68093	Kullander, SO	11267	Chittagong	Palongkhali fish market				9 May 2015
16	Gobiidae	*Boleophthalmus boddarti*	MK572066	NRM	67544	Kullander, SO	11020	Chittagong	Fishery Ghat, fish landing in Chittagong				30 Nov 2014
16	Gobiidae	*Boleophthalmus boddarti*	MK572067	NRM	67544	Kullander, SO	11019	Chittagong	Fishery Ghat, fish landing in Chittagong				30 Nov 2014
17	Gobiidae	*Periophthalmus barbarus*	MK572462	NRM	67026	Kullander, SO	10175	Chittagong	Halda River at Madinagut Point	Karnafuli	22.4291	91.88801	30 Nov 2014
17	Gobiidae	*Periophthalmus barbarus*	MK572461	NRM	67196	Kullander, SO	10451	Chittagong	Lower part of small stream tributary to Naf River, about 3 km north of Teknaf	Naf	20.8986	92.27865	10 May 2015
18	Gobiidae	*Awaous grammepomus*	MK572045	DU	6058	Kullander, SO	6058	Rajshahi	Batpara point, Jamuna River, Feni	Jamuna	24.1395	89.60121	2014-07-17
18	Gobiidae	*Awaous grammepomus*	MK572043	NRM	67250	Kullander, SO	10570	Chittagong	Dopachari Stream, Dudpukuria, Dopachari Wildlife Sanctuary	Sangu	22.2277	92.1256	13 May 2015
18	Gobiidae	*Awaous grammepomus*	MK572044	NRM	67934	Kullander, SO	10796	Chittagong	Mrong Bazar, small stream tributary to Sangu River, below bridge	Sangu	22.0574	92.31547	14 May 2015
19	Gobiidae	*Gobiopterus chuno*	MK572237	NRM	66876	Kullander, SO	10303	Chittagong	Halda River at Madinagut Point	Karnafuli	22.4291	91.88801	30 Nov 2014
20	Eleotridae	*Eleotris melanosoma*	MK572177	NRM	67415	Kullander, SO	10557	Chittagong	Barochora stream, 4 km south of Cox's Bazar		21.3959	92.00372	8 May 2015
20	Eleotridae	*Eleotris melanosoma*	MK572176	NRM	66835	Kullander, SO	10242	Chittagong	Bangchora hill stream in Kaptai, near hydropower station	Karnafuli	22.4958	92.18671	28 Nov 2014
20	Eleotridae	*Eleotris melanosoma*	MK572175	NRM	66837	Kullander, SO	10068	Chittagong	Bangchora hill stream in Kaptai, near hydropower station	Karnafuli	22.4958	92.18671	28 Nov 2014
20	Eleotridae	*Eleotris melanosoma*	MK572174	NRM	66839	Kullander, SO	10064	Chittagong	Bangchora hill stream in Kaptai, near hydropower station	Karnafuli	22.4958	92.18671	28 Nov 2014
20	Eleotridae	*Eleotris melanosoma*	MK572178	NRM	67545	Kullander, SO	11172	Chittagong	Halda River at Madinagut Point	Karnafuli	22.4291	91.88801	30 Nov 2014
21	Eleotridae	*Eleotris lutea*	MK572173	NRM	66836	Kullander, SO	10066	Chittagong	Bangchora hill stream in Kaptai, near hydropower station	Karnafuli	22.4958	92.18671	28 Nov 2014
22	Eleotridae	*Bunaka gyrinoides*	MK572082	NRM	67960	Kullander, SO	11262	Chittagong	Barochora stream, 4 km south of Cox's Bazar		21.3959	92.00372	8 May 2015
23	Eleotridae	*Hypseleotris cyprinoides*	MK572270	NRM	67208	Kullander, SO	10591	Chittagong	Sonarpara Bazar, Delpara stream		21.2699	92.05768	9 May 2015
24	Eleotridae	*Ophiocara porocephala*	MK572389	DU	6259	Kullander, SO	6259	Chittagong	Barachora stream				
25	Syngnathidae	*Microphis cuncalus*	MK572327	NRM	66615	Kullander, SO	9803	Dhaka	Fish market in Shonbari, Sreenagar	Padma	23.544	90.29635	2 Dec 2014
25	Syngnathidae	*Microphis cuncalus*	MK572328	NRM	66906	Kullander, SO	10028	Chittagong	Halda River at Madinagut Point	Karnafuli	22.4291	91.88801	30 Nov 2014
26	Syngnathidae	*Ichthyocampus carce*	MK572271	DU	6126	Rahman, MM	6126	Chittagong	Hatia	Meghna	21.5833	92.01666	2015-03-02
27	Tetraodontidae	*Leiodon cutcutia*	MK572287	NRM	66602	Kullander, SO	9826	Dhaka	Fish market in Shonbari, Sreenagar	Padma	23.544	90.29635	2 Dec 2014
27	Tetraodontidae	*Leiodon cutcutia*	MK572286	NRM	66942	Kullander, SO	10044	Dhaka	Local fish market in Dhaka				1 Dec 2014
27	Tetraodontidae	*Leiodon cutcutia*	MK572285	NRM	69047	Kullander, SO	11430	Dhaka	Meghna River at Ashuganj Point	Meghna	24.0463	91.00562	20 Mar 2016
28	Tetraodontidae	*Takifugu oblongus*	MK572606	DU	6127	Kullander, SO	6127	Khulna	Shamnagar, Sundarban	Sundarban	21.9748	89.19022	2014-12-14
29	Tetraodontidae	*Dichotomyctere fluviatilis*	MK572172	DU	6125	Kullander, SO	6125	Chittagong	Hatia	Meghna	21.5833	92.01666	2015-03-02

30	Poeciliidae	*Gambusia affinis*	MK572204	DU	6124	Norén, M	6124	Khulna	Shamnagar, Sundarban	Sundarban	21.9748	89.19022	2014-12-14
31	Aplocheilidae	*Aplocheilus panchax*	MK572033	NRM	67264	Kullander, SO	10504	Chittagong	Myani Stream in Dudpukuria Dopachari Wildlife Sanctuary	Sangu	22.245	92.1158	13 May 2015
31	Aplocheilidae	*Aplocheilus panchax*	MK572036	NRM	67450	Kullander, SO	10761	Chittagong	Maheshkhali Island, Khalmadia Chora, 5 km N of Maheshkhali Upazila	Bara Matamuhu	21.584	91.96534	11 May 2015
31	Aplocheilidae	*Aplocheilus panchax*	MK572034	NRM	69484	Kullander, SO	11768	Sylhet	Hail Haor, Baikka Beel, at tourist center	Meghna	24.3619	91.70741	21 Mar 2016
31	Aplocheilidae	*Aplocheilus panchax*	MK572037	NRM	66583	Kullander, SO	9838	Dhaka	Fish market in Shonbari, Sreenagar	Padma	23.544	90.29635	2 Dec 2014
31	Aplocheilidae	*Aplocheilus panchax*	MK572038	NRM	66652	Kullander, SO	9937	Chittagong	Kamila stream, tributary to Karnafuli River, 22 km south of Rangamati	Karnafuli	22.5281	92.19861	28 Nov 2014
31	Aplocheilidae	*Aplocheilus panchax*	MK572032	NRM	66650	Kullander, SO	9963	Chittagong	Kamila stream, tributary to Karnafuli River, 22 km south of Rangamati	Karnafuli	22.5281	92.19861	28 Nov 2014
31	Aplocheilidae	*Aplocheilus panchax*	MK572035	NRM	67421	Kullander, SO	10750	Chittagong	Barochora stream, 4 km south of Cox's Bazar		21.3959	92.00372	8 May 2015
32	Osphronemidae	*Trichopsis vittata*	KY327373	NRM	69396	Kullander, SO	11675	Dhaka	Deposited lands bank of Dhaleshwari River near first Dhaleswari bridge on Dhaka to Mawa hwy		23.6599	90.31086	25 Feb 2016
32	Osphronemidae	*Trichopsis vittata*	KT250365	NRM	66580	Kullander, SO	9841	Dhaka	Fish market in Shonbari, Sreenagar	Padma	23.544	90.29635	2 Dec 2014
32	Osphronemidae	*Trichopsis vittata*	KT250367	NRM	66579	Kullander, SO	9842	Dhaka	Fish market in Shonbari, Sreenagar	Padma	23.544	90.29635	2 Dec 2014
32	Osphronemidae	*Trichopsis vittata*	KY349109	NRM	69220	Kullander, SO	11587	Dhaka	Turag River	Meghna			5 Mar 2016
32	Osphronemidae	*Trichopsis vittata*	KY327374	NRM	69461	Kullander, SO	11700	Dhaka	Deposited lands bank of Dhaleshwari River near first Dhaleswari bridge on Dhaka to Mawa hwy		23.6599	90.31086	25 Feb 2016
33	Gobiidae	*Pseudogobiopsis oligactis*	MK572497	NRM	67840	Kullander, SO	10834	Chittagong	Kudung Cave in Teknaf Game Reserve		21.0923	92.16972	9 May 2015
33	Gobiidae	*Pseudogobiopsis oligactis*	MK572498	NRM	67235	Kullander, SO	10488	Chittagong	Kuikhali, small pool close to trail from Kudong Cave		21.0938	92.16811	9 May 2015
34	Heteropneustida	*Heteropneustes fossilis*	MK572261	NRM	66559	Kullander, SO	9802	Chittagong	Fishery Ghat, fish landing in Chittagong				30 Nov 2014
34	Heteropneustida	*Heteropneustes fossilis*	MK572259	NRM	66943	Kullander, SO	10050	Dhaka	Local fish market in Dhaka				1 Dec 2014
34	Heteropneustida	*Heteropneustes fossilis*	MK572260	NRM	66560	Kullander, SO	9808	Chittagong	Fishery Ghat, fish landing in Chittagong				30 Nov 2014
34	Heteropneustida	*Heteropneustes fossilis*	MK572262	NRM	68395	Kullander, SO	12197	Chittagong	Ananda Bazar Market in Brahmanbaria; fishes from Titas and Meghna River	Meghna			20 Mar 2016
35	Clariidae	*Clarias magur*	MK572128	DU	6103	Rahman, MM	6103	Dhaka	Hatirpur		23.7507	90.39568	2015-04-06
35	Clariidae	*Clarias magur*	MK572127	NRM	68892	Kullander, SO	12180	Sylhet	Hail Haor, Baikka Beel, at tourist center	Meghna	24.3619	91.70741	21 Mar 2016
35	Clariidae	*Clarias magur*	MK572129	NRM	68314	Kullander, SO	12188	Sylhet	Kushiyara River drainage, Chatul floodplain, E of Golapganj	Meghna	24.843	92.05717	23 Mar 2016
36	Bagridae	*Pachypterus atherinoides*	MK572421	DU	6245	Rahman, MM	6245	Dhaka	Mohammadpur Bazar		23.7507	90.39568	2015-02-22
36	Horabagridae	*Pachypterus atherinoides*	MK572419	NRM	66548	Kullander, SO	10203	Chittagong	Rangamati, town fish market, fish principally from Kaptai Lake	Karnafuli	22.6497	92.18372	27 Nov 2014
36	Horabagridae	*Pachypterus atherinoides*	MK572418	NRM	66549	Kullander, SO	10204	Chittagong	Rangamati, town fish market, fish principally from Kaptai Lake	Karnafuli	22.6497	92.18372	27 Nov 2014
36	Horabagridae	*Pachypterus atherinoides*	MK572420	NRM	66530	Kullander, SO	9815	Dhaka	Market at Ashulia Point, Turag River	Padma			1 Dec 2014
36	Horabagridae	*Pachypterus atherinoides*	MK572417	NRM	66972	Kullander, SO	10351	Dhaka	Padma River near Srinagar	Padma			2 Dec 2014
36	Horabagridae	*Pachypterus atherinoides*	MK572422	NRM	68973	Kullander, SO	11342	Sylhet	Piyain Goyan River near Goyanghat	Meghna	25.0883	91.98152	25 Mar 2016
37	Bagridae	*Hemibagrus menoda*	MK572257	DU	6134	Rahman, MM	6134	Mymenshing	Itna Haor	Jamuna	24.5309	91.10439	2014-10-02
37	Bagridae	*Hemibagrus menoda*	MK572258	NRM	68356	Kullander, SO	12185	Dhaka	Bhairab Fish Market, fish from Meghna River	Meghna	24.05	90.98333	20 Mar 2016
38	Bagridae	*Mystus tengara*	MK572353	DU	6158	Rahman, MM	6158	Dhaka	Buriganga River	Padma	23.7291	90.35398	2014-09-13
38	Bagridae	*Mystus tengara*	MK572351	NRM	67461	Kullander, SO	11104	Chittagong	Rangamati, fish landing pier, fish from Kaptai Lake	Karnafuli	22.6501	92.18571	27-28 Nov 2014
38	Bagridae	*Mystus tengara*	MK572352	NRM	66655	Kullander, SO	9943	Chittagong	Kamila stream, tributary to Karnafuli River, 22 km south of Rangamati	Karnafuli	22.5281	92.19861	28 Nov 2014
38	Bagridae	*Mystus tengara*	MK572354	NRM	66703	Kullander, SO	9889	Chittagong	Roadside ditch in Sultanpur	Karnafuli	22.5422	91.8997	29 Nov 2014
39	Bagridae	*Mystus carcio*	MK572340	DU	6239	Rahman, MM	6239	Mymenshing	Sherpur market	Jamuna	25.0229	90.01728	2015-03-21
39	Bagridae	*Mystus carcio*	MK572337	NRM	66954	Kullander, SO	10012	Dhaka	Local fish market in Dhaka				1 Dec 2014
39	Bagridae	*Mystus carcio*	MK572338	NRM	66957	Kullander, SO	10015	Dhaka	Local fish market in Dhaka				1 Dec 2014
39	Bagridae	*Mystus carcio*	MK572336	NRM	66951	Kullander, SO	10009	Dhaka	Local fish market in Dhaka				1 Dec 2014
40	Bagridae	*Mystus bleekeri*	MK572335	NRM	68977	Kullander, SO	11349	Sylhet	Piyain Goyan River near Goyanghat	Meghna	25.0883	91.98152	25 Mar 2016
40	Bagridae	*Mystus bleekeri*	MK572334	NRM	67462	Kullander, SO	11155	Dhaka	Fish market in Shonbari, Sreenagar	Padma	23.544	90.29635	2 Dec 2014
41	Bagridae	*Mystus gulio*	MK572345	DU	9013	Rahman. MM	9013	Chittagong	Keruntoli	Naf			2014-10-31
41	Bagridae	*Mystus gulio*	MK572346	DU	9016	Rahman. MM	9016	Chittagong	Nidania	Naf			2014-10-30
41	Bagridae	*Mystus gulio*	MK572350	DU	9010	Rahman, MM	9010	Chittagong	Keruntoli or Naitong Para	Naf			2014-10-31
41	Bagridae	*Mystus gulio*	MK572349	NRM	67577	Kullander, SO	11021	Chittagong	Fishery Ghat, fish landing in Chittagong				30 Nov 2014
41	Bagridae	*Mystus gulio*	MK572348	NRM	67577	Kullander, SO	11022	Chittagong	Fishery Ghat, fish landing in Chittagong				30 Nov 2014
41	Bagridae	*Mystus gulio*	MK572347	NRM	67961	Kullander, SO	11285	Chittagong	Rangamati, fish landing pier, fish from Kaptai Lake	Karnafuli	22.6501	92.18571	27-28 Nov 2014
42	Bagridae	*Mystus cavasius*	MK572342	NRM	68916	Kullander, SO	12211	Sylhet	Piyain Goyan River near Goyanghat	Meghna	25.0883	91.98152	25 Mar 2016
42	Bagridae	*Mystus cavasius*	MK572344	NRM	67459	Kullander, SO	10999	Chittagong	Rangamati, fish landing pier, fish from Kaptai Lake	Karnafuli	22.6501	92.18571	27-28 Nov 2014
42	Bagridae	*Mystus cavasius*	MK572343	NRM	67459	Kullander, SO	11000	Chittagong	Rangamati, fish landing pier, fish from Kaptai Lake	Karnafuli	22.6501	92.18571	27-28 Nov 2014
43	Bagridae	*Sperata aorella*	MK572600	NRM	67298	Kullander, SO	11002	Chittagong	Rangamati, fish landing pier, fish from Kaptai Lake	Karnafuli	22.6501	92.18571	27-28 Nov 2014
43	Bagridae	*Sperata aorella*	MK572602	NRM	67298	Kullander, SO	11003	Chittagong	Rangamati, fish landing pier, fish from Kaptai Lake	Karnafuli	22.6501	92.18571	27-28 Nov 2014
43	Bagridae	*Sperata aorella*	MK572601	NRM	69231	Kullander, SO	11517	Sylhet	Rostumpur, roadside ditch near Goyanghat	Meghna	25.0992	91.92037	25 Mar 2016
44	Bagridae	*Sperata seenghala*	MK572603	NRM	68886	Kullander, SO	12195	Sylhet	Surma River left bank, at Kheaghat point, 1,5 km upstream from Golapganj	Meghna	24.8612	91.99371	23 Mar 2016
45	Bagridae	*Sperata aor*	MK572599	NRM	67457	Kullander, SO	11006	Chittagong	Rangamati, fish landing pier, fish from Kaptai Lake	Karnafuli	22.6501	92.18571	27-28 Nov 2014
45	Bagridae	*Sperata aor*	MK572598	NRM	67457	Kullander, SO	11005	Chittagong	Rangamati, fish landing pier, fish from Kaptai Lake	Karnafuli	22.6501	92.18571	27-28 Nov 2014
45	Bagridae	*Sperata aor*	MK572597	NRM	68355	Kullander, SO	12183	Dhaka	Bhairab Fish Market, fish from Meghna River	Meghna	24.05	90.98333	20 Mar 2016
46	Ailiidae	*Clupisoma garua*	MK572133	NRM	66937	Kullander, SO	10048	Dhaka	Local fish market in Dhaka				1 Dec 2014
46	Ailiidae	*Clupisoma garua*	MK572130	NRM	68887	Kullander, SO	12190	Sylhet	Surma River left bank, at Kheaghat point, 1,5 km upstream from Golapganj	Meghna	24.8612	91.99371	23 Mar 2016
46	Ailiidae	*Clupisoma garua*	MK572132	NRM	70149	Kullander, SO	12570	Chittagong	Ananda Bazar Market in Brahmanbaria; fishes from Titas and Meghna River	Meghna			20 Mar 2016
46	Ailiidae	*Clupisoma garua*	MK572135	NRM	70147	Kullander, SO	12568	Chittagong	Ananda Bazar Market in Brahmanbaria; fishes from Titas and Meghna River	Meghna			20 Mar 2016
46	Ailiidae	*Clupisoma garua*	MK572134	NRM	66936	Kullander, SO	10042	Dhaka	Local fish market in Dhaka				1 Dec 2014
46	Ailiidae	*Clupisoma garua*	MK572131	NRM	67576	Kullander, SO	11007	Chittagong	Rangamati, fish landing pier, fish from Kaptai Lake	Karnafuli	22.6501	92.18571	27-28 Nov 2014
47	Ailiidae	*Eutropiichthys vacha*	MK572194	NRM	70161	Kullander, SO	12582	Chittagong	Ananda Bazar Market in Brahmanbaria; fishes from Titas and Meghna River	Meghna			20 Mar 2016
47	Ailiidae	*Eutropiichthys vacha*	MK572193	NRM	67578	Kullander, SO	11008	Chittagong	Rangamati, fish landing pier, fish from Kaptai Lake	Karnafuli	22.6501	92.18571	27-28 Nov 2014
47	Ailiidae	*Eutropiichthys vacha*	MK572196	NRM	68882	Kullander, SO	12187	Sylhet	Surma River left bank, at Kheaghat point, 1,5 km upstream from Golapganj	Meghna	24.8612	91.99371	23 Mar 2016
47	Ailiidae	*Eutropiichthys vacha*	MK5722195	NRM	67580	Kullander, SO	11063	Dhaka	Market at Ashulia Point, Turag River	Padma			1 Dec 2014
48	Ailiidae	*Eutropiichthys murius*	MK572192	DU	6055	Rahman, MM	6055	Rajshahi	Baghmara point , Shjadpur, Shirajgonj	Jamuna	24.1395	89.60121	2014-07-17
49	Ailiidae	*Silonia silondia*	MK572596	NRM	67469	Kullander, SO	11146	Dhaka	Fish market in Shonbari, Sreenagar	Padma	23.544	90.29635	2 Dec 2014
49	Ailiidae	*Silonia silondia*	MK572595	NRM	67469	Kullander, SO	11143	Dhaka	Fish market in Shonbari, Sreenagar	Padma	23.544	90.29635	2 Dec 2014
50	Ailiidae	*Ailia coila*	MK572008	NRM	67476	Kullander, SO	11129	Chittagong	Rangamati, fish landing pier, fish from Kaptai Lake	Karnafuli	22.6501	92.18571	27-28 Nov 2014
50	Ailiidae	*Ailia coila*	MK572010	NRM	70170	Kullander, SO	12591	Chittagong	Ananda Bazar Market in Brahmanbaria; fishes from Titas and Meghna River	Meghna			20 Mar 2016

50	Ailiidae	*Ailia coila*	MK572009	NRM	70169	Kullander, SO	12590	Chittagong	Ananda Bazar Market in Brahmanbaria; fishes from Titas and Meghna River	Meghna			20 Mar 2016
51	Pangasiidae	*Pangasius pangasius*	MK572424	NRM	67575	Kullander, SO	11049	Dhaka	Local fish market in Dhaka				1 Dec 2014
52	Pangasiidae	*Pangasianodon hypophthalmus*	MK572423	DU	6100	Rahman, MM	6100	Dhaka	Kaoran Bazar		23.7507	90.39568	2015-01-30
53	Sisoridae	*Glyptothorax telchitta*	MK572233	DU	6150	Kullander, SO	6150	Dhaka	Buriganga River	Padma	23.7291	90.35398	2014-09-13
53	Sisoridae	*Glyptothorax telchitta*	MK572234	NRM	66612	Kullander, SO	9857	Dhaka	Fish market in Shonbari, Sreenagar	Padma	23.544	90.29635	2 Dec 2014
53	Sisoridae	*Glyptothorax telchitta*	MK572232	NRM	66609	Kullander, SO	9824	Dhaka	Fish market in Shonbari, Sreenagar	Padma	23.544	90.29635	2 Dec 2014
53	Sisoridae	*Glyptothorax telchitta*	MK572235	NRM	68226	Kullander, SO	11344	Chittagong	Kokkhiyon Jhiri stream, 11 km W of Ruma	Sangu	22.058	92.34182	14 May 2015
53	Sisoridae	*Glyptothorax telchitta*	MK572236	NRM	66040	Kullander, SO	11842	Sylhet	Piyain River at Jaflong	Meghna	25.1844	92.01642	24 Mar 2016
54	Sisoridae	*Glyptothorax botius*	MK572231	NRM	66605	Kullander, SO	9855	Dhaka	Fish market in Shonbari, Sreenagar	Padma	23.544	90.29635	2 Dec 2014
55	Sisoridae	*Glyptothorax* cf *trilineata*	MK572230	DU	6283	Kullander, SO	6283	Barisal	Barishal	Noakhali			
55	Sisoridae	*Glyptothorax* cf *trilineata*	MK572229	NRM	65568	Kullander, SO	9873	Barisal	Barishal	Noakhali			Unknown date
56	Sisoridae	*Bagarius bagarius*	MK572046	NRM	66618	Kullander, SO	9846	Dhaka	Fish market in Shonbari, Sreenagar	Padma	23.544	90.29635	2 Dec 2014
57	Sisoridae	*Pseudolaguvia* sp3	MK572502	NRM	67419	Kullander, SO	10611	Chittagong	Barochora stream, 4 km south of Cox's Bazar	Matamuhuri	21.3959	92.00372	8 May 2015
57	Sisoridae	*Pseudolaguvia* sp3	MK572501	NRM	67420	Kullander, SO	10612	Chittagong	Barochora stream, 4 km south of Cox's Bazar	Matamuhuri	21.3959	92.00372	8 May 2015
58	Sisoridae	*Pseudolaguvia* sp2	MK572504	DU	6220	Kullander, SO	6220	Mymenshing	Chilakhali stream	Jamuna	25.2543	90.03265	21 mar-2015
58	Sisoridae	Pseudolaguvia ribeiroi Sherpur	MK572507	DU	6269	Kullander, SO	DU6269	Dinajpur	Atrai River	Padma			3 maj-2014
59	Sisoridae	*Pseudolaguvia* sp4	MK572500	NRM	67933	Kullander, SO	10867	Chittagong	Udalbania, stream at entrance to Dudpukuria-Dhopachori Wildlife Sanctuary, below bridge	Karnafuli	22.3105	92.15218	12 May 2015
60	Sisoridae	*Pseudolaguvia* sp1	MK572499	NRM	69613	Kullander, SO	12123	Sylhet	River at Bisnakandi, coming from Meghalaya, tributary to Surma River, riffles	Meghna	25.1753	91.88661	25 Mar 2016
61	Sisoridae	*Pseudolaguvia shawi*	MK572508	DU	6207	Ng Heok Hee	6207	Mymenshing	Chilakhali stream	Jamuna	25.2543	90.03265	2015-03-21
62	Sisoridae	*Pseudolaguvia sp*	MK572505	NRM	69609	Kullander, SO	12145	Sylhet	Piyain River at Jaflong	Meghna	25.1844	92.01642	24 Mar 2016
62	Sisoridae	*Pseudolaguvia fucosa*	MK572506	NRM	68229	Kullander, SO	11317	Chittagong	Kokkhiyon Jhiri stream, 11 km W of Ruma	Sangu	22.058	92.34182	14 May 2015
62	Sisoridae	*Pseudolaguvia sp*	MK572503	NRM	69612	Kullander, SO	12128	Sylhet	River at Bisnakandi, coming from Meghalaya, tributary to Surma River, riffles	Meghna	25.1753	91.88661	25 Mar 2016
64	Sisoridae	*Erethistes jerdoni*	MK572179	NRM	69381	Kullander, SO	11660	Sylhet	Piyain River at Jaflong	Meghna	25.1844	92.01642	24 Mar 2016
65	Sisoridae	*Erethistes pusillus*	MK572180	NRM	69382	Kullander, SO	11661	Sylhet	Kushiyara River left bank in Fenchuganj at junction with Juri River	Meghna	24.7054	91.95432	22 Mar 2016
65	Sisoridae	*Erethistes pusillus*	MK572181	NRM	69390	Kullander, SO	11669	Chittagong	Salda River where crossed by Brahmanbaria-Comilla Bypass Road	Meghna	23.6693	91.14957	19 Mar 2016
65	Sisoridae	*Erethistes pusillus*	MK572183	NRM	69394	Kullander, SO	11673	Sylhet	Surma River left bank, at Kheaghat point, 1,5 km upstream from Golapganj	Meghna	24.8612	91.99371	23 Mar 2016
65	Sisoridae	*Erethistes pusillus*	MK572182	DU	6154	Kullander, SO	6154	Dhaka	Buriganga River	Padma	23.7291	90.35398	2014-09-13
66	Sisoridae	*Erethistoides sp*	MK572185	NRM	69608	Kullander, SO	12126	Sylhet	Piyain River at Jaflong	Meghna	25.1844	92.01642	24 Mar 2016
66	Sisoridae	*Erethistoides sp*	MK572184	NRM	69607	Kullander, SO	12127	Sylhet	Piyain River at Jaflong near Jaflong Bazar	Meghna	25.1543	92.02039	24 Mar 2016
67	Sisoridae	*Nangra assamensis*	MK572355	NRM	66611	Kullander, SO	9854	Dhaka	Fish market in Shonbari, Sreenagar	Padma	23.544	90.29635	2 Dec 2014
68	Sisoridae	*Nangra bucculenta*	MK572356	NRM	65769	Kullander, SO	12099	Sylhet	Kushiyara River left bank in Fenchuganj at junction with Juri River	Meghna	24.7054	91.95432	22 Mar 2016
69	Sisoridae	*Gagata cenia*	MK572199	NRM	69285	Kullander, SO	11480	Sylhet	Kushiyara River left bank in Fenchuganj at junction with Juri River	Meghna	24.7054	91.95432	22 Mar 2016
69	Sisoridae	*Gagata cenia*	MK572198	NRM	66550	Kullander, SO	10177	Chittagong	Rangamati, town fish market, fish principally from Kaptai Lake	Karnafuli	22.6497	92.18372	27 Nov 2014
69	Sisoridae	*Gagata cenia*	MK572197	NRM	67000	Kullander, SO	10054	Dhaka	Padma River near Srinagar	Padma			2 Dec 2014
69	Sisoridae	*Gagata cenia*	MK572200	DU	6160	Kullander, SO	6160	Dhaka	Buriganga River	Padma	23.7291	90.35398	2014-09-13
70	Sisoridae	*Gagata gagata*	MK572201	NRM	67176	Kullander, SO	10552	Chittagong	Sangu River at Dhopachari	Sangu	22.2061	92.13399	13 May 2015
70	Sisoridae	*Gagata gagata*	MK572202	NRM	67574	Kullander, SO	11169	Chittagong	Halda River at Madinagut Point	Karnafuli	22.4291	91.88801	30 Nov 2014
70	Sisoridae	*Gagata gagata*	MK572203	DU	6091	Kullander, SO	6091	Dhaka	Mohammadpur Bazar		23.7507	90.39568	2015-02-22
71	Sisoridae	*Gogangra laevis*	MK572242	NRM	66613	Kullander, SO	9860	Dhaka	Fish market in Shonbari, Sreenagar	Padma	23.544	90.29635	2 Dec 2014
71	Sisoridae	*Gogangra laevis*	MK572240	NRM	65770	Kullander, SO	12100	Sylhet	Kushiyara River left bank in Fenchuganj at junction with Juri River	Meghna	24.7054	91.95432	22 Mar 2016
71	Sisoridae	*Gogangra laevis*	MK572238	NRM	69350	Kullander, SO	11575	Dhaka	Meghna River at Ashuganj Point	Meghna	24.0463	91.00562	20 Mar 2016
71	Sisoridae	*Gogangra laevis*	MK572239	NRM	69371	Kullander, SO	11576	Sylhet	Kushiyara River left bank in Fenchuganj at junction with Juri River	Meghna	24.7054	91.95432	22 Mar 2016
71	Sisoridae	*Gogangra laevis*	MK572241	DU	6153	Kullander, SO	6153	Dhaka	Buriganga River	Padma	23.7291	90.35398	2014-09-13
72	Amblycipitidae	*Amblyceps mangois*	MK572015	DU	6242	Kullander, SO	6242	Chittagong	Taichala Stream	Karnafuli	23.1038	91.96689	2015-04-26
72	Amblycipitidae	*Amblyceps mangois*	MK572016	NRM	66788	Kullander, SO	10289	Chittagong	Stream at southeastern margin of Chittagong University campus	Karnafuli	22.4603	91.7967	29 Nov 2014
72	Amblycipitidae	*Amblyceps mangois*	MK572014	NRM	69716	Kullander, SO	12268	Chittagong	Shaila Propat hillstream	Sangu			13 Feb 2016
72	Amblycipitidae	*Amblyceps mangois*	MK572011	NRM	67687	Kullander, SO	10679	Chittagong	Small stream tributary to Sangu River,under bridge near Munlai Para village, east of Ruma	Sangu	22.0328	92.41753	14 May 2015
72	Amblycipitidae	*Amblyceps mangois*	MK572013	NRM	66706	Kullander, SO	10127	Sylhet	Pyiang River at Jaflong, about 60 km from Sylhet city	Meghna	25.1862	92.01667	25 Apr 2014
72	Amblycipitidae	*Amblyceps mangois*	MK572012	DU	6264	Kullander, SO	6264	Sylhet	Piyang River, Jaflong, Sylhet	Meghna			2014-04-25
73	Siluridae	*Ompok bimaculatus*	MK572383	NRM	67467	Kullander, SO	11144	Dhaka	Fish market in Shonbari, Sreenagar	Padma	23.544	90.29635	2 Dec 2014
73	Siluridae	*Ompok bimaculatus*	MK572384	NRM	67470	Kullander, SO	11087	Chittagong	Rangamati, town fish market, fish principally from Kaptai Lake	Karnafuli	22.6497	92.18372	27 Nov 2014
74	Siluridae	*Ompok pabo*	MK572387	NRM	68913	Kullander, SO	12214	Sylhet	Piyain Goyan River near Goyanghat	Meghna	25.0883	91.98152	25 Mar 2016
74	Siluridae	*Ompok pabo*	MK572388	NRM	67471	Kullander, SO	12655	Chittagong	Rangamati, fish landing pier, fish from Kaptai Lake	Karnafuli	22.6501	92.18571	27-28 Nov 2014
75	Siluridae	*Ompok pabda*	MK572385	NRM	67581	Kullander, SO	11145	Dhaka	Fish market in Shonbari, Sreenagar	Padma	23.544	90.29635	2 Dec 2014
75	Siluridae	*Ompok pabda*	MK572386	NRM	69321	Kullander, SO	11639	Sylhet	Hail Haor, Baikka Beel, at tourist center	Meghna	24.3619	91.70741	21 Mar 2016
76	Siluridae	*Pterocryptis barakensis*	MK572524	NRM	67334	Kullander, SO	11263	Chittagong	Kudung Cave in Teknaf Game Reserve		21.0923	92.16972	9 May 2015
76	Siluridae	*Pterocryptis barakensis*	MK572523	NRM	67334	Kullander, SO	11264	Chittagong	Kudung Cave in Teknaf Game Reserve		21.0923	92.16972	9 May 2015
77	Siluridae	*Wallago attu*	MK572628	NRM	68365	Kullander, SO	12209	Sylhet	Hakaluki Haor in Borolek	Meghna	24.6617	92.03478	26 Mar 2016
78	Ritaidae	*Rita rita*	MK572552	NRM	66599	Kullander, SO	9856	Dhaka	Fish market in Shonbari, Sreenagar	Padma	23.544	90.29635	2 Dec 2014
78	Ritaidae	*Rita rita*	MK572551	DU	6036	Kullander, SO	6036	Barisal	Barishal Fish Landing	Meghna	22.7396	90.43307	2014-11-01
79	Bagridae	*Batasio batasio*	MK572063	NRM	69228	Kullander, SO	11504	Sylhet	Piyain Goyan River near Goyanghat	Meghna	25.0883	91.98152	25 Mar 2016
79	Bagridae	*Batasio batasio*	MK572065	NRM	69271	Kullander, SO	11511	Sylhet	Surma River left bank, at Kheaghat point, 1,5 km upstream from Golapganj	Meghna	24.8612	91.99371	23 Mar 2016
79	Bagridae	*Batasio batasio*	MK572064	NRM	69272	Kullander, SO	11512	Sylhet	Surma River left bank, at Kheaghat point, 1,5 km upstream from Golapganj	Meghna	24.8612	91.99371	23 Mar 2016
80	Bagridae	*Rama chandramara*	MK572104	NRM	69451	Kullander, SO	11690	Dhaka	Bhairab Fish Market, fish from Meghna	Meghna	24.05	90.98333	20 Mar 2016
80	Bagridae	*Rama chandramara*	MK572102	NRM	69695	Kullander, SO	12229	Dhaka	Bhairab Fish Market, fish from Meghna River	Meghna	24.05	90.98333	20 Mar 2016
80	Bagridae	*Rama chandramara*	MK572103	NRM	69694	Kullander, SO	12228	Dhaka	Bhairab Fish Market, fish from Meghna River	Meghna	24.05	90.98333	20 Mar 2016
80	Bagridae	*Rama chandramara*	MK572101	NRM	69450	Kullander, SO	11689	Dhaka	Bhairab Fish Market, fish from Meghna River	Meghna	24.05	90.98333	20 Mar 2016
81	Chacidae	*Chaca chaca*	MK572091	DU	6266	Kullander, SO	DU6266		Bangladesh, no further data saved				
82	Chacidae	*Chaca chaca* Meghna	MK572090	NRM	68391	Kullander, SO	12533	Sylhet	Fish Market in Golapgonj	Meghna			23 Mar 2016
83	Bagridae	*Olyra* sp	MK572379	NRM	67396	Kullander, SO	10735	Chittagong	Barochora stream, 4 km south of Cox's Bazar	Mutamuhuri	21.3959	92.00372	8 May 2015
83	Bagridae	*Olyra* sp	MK572375	NRM	67389	Kullander, SO	10754	Chittagong	Barochora stream, 4 km south of Cox's Bazar	Mutamuhuri	21.3959	92.00372	8 May 2015

83	Bagridae	*Olyra* sp	MK572378	NRM	67364	Kullander, SO	10588	Chittagong	Godarpar Stream at Sonarpara Bazar, small stream to Bay of Bengal	Mutamuhuri	21.279	92.0566	9 May 2015
83	Bagridae	*Olyra* sp	MK572377	DU	6238	Kullander, SO	6238	Chittagong	Taichala Stream	Karnafuli	23.1038	91.96689	2015-04-26
83	Bagridae	*Olyra* sp	MK572374	DU	6164	Kullander, SO	6164	Chittagong	Taichala Stream	Karnafuli	23.1038	91.96689	2015-04-26
83	Bagridae	*Olyra* sp	MK572376	DU	6164	Kullander, SO	6164	Chittagong	Taichala Stream	Karnafuli	23.1038	91.96689	2015-04-26
84	Bagridae	*Olyra longicaudata*	MK572382	NRM	66039	Kullander, SO	11837	Sylhet	Piyain River at Jaflong	Meghna	25.1844	92.01642	24 Mar 2016
84	Bagridae	*Olyra longicaudata*	MK572381	NRM	65775	Kullander, SO	12104	Sylhet	Piyain River at Jaflong	Meghna	25.1844	92.01642	24 Mar 2016
84	Bagridae	*Olyra longicaudata*	MK572380	NRM	66193	Kullander, SO	12118	Sylhet	River at Bisnakandi, coming from Meghalaya, tributary to Meghna River, sandy bed stream	Meghna	25.1592	91.89299	25 Mar 2016
85	Bagridae	*Olyra* sp1	MK572373	NRM	67767	Kullander, SO	10991	Chittagong	Udalbania, small stream left bank tributary to Karnafuli River, flowing through tobacco plant	Karnafuli	22.3245	92.139	12 May 2015
85	Bagridae	*Olyra* sp1	MK572372	NRM	67768	Kullander, SO	10985	Chittagong	Udalbania, small stream left bank tributary to Karnafuli River, flowing through tobacco plant	Karnafuli	22.3245	92.139	12 May 2015
86	Loricariidae	*Pterygoplichthys* sp	MK572526	DU	6095	Rahman, MM	6095	Dhaka	Noyabazar		23.7507	90.39568	2015-04-07
86	Loricariidae	*Pterygoplichthys* sp	MK572525	DU	6093	Rahman, MM	6093	Dhaka	Noyabazar		23.7507	90.39568	2015-04-07
86	Loricariidae	*Pterygoplichthys* sp	MK572527	DU	6094	Rahman, MM	6094	Dhaka	Noyabazar		23.7507	90.39568	2015-04-07
87	Channidae	*Channa punctata*	MK572114	NRM	66577	Kullander, SO	9844	Dhaka	Fish market in Shonbari, Sreenagar	Padma	23.544	90.29635	2 Dec 2014
87	Channidae	*Channa punctata*	MK572117	NRM	67236	Kullander, SO	10469	Chittagong	Kuikhali, small pool close to trail from Kudong Cave		21.0938	92.16811	9 May 2015
87	Channidae	*Channa punctata*	MK572118	NRM	67709	Kullander, SO	11117	Chittagong	Rangamati, fish landing pier, fish from Kaptai Lake	Karnafuli	22.6501	92.18571	27-28 Nov 2014
88	Channidae	*Channa punctata*	MK572115	NRM	69602	Kullander, SO	12085	Chittagong	Titas River in Akhaura	Meghna	23.8843	91.20121	19 Mar 2016
88	Channidae	*Channa punctata*	MK572116	NRM	67958	Kullander, SO	11269	Chittagong	Teknaf Wildlife Park, small stream	Naf	20.932	92.25827	10 May 2015
88	Channidae	*Channa gachua*	MK572111	NRM	66603	Kullander, SO	9850	Dhaka	Fish market in Shonbari, Sreenagar	Padma	23.544	90.29635	2 Dec 2014
89	Channidae	*Channa gachua*	MK572106	NRM	69351	Kullander, SO	11565	Dhaka	Meghna River at Ashuganj Point	Meghna	24.0463	91.00562	20 Mar 2016
90	Channidae	*Channa* sp cf *gachua*	MK572105	NRM	67312	Kullander, SO	10578	Chittagong	Shaila Propat Waterfall and downstream pools	Sangu	22.1515	92.2163	14 May 2015
91	Channidae	*Channa gachua*	MK572110	NRM	67452	Kullander, SO	10768	Chittagong	Maheshkhali Island, Khalmadia Chora, 5 km N of Maheshkhali Upazila	Bara Matamuhu	21.584	91.96534	11 May 2015
91	Channidae	*Channa gachua*	MK572108	NRM	68120	Kullander, SO	11278	Chittagong	Maheshkhali Island, small stream in Khalmadia Chora, 8 km north of Maheshkhali Upazila	Bara Matamuhu	21.5873	91.96645	11 May 2015
91	Channidae	*Channa gachua*	MK572109	NRM	67940	Kullander, SO	10806	Chittagong	Mrong Bazar, small stream tributary to Sangu River, below bridge	Sangu	22.0574	92.31547	14 May 2015
91	Channidae	*Channa gachua*	MK572107	NRM	67956	Kullander, SO	11288	Chittagong	Small stream about 2 km north of Teknaf on Cox's Bazar-Teknaf Highway	Naf	20.8821	92.29758	10 May 2015
92	Channidae	*Channa marulius*	MK572113	NRM	67708	Kullander, SO	11120	Chittagong	Rangamati, fish landing pier, fish from Kaptai Lake	Karnafuli	22.6501	92.18571	27-28 Nov 2014
92	Channidae	*Channa marulius*	MK572112	NRM	67708	Kullander, SO	11119	Chittagong	Rangamati, fish landing pier, fish from Kaptai Lake	Karnafuli	22.6501	92.18571	27-28 Nov 2014
93	Channidae	*Channa striata*	MK572121	NRM	68137	Kullander, SO	11265	Chittagong	Godarpar Stream at Sonarpara Bazar, small stream to Bay of Bengal		21.279	92.0566	9 May 2015
93	Channidae	*Channa striata*	MK572119	NRM	67365	Kullander, SO	10584	Chittagong	Godarpar Stream at Sonarpara Bazar, small stream to Bay of Bengal		21.279	92.0566	9 May 2015
94	Channidae	*Channa striata*	MK572122	NRM	67710	Kullander, SO	11114	Chittagong	Rangamati, fish landing pier, fish from Kaptai Lake	Karnafuli	22.6501	92.18571	27-28 Nov 2014
94	Channidae	*Channa striata*	MK572120	NRM	67710	Kullander, SO	11115	Chittagong	Rangamati, fish landing pier, fish from Kaptai Lake	Karnafuli	22.6501	92.18571	27-28 Nov 2014
95	Nandidae	*Nandus nandus*	MK029410	NRM	66608	Kullander, SO	9825	Dhaka	Fish market in Shonbari, Sreenagar	Padma	23.544	90.29635	2 Dec 2014
95	Nandidae	*Nandus nandus*	MK029411	NRM	68968	Kullander, SO	11455	Sylhet	Kushiyara River drainage, Chatul floodplain, E of Golapganj	Meghna	24.843	92.05717	23 Mar 2016
96	Cynoglossidae	*Cynoglossus cynoglossus*	MK572143	NRM	66877	Kullander, SO	10166	Chittagong	Halda River at Madinagut Point	Karnafuli	22.4291	91.88801	30 Nov 2014
96	Cynoglossidae	*Cynoglossus cynoglossus*	MK572144	NRM	67024	Kullander, SO	10174	Chittagong	Halda River at Madinagut Point	Karnafuli	22.4291	91.88801	30 Nov 2014
97	Polynemidae	*Polynemus paradiseus*	MK572496	NRM	66859	Kullander, SO	10192	Chittagong	Halda River at Madinagut Point	Karnafuli	22.4291	91.88801	30 Nov 2014
97	Polynemidae	*Polynemus paradiseus*	MK572495	NRM	67478	Kullander, SO	11039	Chittagong	Fishery Ghat, fish landing in Chittagong				30 Nov 2014
98	Latidae	*Lates calcarifer*	MK572284	NRM	67596	Kullander, SO	11032	Chittagong	Fishery Ghat, fish landing in Chittagong				30 Nov 2014
99	Soleidae	*Brachirus orientalis*	MK572076	NRM	66875	Kullander, SO	10344	Chittagong	Halda River at Madinagut Point	Karnafuli	22.4291	91.88801	30 Nov 2014
99	Soleidae	*Brachirus orientalis*	MK572075	NRM	66874	Kullander, SO	10307	Chittagong	Halda River at Madinagut Point	Karnafuli	22.4291	91.88801	30 Nov 2014
100	Badidae	*Badis badis*	MK029393	NRM	69025	Kullander, SO	11528	Sylhet	Kushiyara River drainage, Chatul floodplain, E of Golapganj	Meghna	24.843	92.05717	23 Mar 2016
100	Badidae	*Badis badis*	MK029383	NRM	66682	Kullander, SO	9925	Dhaka	Turag River at Kamarpare, near Dhaka city	Padma	23.8985	90.38438	1 Dec 2014
100	Badidae	*Badis badis*	MK029396	NRM	66683	Kullander, SO	9926	Dhaka	Turag River at Kamarpare, near Dhaka city	Padma	23.8985	90.38438	1 Dec 2014
100	Badidae	*Badis badis*	MK029388	NRM	69449	Kullander, SO	11688	Chittagong	Titas River in Akhaura	Meghna	23.8843	91.20121	19 Mar 2016
100	Badidae	*Badis badis*	MK567705	NRM	66576	Kullander, SO	9845	Dhaka	Fish market in Shonbari, Sreenagar	Padma	23.544	90.29635	2 Dec 2014
100	Badidae	*Badis badis*	MK029394	NRM	69042	Kullander, SO	11436	Dhaka	Meghna River at Ashuganj Point	Meghna	24.0463	91.00562	20 Mar 2016
100	Badidae	*Badis badis*	MK029386	NRM	69478	Kullander, SO	11774	Sylhet	Hail Haor, Baikka Beel, at tourist center	Meghna	24.3619	91.70741	21 Mar 2016
100	Badidae	*Badis badis*	MK029395	NRM	68820	Kullander, SO	11410	Sylhet	Hakaluki Haor in Borolek	Meghna	24.6617	92.03478	26 Mar 2016
100	Badidae	*Badis badis*	MK029387	NRM	69455	Kullander, SO	11694	Dhaka	Deposited lands bank of Dhaleshwari River near first Dhaleswari bridge on Dhaka to Mawa	Daleshwari	23.6599	90.31086	25 Feb 2016
100	Badidae	*Badis badis*	MK029389	NRM	69406	Kullander, SO	11679	Dhaka	Kaligonj, narrow man-made canal running across a rice-field floodplain, to Turag River	Meghna	23.9872	90.52856	4 Mar 2016
100	Badidae	*Badis badis*	MK029392	DU	6247	Kullander, SO	6247	Mymenshing	Old Bramaputra, BAU	Jamuna	24.7315	92.43496	2015-03-21
101	Badidae	*Badis pallidus*	MK029376	NRM	66773	Kullander, SO	10388	Chittagong	Roadside ditch in Sultanpur	Karnafuli	22.5422	91.8997	29 Nov 2014
101	Badidae	*Badis pallidus*	MK029377	NRM	66790	Kullander, SO	10238	Chittagong	Stream at southeastern margin of Chittagong University campus	Karnafuli	22.4603	91.7967	29 Nov 2014
101	Badidae	*Badis pallidus*	MK029406	NRM	66816	Kullander, SO	10296	Chittagong	Hillstream at northwestern margin of Chittagong University campus, in and below rapids	Karnafuli	22.4738	91.78312	29 Nov 2014
101	Badidae	*Badis pallidus*	MK029382	NRM	67950	Kullander, SO	10820	Chittagong	Mrong Bazar, small stream tributary to Sangu River, below bridge	Sangu	22.0574	92.31547	14 May 2015
101	Badidae	*Badis pallidus*	MK029379	NRM	67273	Kullander, SO	10565	Chittagong	Myani Stream in Dudpukuria Dopachari Wildlife Sanctuary	Sangu	22.245	92.1158	13 May 2015
101	Badidae	*Badis pallidus*	MK567722	NRM	67251	Kullander, SO	10567	Chittagong	Dopachari Stream, Dudpukuria, Dopachari Wildlife Sanctuary	Sangu	22.2277	92.1256	13 May 2015
101	Badidae	*Badis pallidus*	MK029378	NRM	67309	Kullander, SO	10574	Chittagong	Shaila Propat Waterfall and downstream pools	Sangu	22.1515	92.2163	14 May 2015
101	Badidae	*Badis pallidus*	MK029381	NRM	69436	Kullander, SO	11684	Chittagong	Shaila Propat hillstream	Sangu			13 Feb 2016
101	Badidae	*Badis pallidus*	MK029402	NRM	67671	Kullander, SO	10722	Chittagong	Small stream tributary to Sangu River,under bridge near Munlai Para village, E of Ruma	Sangu	22.0328	92.41753	14 May 2015
101	Badidae	*Badis pallidus*	MK029405	DU	6243	Kullander, SO	6243	Chittagong	Taichala Stream	Karnafuli	23.1038	91.96689	2015-04-26
102	Badidae	*Badis chittagongis*	MK029398	NRM	67448	Kullander, SO	10745	Chittagong	Maheshkhali Island, Khalmadia Chora, 5 km N of Maheshkhali Upazila	Bara Matamuhu	21.584	91.96534	11 May 2015
102	Badidae	*Badis chittagongis*	MK029400	NRM	67141	Kullander, SO	10599	Chittagong	Maheshkhali Island, Khaler Uttarkul, Barachora Stream	Bara Matamuhu	21.5141	91.96584	11 May 2015
102	Badidae	*Badis chittagongis*	MK029399	NRM	67404	Kullander, SO	10613	Chittagong	Barochora stream, 4 km south of Cox's Bazar	Bara Matamuhu	21.3959	92.00372	8 May 2015
102	Badidae	*Badis chittagongis*	MK029397	NRM	67831	Kullander, SO	10829	Chittagong	Kudung Cave in Teknaf Game Reserve		21.0923	92.16972	9 May 2015
102	Badidae	*Badis chittagongis*	MK029401	NRM	67891	Kullander, SO	10802	Chittagong	Maheshkhali Island, ravine about 7 km north of boat landing	Bara Matamuhu	21.5808	91.9631	11 May 2015
102	Badidae	*Badis chittagongis*	MK567708	NRM	67212	Kullander, SO	10539	Chittagong	Sonarpara Bazar, Delpara stream	Matamuhuri	21.2699	92.05768	9 May 2015
103	Badidae	*Badis rhabdotus*	MK029404	NRM	69034	Kullander, SO	11524	Sylhet	Piyain River at Jaflong	Meghna	25.1844	92.01642	24 Mar 2016
103	Badidae	*Badis rhabdotus*	MK029403	DU	9037	Kullander, SO	DU9037	Sylhet	Piyain River at Jaflong	Meghna	25.1844	92.01642	24 Mar 2016
104	Badidae	*Dario kajal*	MK029408	NRM	66171	Kullander, SO	11950	Sylhet	Fenchuganj, roadside ditch in Garuli floodplain, 4 km south of Sylhet	Meghna	24.6945	91.94553	22 Mar 2016
104	Badidae	*Dario kajal*	MK029407	NRM	66172	Kullander, SO	11949	Sylhet	Fenchuganj, roadside ditch in Garuli floodplain, 4 km south of Sylhet	Meghna	24.6945	91.94553	22 Mar 2016
105	Mastacembelida	*Macrognathus pancalus*	MK572318	NRM	66944	Kullander, SO	10039	Dhaka	Local fish market in Dhaka				1 Dec 2014

105	Mastacembelida	Macrognathus pancalus	MK572317	NRM	66915	Kullander, SO	10428	Chittagong	Rangamati, fish landing pier, fish from Kaptai Lake	Karnafuli	22.6501	92.18571	27-28 Nov 2014
105	Mastacembelida	Macrognathus pancalus	MK572319	NRM	69081	Kullander, SO	11373	Chittagong	Titas River in Akhaura	Meghna	23.8843	91.20121	19 Mar 2016
106	Mastacembelida	Macrognathus aral	MK572315	NRM	68392	Kullander, SO	12537	Sylhet	Fish Market in Golapgonj	Meghna			23 Mar 2016
106	Mastacembelida	Macrognathus aral	MK572316	DU	6246	Kullander, SO	6246	Mymenshing	Itna Haor	Jamuna	24.5309	90.2721	2014-10-02
107	Mastacembelida	Mastacembelus armatus	MK572323	NRM	67089	Kullander, SO	10253	Dhaka	Turag River at Kamarpare, near Dhaka city	Padma	23.8985	90.38438	1 Dec 2014
107	Mastacembelida	Mastacembelus armatus	MK572321	NRM	67713	Kullander, SO	11125	Chittagong	Rangamati, fish landing pier, fish from Kaptai Lake	Karnafuli	22.6501	92.18571	27-28 Nov 2014
107	Mastacembelida	Mastacembelus armatus	MK572324	NRM	66831	Kullander, SO	10199	Chittagong	Bangchora hill stream in Kaptai, near hydropower station	Karnafuli	22.4958	92.18671	28 Nov 2014
107	Mastacembelida	Mastacembelus armatus	MK572320	NRM	69293	Kullander, SO	11488	Sylhet	Piyain River at Jaflong	Meghna	25.1844	92.01642	24 Mar 2016
107	Mastacembelida	Mastacembelus armatus	MK572322	NRM	67088	Kullander, SO	10251	Dhaka	Turag River at Kamarpare, near Dhaka city	Padma	23.8985	90.38438	1 Dec 2014
107	Mastacembelida	Mastacembelus armatus	MK572325	NRM	68224	Kullander, SO	11345	Chittagong	Kokhiyon Jhiri stream, 11 km W of Ruma	Sangu	22.058	92.34182	14 May 2015
108	Chaudhuriidae	Pillaia sp	MK572487	NRM	67690	Kullander, SO	10708	Chittagong	Small stream tributary to Sangu River,under bridge near Munlai Para village, Et of Ruma	Sangu	22.0328	92.41753	14 May 2015
108	Chaudhuriidae	Pillaia sp	MK572486	NRM	67689	Kullander, SO	10710	Chittagong	Small stream tributary to Sangu River,under bridge near Munlai Para village, E of Ruma	Sangu	22.0328	92.41753	14 May 2015
109	Cyprinidae	Pethia sp aff guganio	MK572463	NRM	66125	Kullander, SO	11929	Sylhet	Small canal near Jaintiapur on Sylhet-Jaflong road N2	Meghna	25.1519	92.11258	24 Mar 2016
109	Cyprinidae	Pethia sp aff guganio	MK572528	NRM	69361	Kullander, SO	11569	Sylhet	Surma River left bank, at Kheaghat point, 1,5 km upstream from Golapganj	Meghna	24.8612	91.99371	23 Mar 2016
110	Cyprinidae	Pethia guganio	MK572475	NRM	67758	Kullander, SO	10935	Chittagong	Udalbania, small stream left bank tributary to Karnafuli River, tobacco plantation	Karnafuli	22.3245	92.139	12 May 2015
110	Cyprinidae	Pethia guganio	MK572478	NRM	67756	Kullander, SO	10939	Chittagong	Udalbania, small stream left bank tributary to Karnafuli River,h tobacco plantation	Karnafuli	22.3245	92.139	12 May 2015
110	Cyprinidae	Pethia guganio	MK572476	NRM	66665	Kullander, SO	9952	Chittagong	Kamila stream, tributary to Karnafuli River, 22 km south of Rangamati	Karnafuli	22.5281	92.19861	28 Nov 2014
110	Cyprinidae	Pethia guganio	MK572474	NRM	67326	Kullander, SO	10465	Chittagong	Udalbania, stream in Dudpukuria-Dhopachori Wildlife Sanctuary	Karnafuli	22.3102	92.15844	12 May 2015
111	Cyprinidae	Pethia guganio	MK572477	NRM	66963	Kullander, SO	10021	Dhaka	Padma River near Srinagar	Padma			2 Dec 2014
112	Cyprinidae	Pethia gelius	MK572472	NRM	69033	Kullander, SO	11523	Sylhet	Piyain River at Jaflong	Meghna	25.1844	92.01642	24 Mar 2016
112	Cyprinidae	Pethia gelius	MK572473	NRM	66968	Kullander, SO	10339	Dhaka	Padma River near Srinagar	Padma			2 Dec 2014
112	Cyprinidae	Pethia gelius	MK572470	NRM	66591	Kullander, SO	9781	Dhaka	Fish market in Shonbari, Sreenagar	Padma	23.544	90.29635	2 Dec 2014
112	Cyprinidae	Pethia gelius	MK572471	DU	6241	Kullander, SO	6241	Mymenshing	Sherpur market	Jamuna	25.0229	90.01728	2015-03-21
113	Cyprinidae	Pethia conchonius	MK572465	NRM	67322	Kullander, SO	10486	Chittagong	Udalbania, stream in Dudpukuria-Dhopachori Wildlife Sanctuary	Karnafuli	22.3102	92.15844	12 May 2015
113	Cyprinidae	Pethia conchonius	MK572468	NRM	66570	Kullander, SO	9793	Chittagong	Woagga Stream, 4 km W of Kaptai National Park	Karnafuli	22.5091	92.14182	28 Nov 2014
113	Cyprinidae	Pethia conchonius	MK572467	NRM	66538	Kullander, SO	9822	Dhaka	Market at Ashulia Point, Turag River	Padma			1 Dec 2014
113	Cyprinidae	Pethia conchonius	MK572469	NRM	66708	Kullander, SO	9985	Chittagong	Boga Lake	Sangu	21.9806	92.47478	11 Jun 2014
113	Cyprinidae	Pethia conchonius	MK572464	NRM	66725	Kullander, SO	10099	Rangpur	Atrai River at Dhaspara village, 8 km N of Khanshama	Atrai	26.0003	88.71325	3 May 2014
113	Cyprinidae	Pethia conchonius	MK572466	NRM	66712	Kullander, SO	10122	Chittagong	Upper Sangu River at Remacri point	Sangu	21.679	92.51936	9 Jun 2014
114	Cyprinidae	Pethia rutila	MK572481	NRM	67239	Kullander, SO	10471	Chittagong	Kuikhali, small pool close to trail from Kudong Cave		21.0938	92.16811	9 May 2015
114	Cyprinidae	Pethia rutila	MK572484	NRM	67837	Kullander, SO	10879	Chittagong	Kudung Cave in Teknaf Game Reserve		21.0923	92.16972	9 May 2015
114	Cyprinidae	Pethia rutila	MK572482	NRM	66666	Kullander, SO	9949	Chittagong	Kamila stream, tributary to Karnafuli River, 22 km south of Rangamati	Karnafuli	22.5281	92.19861	28 Nov 2014
114	Cyprinidae	Pethia rutila	MK572483	NRM	67895	Kullander, SO	10903	Chittagong	Maheshkhali Island, ravine about 7 km north of boat landing	Bara Matamuhu	21.5808	91.9631	11 May 2015
115	Cyprinidae	Pethia ticto	MK572485	NRM	66970	Kullander, SO	10350	Dhaka	Padma River near Srinagar	Padma			2 Dec 2014
116	Cyprinidae	Puntius phutunio	MK572485	NRM	68808	Kullander, SO	11407	Sylhet	Hakaluki Haor in Borolek	Meghna	24.6617	92.03478	26 Mar 2016
116	Cyprinidae	Pethia phutunio	MK572480	DU	6197	Kullander, SO	6197	Mymenshing	Sherpur market	Jamuna	25.0229	90.01728	2015-03-21
117	Cyprinidae	Garra sp SHERPUR	MK572205	DU	6203	Kullander, SO	6203	Mymenshing	Chilakhali stream	Jamuna	25.2543	90.03265	2015-03-21
118	Cyprinidae	Garra sp cf Koladynensis	MK572209	NRM	66036	Kullander, SO	11839		Piyain River at Jaflong	Meghna	25.1844	92.01642	24 Mar 2016
119	Cyprinidae	Garra sp MEGHNA	MK572210	NRM	65566	Kullander, SO	9875		Locality data pending	Meghna			Unknown date
120	Cyprinidae	Garra vittatula	MK572213	NRM	66709	Kullander, SO	9992	Chittagong	pools at Chingthong and Shangham Jhiri water falls in near Remacri canal	Sangu	21.6911	92.5227	10 Jun 2014
120	Cyprinidae	Garra vittatula	MK572214	NRM	67693	Kullander, SO	10756	Chittagong	Small stream tributary to Sangu River,under bridge near Munlai Para village, E of Ruma	Sangu	22.0328	92.41753	14 May 2015
120	Cyprinidae	Garra vittatula	MK572215	NRM	67693	Kullander, SO	11323	Chittagong	Small stream tributary to Sangu River,under bridge near Munlai Para village, E of Ruma	Sangu	22.0328	92.41753	14 May 2015
120	Cyprinidae	Garra vittatula	MK572212	DU	9012	Kullander, SO	9012	Chittagong	Shangom Jhiri Remacri	Sangu			
121	Cyprinidae	Garra mini	KT223104	NRM	66710	Kullander, SO	10101	Chittagong	pools at Chingthong and Shangham Jhiri water falls near Remacri canal	Sangu	21.6911	92.5227	10 Jun 2014
121	Cyprinidae	Garra mini	KT223105	NRM	66722	Kullander, SO	10121	Chittagong	Shuvolong Water Falls	Karnafuli			10 Apr 2014
121	Cyprinidae	Garra mini	KT223107	NRM	66745	Kullander, SO	10057	Chittagong	Borokal, pool at bottom of Shuvolong Waterfall	Karnafuli	22.7143	92.2477	28 Nov 2014
121	Cyprinidae	Garra mini	KT223106	NRM	66746	Kullander, SO	10058	Chittagong	Borokal, pool at bottom of Shuvolong Waterfall	Karnafuli	22.7143	92.2477	28 Nov 2014
121	Cyprinidae	Garra mini	MK572211	NRM	66748	Kullander, SO	10168	Chittagong	Borokal, pool at bottom of Shuvolong Waterfall	Karnafuli	22.7143	92.2477	28 Nov 2014
121	Cyprinidae	Garra mini	KT272021	DU	6240	Kullander, SO	6240	Chittagong	Shailopropat Waterfalls	Sangu	22.1509	92.20908	2015-05-05
122	Cyprinidae	Garra dampaensis	MK572206	NRM	66711	Kullander, SO	10106	Chittagong	pools at Chingthong and Shangham Jhiri water falls near Remacri canal	Sangu	21.6911	92.5227	10 Jun 2014
122	Cyprinidae	Garra dampaensis	MK572207	NRM	67685	Kullander, SO	10709	Chittagong	Small stream tributary to Sangu River,under bridge near Munlai Para village, E of Ruma	Sangu	22.0328	92.41753	14 May 2015
122	Cyprinidae	Garra dampaensis	MK572208	NRM	67311	Kullander, SO	10576	Chittagong	Shaila Propat Waterfall and downstream pools	Sangu	22.1515	92.2163	14 May 2015
123	Cyprinidae	Puntius terio	MK572537	NRM	66663	Kullander, SO	9908	Chittagong	Kamila stream, tributary to Karnafuli River, 22 km south of Rangamati	Karnafuli	22.5281	92.19861	28 Nov 2014
123	Cyprinidae	Putius terio	MK572536	NRM	52296	Kullander, SO	2347	Khulna	University of Khulna, ponds on campus	Padma	22.8035	89.53458	2005
123	Cyprinidae	Puntius terio	MK572535	NRM	66586	Kullander, SO	9836	Dhaka	Fish market in Shonbari, Sreenagar	Padma	23.544	90.29635	2 Dec 2014
124	Cyprinidae	Puntius chola	MK572530	NRM	66510	Kullander, SO	10129	Rangpur	Tangoan River, Dumurighat point of Rangon Union, 10 km south of Bochagonj	Mahananda	25.7339	88.42311	4 May 2014
125	Cyprinidae	Pethia sophore	MK572533	NRM	66041	Kullander, SO	11838	Khulna	Darhampur, large roadside pond	Padma	24.7693	91.91394	22 Mar 2016
125	Cyprinidae	Puntius sophore	MK572531	NRM	66502	Kullander, SO	10133	Rangpur	Tista barrage area, about 7 km E of Dimla Upazila	Tista River	26.1802	89.03997	2 May 2014
125	Cyprinidae	Puntius sophore	MK572532	NRM	66740	Kullander, SO	9996	Rangpur	Tangoan River, Dumurighat point of Rangon Union, 10 km south of Bochagonj	Mahananda	25.7339	88.42311	4 May 2014
125	Cyprinidae	Puntius sophore	MK572534	NRM	67086	Kullander, SO	10252	Dhaka	Turag River at Kamarpare, near Dhaka city	Padma	23.8985	90.38438	1 Dec 2014
126	Cyprinidae	Osteobrama cf cotio	MG895646	NRM	67175	Kullander, SO	10553	Chittagong	Sangu River at Dhopachari	Sangu	22.2061	92.13399	13 May 2015
126	Cyprinidae	Osteobrama cf cotio	MG895647	NRM	66545	Kullander, SO	10196	Chittagong	Rangamati, town fish market, fish principally from Kaptai Lake	Karnafuli	22.6497	92.18372	27 Nov 2014
127	Cyprinidae	Osteobrama cotio	MG895645	NRM	69291	Kullander, SO	11486	Sylhet	Kushiyara River left bank in Fenchuganj at junction with Juri River	Meghna	24.7054	91.95432	22 Mar 2016
127	Cyprinidae	Osteobrama cotio	MG895644	NRM	69352	Kullander, SO	11566	Dhaka	Meghna River at Ashuganj Point	Meghna	24.0463	91.00562	20 Mar 2016
128	Cyprinidae	Systomus sarana	MH708075	NRM	66723	Kullander, SO	9983	Rangpur	Atrai River at Dhaspara village, 8 km N of Khanshama	Padma	26.0003	88.71325	3 May 2014
128	Cyprinidae	Systomus sarana	MH708074	NRM	66512	Kullander, SO	10131	Sylhet	Pyiang River at Jaflong, about 60 km from Sylhet city	Meghna	25.1862	92.01667	25 Apr 2014
129	Cyprinidae	Barbonymus gonionotus	MK572052	NRM	52297	Reichard, M	2348	Khulna	University of Khulna, ponds on campus	Padma	22.8035	89.53458	2005
129	Cyprinidae	Barbonymus gonionotus	MK572051	DU	6085	Rahman, MM	6085	Dhaka	Kaoran Bazar		23.7507	90.39568	6 apr 2014
130	Cyprinidae	Oreichthys cosuatis	MK572404	NRM	66971	Kullander, SO	10343	Dhaka	Padma River near Srinagar	Padma			2 Dec 2014
130	Cyprinidae	Oreichthys cosuatis	MK572403	NRM	69346	Kullander, SO	11579	Sylhet	Surma River left bank, at Kheaghat point, 1,5 km upstream from Golapganj	Meghna	24.8612	91.99371	23 Mar 2016
131	Cyprinidae	Labeo rohita	MK572283	NRM	68366	Kullander, SO	12237	Sylhet	Hakaluki Haor in Borolek	Meghna	24.6617	92.03478	26 Mar 2016

132	Cyprinidae	*Labeo catla*	MK572219	DU	6233	Rahman, MM	6233	Mymenshing	Sherpur market	Jamuna	25.0229	90.01728	2015-03-21
133	Cyprinidae	*Labeo angra*	MK572273	NRM	66733	Kullander, SO	9997	Rangpur	Tangoan River, Dumurighat point of Rangon Union, 10 km south of Bochagonj	Mahananda	25.7339	88.42311	4 May 2014
134	Cyprinidae	*Labeo calbasu*	MK572279	NRM	67629	Kullander, SO	11140	Chittagong	Rangamati, fish landing pier, fish from Kaptai Lake	Karnafuli	22.6501	92.18571	27-28 Nov 2014
134	Cyprinidae	*Labeo calbasu*	MK572281	NRM	66717	Kullander, SO	9991	Dhaka	Someshwari River in Durgapur	Jamuna	25.1092	90.67818	4 Apr 2014
134	Cyprinidae	*Labeo calbasu*	MK572280	NRM	67751	Kullander, SO	11151	Dhaka	Fish market in Shonbari, Sreenagar	Padma	23.544	90.29635	2 Dec 2014
135	Cyprinidae	*Labeo gonius*	MG895641	NRM	67602	Kullander, SO	11046	Dhaka	Local fish market in Dhaka				1 Dec 2014
135	Cyprinidae	*Labeo gonius*	MK572282	DU	6008	Rahman, MM	6008	Mymenshing	Itna Haor	Jamuna	24.5309	91.10439	###########
136	Cyprinidae	*Labeonini sp*	MK572145	DU	9034	Kullander, SO	9034	Mymenshing	Batpara point Jamuna River				
137	Cyprinidae	*Cirrhinus cirrhosus*	MK572125	NRM	68952	Kullander, SO	12540	Sylhet	Fish Market in Golapgonj	Meghna			23 Mar 2016
137	Cyprinidae	*Cirrhinus cirrhosus*	MK572124	DU	6268	Rahman, MM	DU6268		Bangladesh, no further data saved				
137	Cyprinidae	*Cirrhinus cirrhosus*	MK572126	DU	6133	Rahman, MM	6133	Mymenshing	Itna Haor	Jamuna	24.5309	91.10439	2014-10-02
138	Cyprinidae	*Labeo boggut*	MK572277	NRM	66732	Kullander, SO	9989	Rangpur	Tangoan River, Dumurighat point of Rangon Union, 10 km south of Bochagonj	Mahananda	25.7339	88.42311	4 May 2014
138	Cyprinidae	*Labeo boggut*	MK572276	NRM	66724	Kullander, SO	9990	Rangpur	Tista barrage area, about 7 km E of Dimla Upazila	Tista	26.1802	89.03997	2 May 2014
138	Cyprinidae	*Labeo boggut*	MK572278	NRM	66735	Kullander, SO	10112	Rangpur	Kortoa River, at Bogorbari point, under Teprigonj union, about 5 km N of Devigonj town	Padma	26.1375	88.71884	3 May 2014
139	Cyprinidae	*Labeo boga*	MK572274	NRM	66718	Kullander, SO	10100	Dhaka	Someshwari River in Durgapur	Samuna	25.1092	90.67818	4 Apr 2014
139	Cyprinidae	*Labeo boga*	MK572275	NRM	66734	Kullander, SO	10137	Rangpur	Kortoa River, at Bogorbari point, under Teprigonj union, about 5 km N of Devigonj town	Padma	26.1375	88.71884	3 May 2014
140	Cyprinidae	*Gymnostomus ariza*	MK572253	NRM	66726	Kullander, SO	10104	Rangpur	Atrai River at Dhaspara village, 8 km N of Khanshama	Padma	26.0003	88.71325	3 May 2014
140	Cyprinidae	*Gymnostomus ariza*	MK572255	NRM	66513	Kullander, SO	10132	Sylhet	Pyiang River at Jaflong, about 60 km from Sylhet city	Meghna	25.1862	92.01667	25 Apr 2014
140	Cyprinidae	*Gymnostomus ariza*	MK572256	NRM	66743	Kullander, SO	10111		Various localities, TBA				2014
140	Cyprinidae	*Gymnostomus ariza*	MK572254	NRM	66719	Kullander, SO	10105	Dhaka	Someshwari River in Durgapur	Jamuna	25.1092	90.67818	4 Apr 2014
141	Cyprinidae	*Cyprinus carpio*	MK572147	DU	6096	Rahman, MM	6096	Dhaka	Kaoran Bazar		23.7507	90.39568	2015-01-30
141	Cyprinidae	*Cyprinus carpio*	MK572146	DU	6079	Rahman, MM	6079	Dhaka	Kaoran Bazar				2015-04-06
142	Cyprinidae	*Tariqilabeo latius*	MK572607	NRM	69280	Kullander, SO	11475	Sylhet	Kushiyara River left bank in Fenchuganj at junction with Juri River	Meghna	24.7054	91.95432	22 Mar 2016
142	Cyprinidae	*Tariqilabeo latius*	MK572609	NRM	67002	Kullander, SO	10030	Dhaka	Padma River near Srinagar	Padma			2 Dec 2014
142	Cyprinidae	*Tariqilabeo latius*	MK572608	NRM	66505	Kullander, SO	10136	Rangpur	Tista barrage area, about 7 km E of Dimla Upazila	Tista	26.1802	89.03997	2 May 2014
143	Cyprinidae	*Neolissochilus stracheyi*	MK572367	NRM	66713	Kullander, SO	10123	Chittagong	pools at Chingthong and Shangham Jhiri water falls near Remacri canal	Sangu	21.6911	92.5227	10 Jun 2014
143	Cyprinidae	*Neolissochilus stracheyi*	MK572366	NRM	66714	Kullander, SO	10124	Chittagong	pools at Chingthong and Shangham Jhiri water falls near Remacri canal	Sangu	21.6911	92.5227	10 Jun 2014
143	Cyprinidae	*Neolissochilus stracheyi*	MK572365	DU	6110	Kullander, SO	6110	Chittagong	Mohamaya Hill stream		22.8185	91.57269	2015-05-31
144	Cyprinidae	*Chagunius chagunio*	MK572093	NRM	68873	Kullander, SO	12202	Sylhet	Piyain River at Jaflong	Meghna	25.1844	92.01642	24 Mar 2016
144	Cyprinidae	*Chagunius chagunio*	MK572094	DU	6212	Rahman, MM	6212	Mymenshing	Chilakhali stream	Jamuna	25.2543	90.03265	2015-03-21
145	Cyprinidae	*Ctenopharyngodon idella*	MK572141	NRM	67631	Kullander, SO	11154	Dhaka	Fish market in Shonbari, Sreenagar	Padma	23.544	90.29635	2 Dec 2014
145	Cyprinidae	*Ctenopharyngodon idella*	MK572142	DU	6099	Rahman, MM	6099	Dhaka	Kaoran Bazar		23.7507	90.39568	2015-01-30
146	Cyprinidae	*Mylopharyngodon piceus*	MK572333	DU	6081	Rahman, MM	6081	Dhaka	Swapna Super Market				2015-02-26
147	Cyprinidae	*Hypophthalmichthys molitrix*	MK572264	DU	6097	Rahman, MM	6097	Dhaka	Kaoran Bazar		23.7507	90.39568	2015-01-30
147	Cyprinidae	*Hypophthalmichthys nobilis*	MK572265	DU	6098	Rahman, MM	6097	Dhaka	Kaoran Bazar		23.7507	90.39568	2015-01-30
149	Cyprinidae	*Rasbora rasbora*	MK572544	NRM	66619	Kullander, SO	9811	Dhaka	Fish market in Shonbari, Sreenagar	Padma	23.544	90.29635	2 Dec 2014
149	Cyprinidae	*Rasbora rasbora*	MK572545	NRM	67865	Kullander, SO	10909	Chittagong	Donumia Stream, 10 km north of Teknaf town, 70 km south of Cox's Bazar	Naf	20.9233	92.26298	9 May 2015
149	Cyprinidae	*Rasbora rasbora*	MK572546	NRM	67864	Kullander, SO	10913	Chittagong	Donumia Stream, 10 km north of Teknaf town, 70 km south of Cox's Bazar	Naf	20.9233	92.26298	9 May 2015
150	Cyprinidae	*Amblypharyngodon mola*	MK572021	NRM	67207	Kullander, SO	10592	Chittagong	Sonarpara Bazar, Delpara stream		21.2699	92.05768	9 May 2015
150	Cyprinidae	*Amblypharyngodon mola*	MK572017	NRM	66695	Kullander, SO	9935	Dhaka	Turag River at Kamarpare, near Dhaka city	Padma	23.8985	90.38438	1 Dec 2014
150	Cyprinidae	*Amblypharyngodon mola*	MK572022	NRM	66696	Kullander, SO	9958	Dhaka	Turag River at Kamarpare, near Dhaka city	Padma	23.8985	90.38438	1 Dec 2014
150	Cyprinidae	*Amblypharyngodon mola*	MK572020	NRM	68328	Kullander, SO	12189	Khulna	Darhampur, large roadside pond	Padma	24.7693	91.91394	22 Mar 2016
150	Cyprinidae	*Amblypharyngodon mola*	MK572018	DU	6087	Rahman, MM	6087	Dhaka	Mohammadpur Bazar		23.7507	90.39568	2015-02-22
150	Cyprinidae	*Amblypharyngodon mola*	MK572019	DU	6086	Rahman, MM	6086	Dhaka	Mohammadpur Bazar		23.7507	90.39568	2015-02-22
151	Cyprinidae	*Rasbora daniconius*	MK572543	NRM	69002	Kullander, SO	11395	Sylhet	Hail Haor, Baikka Beel, at tourist center	Meghna	24.3619	91.70741	21 Mar 2016
151	Cyprinidae	*Rasbora daniconius*	MK572542	NRM	67828	Kullander, SO	10835	Chittagong	Kudung Cave in Teknaf Game Reserve		21.0923	92.16972	9 May 2015
151	Cyprinidae	*Rasbora daniconius*	MK572541	NRM	66749	Kullander, SO	10376	Chittagong	Roadside ditch in Sultanpur	Karnafuli	22.5422	91.8997	29 Nov 2014
151	Cyprinidae	*Rasbora daniconius*	MK572540	NRM	66645	Kullander, SO	9909	Chittagong	Borokal, small stream drainging pool below Shuvolong waterfall	Karnafuli	22.7143	92.2477	28 Nov 2014
152	Cyprinidae	*Devario aequipinnatus*	MF12750	NRM	66258	Kullander, SO	9865	Sylhet	Madokundo waterfall, Moulovi Bazar. Aquarium specimens from the wild	Meghna			Unknown date
152	Cyprinidae	*Devario anomalus* HYBRID	MF172758	NRM	67936	Kullander, SO	10786	Chittagong	Mrong Bazar, small stream tributary to Sangu River, below bridge	Sangu	22.0574	92.31547	14 May 2015
152	Cyprinidae	*Devario anomalus* HYBRID	MF172757	NRM	67937	Kullander, SO	10787	Chittagong	Mrong Bazar, small stream tributary to Sangu River, below bridge	Sangu	22.0574	92.31547	14 May 2015
152	Cyprinidae	*Devario aequipinnatus*	MF172746	NRM	66259	Kullander, SO	9866	Sylhet	Madokundo waterfall, Moulovi Bazar. Aquarium specimens from the wild	Meghna			Unknown date
152	Cyprinidae	*Devario aequipinnatus*	MF172744	NRM	66832	Kullander, SO	10198	Chittagong	Bangchora hill stream in Kaptai, near hydropower station	Karnafuli	22.4958	92.18671	28 Nov 2014
152	Cyprinidae	*Devario aequipinnatus*	MF172745	NRM	66705	Kullander, SO	10126	Sylhet	Madopkundo water falls, hill streams about 5 km from Dakshinbagh railway station	Meghna	24.5547	92.224	26 Apr 2014
152	Cyprinidae	*Devario coxi*	MF172763	NRM	67378	Kullander, SO	10543	Chittagong	Majerchora Stream, 10 km south of Cox's Bazar	Bakkhali	21.3958	92.00455	8 May 2015
152	Cyprinidae	*Devario coxi*	MF172779	NRM	67379	Kullander, SO	10544	Chittagong	Majerchora Stream, 10 km south of Cox's Bazar	Bakkhali	21.3958	92.00455	8 May 2015
152	Cyprinidae	*Devario aequipinnatus*	MF172754	NRM	66813	Kullander, SO	10223	Chittagong	Hillstream at northwestern margin of Chittagong University campus, in and below rapids	Karnafuli	22.4738	91.78312	29 Nov 2014
152	Cyprinidae	*Devario aequipinnatus*	MF172753	DU	6188	Kullander; SO	6188	Chittagong	KhagrachoriSadar: Bangmara stream beside Chittagong-Khagrachori highway	Karnafuli	23.0644	91.90538	2015-04-24
152	Cyprinidae	*Devario aequipinnatus*	MF172751	DU	6253	Kullander; SO	6253	Chittagong	Mohamaya Hill stream		22.8185	91.57269	2015-05-31
152	Cyprinidae	*Devario aequipinnatus*	MF172752	DU	6244	Kullander; SO	6244	Chittagong	Richong Stream	Karnafuli	23.0664	91.94392	2015-04-24
153	Cyprinidae	*Devario anomalus*	MF172755	NRM	67399	Kullander, SO	10659	Chittagong	Barochora stream, 4 km south of Cox's Bazar		21.3959	92.00372	8 May 2015
154	Cyprinidae	*Devario devario*	MF172766	NRM	66260	Kullander, SO	9870	Dhaka	Fish market in Shonbari, Sreenagar	Padma	23.544	90.29635	2 Dec 2014
154	Cyprinidae	*Devario devario*	MF172764	NRM	66262	Kullander, SO	9867	Dhaka	Fish market in Shonbari, Sreenagar	Padma	23.544	90.29635	2 Dec 2014
154	Cyprinidae	*Devario devario*	MF172765	NRM	66730	Kullander, SO	10119	Rangpur	Tista barrage area, about 7 km E of Dimla Upazila	Tista	26.1802	89.03997	2 May 2014
155	Cyprinidae	*Chela cachius*	MG895633	NRM	66988	Kullander, SO	10085	Dhaka	Padma River near Srinagar				2 Dec 2014
156	Cyprinidae	*Chela cachius*	MG895632	DU	6116	Rahman, MM	6116	Chittagong	Kohua River		22.9118	91.50275	2015-06-03
157	Cyprinidae	*Laubuka laubuca*	MG895634	NRM	67317	Kullander, SO	10455		Aquarium shops in Dhaka, said to be local fish				16 May 2015
157	Cyprinidae	*Laubuka laubuca*	MG895635	NRM	67315	Kullander, SO	10453		Aquarium shops in Dhaka, said to be local fish				16 May 2015
158	Cyprinidae	*Laubuka tenella*	MG895638	NRM	67845	Kullander, SO	10914	Chittagong	Donumia Stream, 10 km north of Teknaf town, 70 km south of Cox's Bazar	Naf	20.9233	92.26298	9 May 2015
158	Cyprinidae	*Laubuka tenella*	MG895639	NRM	67862	Kullander, SO	10908	Chittagong	Donumia Stream, 10 km north of Teknaf town, 70 km south of Cox's Bazar	Naf	20.9233	92.26298	9 May 2015
158	Cyprinidae	*Laubuka tenella*	MG895640	NRM	67380	Kullander, SO	10542	Chittagong	Majerchora Stream, 10 km south of Cox's Bazar	Bakkhali	21.3958	92.00455	8 May 2015

158	Cyprinidae	*Laubuka tenella*	MG895637	NRM	67381	Kullander, SO	10563	Chittagong	Majerchora Stream, 10 km south of Cox's Bazar	Bakkhali	21.3958	92.00455	8 May 2015
159	Cyprinidae	*Danio annulosus*	KT199744	NRM	66253	Kullander, SO	9869	Chittagong	Borokal, pool at bottom of Shuvolong Waterfall	Karnafuli	22.7143	92.2477	28 Nov 2014
159	Cyprinidae	*Danio annulosus*	KT199745	NRM	66254	Kullander, SO	10017	Chittagong	Borokal, pool at bottom of Shuvolong Waterfall	Karnafuli	22.7143	92.2477	28 Nov 2014
159	Cyprinidae	*Danio annulosus*	KT199746	NRM	66255	Kullander, SO	10020	Chittagong	Borokal, pool at bottom of Shuvolong Waterfall	Karnafuli	22.7143	92.2477	28 Nov 2014
159	Cyprinidae	*Danio annulosus*	MK572148	NRM	67351	Kullander, SO	10550	Chittagong	Kudung Cave in Teknaf Game Reserve		21.0923	92.16972	9 May 2015
160	Cyprinidae	*Danio rerio*	MK572160	NRM	66511	Kullander, SO	10130	Dhaka	Bhabanipur, hill streams about 5 km north of Durgarpur Upazila	Jamuna	25.1899	90.66049	5 Apr 2014
160	Cyprinidae	*Danio rerio*	MK572158	DU	6234	Kullander, SO	6234	Mymenshing	Chilakhali stream	Jamuna	25.2543	90.03265	2015-03-21
160	Cyprinidae	*Danio rerio*	MK572159	DU	6211	Kullander, SO	6211	Mymenshing	Chilakhali stream	Jamuna	25.2543	90.03265	2015-03-21
161	Cyprinidae	*Danio* cf *rerio*	MK572157	NRM	67327	Kullander, SO	10487	Chittagong	Udalbania, stream in Dudpukuria-Dhopachori Wildlife Sanctuary	Karnafuli	22.3102	92.15844	12 May 2015
161	Cyprinidae	*Danio* cf *rerio*	MK572151	NRM	66257	Kullander, SO	9948	Chittagong	Hillstream at northwestern margin of Chittagong University campus, in and below rapids	Karnafuli	22.4738	91.78312	29 Nov 2014
161	Cyprinidae	*Danio* cf *rerio*	MK572152	NRM	66256	Kullander, SO	9947	Chittagong	Hillstream at northwestern margin of Chittagong University campus, in and below rapids	Karnafuli	22.4738	91.78312	29 Nov 2014
161	Cyprinidae	*Danio* cf *rerio*	MK572153	DU	6254	Kullander, SO	DU6254		Bangladesh, no furthr data saved				
161	Cyprinidae	*Danio* cf *rerio*	MK572149	NRM	67213	Kullander, SO	10540	Chittagong	Sonarpara Bazar, Delpara stream		21.2699	92.05768	9 May 2015
161	Cyprinidae	*Danio* cf *rerio*	MK572156	NRM	67268	Kullander, SO	10503	Chittagong	Myani Stream in Dudpukuria Dopachari Wildlife Sanctuary	Sangu	22.245	92.1158	13 May 2015
161	Cyprinidae	*Danio* cf *rerio*	MK572150	DU	6237	Kullander, SO	6237	Chittagong	Mohamaya Hill stream			91.57269	2015-05-31
161	Cyprinidae	*Danio* cf *rerio*	MK572154	DU	6251	Kullander, SO	6251	Chittagong	Shailopropat stream	Sangu	22.1509	92.20908	2015-05-15
161	Cyprinidae	*Danio* cf *rerio*	MK572155	DU	6176	Kullander, SO	6176	Chittagong	Doulla khal	Karnafuli	22.7858	91.80491	2015-04-24
162	Cyprinidae	*Danio* sp BANGLADESH	MK572162	NRM	67241	Kullander, SO	10466	Chittagong	Teknaf Wildlife Park, small stream	Naf	20.932	92.25827	10 May 2015
162	Cyprinidae	*Danio* sp BANGLADESH	MK572161	NRM	67242	Kullander, SO	10467	Chittagong	Teknaf Wildlife Park, small stream	Naf	20.932	92.25827	10 May 2015
163	Cyprinidae	*Esomus danrica*	MK572186	NRM	67203	Kullander, SO	10590	Chittagong	Sonarpara Bazar, Delpara stream		21.2699	92.05768	9 May 2015
163	Cyprinidae	*Esomus danrica*	MK572188	NRM	66641	Kullander, SO	9910	Chittagong	Borokal, small stream drainging pool below Shuvolong waterfall	Karnafuli	22.7143	92.2477	28 Nov 2014
163	Cyprinidae	*Esomus danrica*	MK572189	NRM	69032	Kullander, SO	11522	Sylhet	Small roadside pool near Jaflongon Sylhet-Jaflong road N2	Meghna	25.178	92.04595	24 Mar 2016
163	Cyprinidae	*Esomus danrica*	MK572187	NRM	66688	Kullander, SO	9931	Dhaka	Turag River at Kamarpare, near Dhaka city	Padma	23.8985	90.38438	1 Dec 2014
163	Cyprinidae	*Esomus danrica*	MK572191	NRM	67350	Kullander, SO	10551	Chittagong	Kudung Cave in Teknaf Game Reserve		21.0923	92.16972	9 May 2015
163	Cyprinidae	*Esomus danrica*	MK572190	NRM	67438	Kullander, SO	10663	Chittagong	Maheshkhali Island, Khalmadia Chora, 5 km N of Maheshkhali Upazila	Bara Matamuhu	21.584	91.96534	11 May 2015
164	Psilorhynchidae	*Psilorhynchus nudithoracius*	MK572513	NRM	65789	Kullander, SO	12073	Sylhet	Surma River left bank, at Kheaghat point, 1,5 km upstream from Golapganj	Meghna	24.8612	91.99371	23 Mar 2016
164	Psilorhynchidae	*Psilorhynchus nudithoracicus*	MK572515	NRM	66715	Kullander, SO	10125	Chittagong	Upper Sangu River at Remacri point	Sangu	21.679	92.51936	9 Jun 2014
164	Psilorhynchidae	*Psilorhynchus nudithoracius*	MK572511	NRM	68244	Kullander, SO	11292	Chittagong	Kokhiyon Jhiri stream, 11 km W of Ruma	Sangu	22.058	92.34182	14 May 2015
164	Psilorhynchidae	*Psilorhynchus nudithoracius*	MK572512	NRM	68243	Kullander, SO	11291	Chittagong	Kokhiyon Jhiri stream, 11 km W of Ruma	Sangu	22.058	92.34182	14 May 2015
165	Psilorhynchidae	*Psilorhynchus sucatio*	MK572521	NRM	66504	Kullander, SO	10135	Rangpur	Atrai River at Dhaspara village, 8 km N of Khanshama	Padma	26.0003	88.71325	3 May 2014
166	Psilorhynchidae	*Psilorhynchus sucatio*	MK572518	NRM	66797	Kullander, SO	10298	Chittagong	Hillstream at northwestern margin of Chittagong University campus, in and below rapids	Karnafuli	22.4738	91.78312	29 Nov 2014
166	Psilorhynchidae	*Psilorhynchus sucatio*	MK572522	NRM	66802	Kullander, SO	10181	Chittagong	Hillstream at northwestern margin of Chittagong University campus, in and below rapids	Karnafuli	22.4738	91.78312	29 Nov 2014
166	Psilorhynchidae	*Psilorhynchus sucatio*	MK572519	NRM	66810	Kullander, SO	10184	Chittagong	Hillstream at northwestern margin of Chittagong University campus, in and below rapids	Karnafuli	22.4738	91.78312	29 Nov 2014
166	Psilorhynchidae	*Psilorhynchus sucatio*	MK572520	NRM	67952	Kullander, SO	10807	Chittagong	Mrong Bazar, small stream tributary to Sangu River, below bridge	Sangu	22.0574	92.31547	14 May 2015
167	Psilorhynchidae	*Psilorhynchus rahmani*	MK572517	NRM	66796	Kullander, SO	10226	Chittagong	Hillstream at northwestern margin of Chittagong University campus, in and below rapids	Karnafuli	22.4738	91.78312	29 Nov 2014
167	Psilorhynchidae	*Psilorhynchus rahmani*	MK572516	NRM	66804	Kullander, SO	10187	Chittagong	Hillstream at northwestern margin of Chittagong University campus, in and below rapids	Karnafuli	22.4738	91.78312	29 Nov 2014
168	Psilorhynchidae	*Psilorhynchus balitora*	MK572510	NRM	65625	Kullander, SO	12024	Sylhet	River at Bisnakandi, coming from Meghalaya, tributary to Surma River, riffles	Meghna	25.1753	91.88661	25 Mar 2016
168	Psilorhynchidae	*Psilorhynchus balitora*	MK572509	NRM	66044	Kullander, SO	11841	Sylhet	Piyain River at Jaflong	Meghna	25.1844	92.01642	24 Mar 2016
169	Cyprinidae	*Salmostoma sardinella*	MK572571	NRM	66998	Kullander, SO	10033	Dhaka	Padma River near Srinagar	Padma			2 Dec 2014
169	Cyprinidae	*Salmostoma sardinella*	MK572570	NRM	67038	Kullander, SO	10173	Chittagong	Halda River at Madinagut Point	Karnafuli	22.4291	91.88801	30 Nov 2014
169	Cyprinidae	*Salmostoma sardinella*	MK572572	NRM	66616	Kullander, SO	9812	Dhaka	Fish market in Shonbari, Sreenagar	Padma	23.544	90.29635	2 Dec 2014
170	Cyprinidae	*Salmostoma phulo*	MK572565	NRM	66539	Kullander, SO	10201	Chittagong	Rangamati, town fish market, fish principally from Kaptai Lake	Karnafuli	22.6497	92.18372	27 Nov 2014
170	Cyprinidae	*Salmostoma phulo*	MK572567	NRM	66689	Kullander, SO	9932	Dhaka	Turag River at Kamarpare, near Dhaka city	Padma	23.8985	90.38438	1 Dec 2014
170	Cyprinidae	*Salmostoma phulo*	MK572568	NRM	66543	Kullander, SO	10190	Chittagong	Rangamati, town fish market, fish principally from Kaptai Lake	Karnafuli	22.6497	92.18372	27 Nov 2014
170	Cyprinidae	*Salmostoma phulo*	MK572566	NRM	67155	Kullander, SO	10502	Chittagong	Sangu River at Dhopachari	Sangu	22.2061	92.13399	13 May 2015
170	Cyprinidae	*Salmostoma phulo*	MK572569	NRM	69294	Kullander, SO	11489	Chittagong	Ananda Bazar Market in Brahmanbaria; fishes from Titas and Meghna River	Meghna			20 Mar 2016
170	Cyprinidae	*Salmostoma phulo*	MK572564	DU	6059	Kullander, SO	6059	Rajshahi	Batpara point, Jamuna River, Feni	Jamuna	24.1395	89.60121	2014-07-17
171	Cyprinidae	*Salmostoma* sp KUSHIYARA	MK572554	NRM	65767	Kullander, SO	12097	Sylhet	Kushiyara River left bank in Fenchuganj at junction with Juri River	Meghna	24.7054	91.95432	22 Mar 2016
171	Cyprinidae	*Salmostoma* sp KUSHIYARA	MK572557	NRM	65763	Kullander, SO	12093	Sylhet	Kushiyara River left bank in Fenchuganj at junction with Juri River	Meghna	24.7054	91.95432	22 Mar 2016
171	Cyprinidae	*Salmostoma* sp KUSHIYARA	MK572553	NRM	65765	Kullander, SO	12095	Sylhet	Kushiyara River left bank in Fenchuganj at junction with Juri River	Meghna	24.7054	91.95432	22 Mar 2016
171	Cyprinidae	*Salmostoma* sp KUSHIYARA	MK572558	NRM	65766	Kullander, SO	12096	Sylhet	Kushiyara River left bank in Fenchuganj at junction with Juri River	Meghna	24.7054	91.95432	22 Mar 2016
171	Cyprinidae	*Salmostoma* sp KUSHIYARA	MK572556	NRM	65768	Kullander, SO	12098	Sylhet	Kushiyara River left bank in Fenchuganj at junction with Juri River	Meghna	24.7054	91.95432	22 Mar 2016
171	Cyprinidae	*Salmostoma* sp KUSHIYARA	MK572555	NRM	65764	Kullander, SO	12094	Sylhet	Kushiyara River left bank in Fenchuganj at junction with Juri River	Meghna	24.7054	91.95432	22 Mar 2016
172	Cyprinidae	*Securicula gora*	MK572591	DU	6065	Kullader, SO	6065	Rajshahi	Batpara point, Jamuna River, Feni	Jamuna	24.1395	89.60121	2014-07-17
173	Cyprinidae	*Salmostoma bacaila*	MK572561	NRM	69282	Kullander, SO	11477	Sylhet	Kushiyara River left bank in Fenchuganj at junction with Juri River	Meghna	24.7054	91.95432	22 Mar 2016
173	Cyprinidae	*Salmostoma bacaila*	MK572563	NRM	66997	Kullander, SO	10032	Dhaka	Padma River near Srinagar				2 Dec 2014
173	Cyprinidae	*Salmostoma bacaila*	MK572559	NRM	66736	Kullander, SO	10113	Rangpur	Kortoa River, at Bogorbari point, under Teprigonj union, about 5 km N of Devigonj town	Padma	26.1375	88.71884	3 May 2014
173	Cyprinidae	*Salmostoma bacaila*	MK572560	NRM	67279	Kullander, SO	11110	Chittagong	Rangamati, fish landing pier, fish from Kaptai Lake	Karnafuli	22.6501	92.18571	27-28 Nov 2014
173	Cyprinidae	*Salmostoma bacaila*	MK572562	DU	6252	Rahman, MM	6252	Chittagong	Mohua River, Feni		22.9118	91.50275	2015-05-30
174	Cyprinidae	*Cabdio morar*	MK572086	NRM	69236	Kullander, SO	11474	Sylhet	Fish Market in Golapgonj	Meghna			23 Mar 2016
174	Cyprinidae	*Cabdio morar*	MK572084	NRM	68393	Kullander, SO	12542	Sylhet	Fish Market in Golapgonj	Meghna			23 Mar 2016
174	Cyprinidae	*Cabdio morar*	MK572085	NRM	69281	Kullander, SO	11476	Sylhet	Kushiyara River left bank in Fenchuganj at junction with Juri River	Meghna	24.7054	91.95432	22 Mar 2016
174	Cyprinidae	*Cabdio morar*	MK572087	NRM	66729	Kullander, SO	10117	Rangpur	Tista barrage area, about 7 km E of Dimla Upazila	Tista River	26.1802	89.03997	2 May 2014
175	Cyprinidae	*Opsarius bendelisis*	MK572058	NRM	67305	Kullander, SO	10446	Chittagong	Shaila Propat Waterfall and downstream pools	Sangu	22.1515	92.2163	14 May 2015
175	Cyprinidae	*Opsarius bendelisis*	MK572062	NRM	66731	Kullander, SO	9982	Rangpur	Kortoa River, at Bogorbari point, under Teprigonj union, about 5 km N of Devigonj town	Ganga River	26.1375	88.71884	3 May 2014
175	Cyprinidae	*Opsarius bendelisis*	MK572060	NRM	69373	Kullander, SO	11652	Sylhet	Piyain River at Jaflong	Meghna	25.1844	92.01642	24 Mar 2016
175	Cyprinidae	*Opsarius bendelisis*	MK572059	NRM	67291	Kullander, SO	11161	Chittagong	Bangchora hill stream in Kaptai, near hydropower station	Karnafuli	22.4958	92.18671	28 Nov 2014
175	Cyprinidae	*Opsarius bendelisis*	MK572061	NRM	66507	Kullander, SO	9994	Rangpur	Tista barrage area, about 7 km E of Dimla Upazila	Tista	26.1802	89.03997	2 May 2014
175	Cyprinidae	*Opsarius bendelisis*	MK572398	NRM	67227	Kullander, SO	10615	Chittagong	Small stream in Dopachari Wildlife Sanctuary	Sangu	22.2277	92.1255	13 May 2015
176	Cyprinidae	*Barilius barila*	MK572053	NRM	69372	Kullander, SO	11645	Sylhet	Kushiyara River left bank in Fenchuganj at junction with Juri River	Meghna	24.7054	91.95432	22 Mar 2016
176	Cyprinidae	*Barilius barila*	MK572056	NRM	66983	Kullander, SO	10081	Dhaka	Padma River near Srinagar	Padma			2 Dec 2014

176	Cyprinidae	*Barilius barila*	MK572055	NRM	66981	Kullander, SO	10055	Dhaka	Padma River near Srinagar	Padma			2 Dec 2014
176	Cyprinidae	*Barilius barila*	MK572057	NRM	66042	Kullander, SO	11835	Sylhet	Kushiyara River left bank in Fenchuganj at junction with Juri River	Meghna	24.7054	91.95432	22 Mar 2016
177	Cyprinidae	*Barilius barila*	MK572054	NRM	67161	Kullander, SO	10493	Chittagong	Sangu River at Dhopachari	Sangu	22.2061	92.13399	13 May 2015
178	Cyprinidae	*Opsarius barna*	MK572394	NRM	67246	Kullander, SO	10569	Chittagong	Dopachari Stream, Dudpukuria, Dopachari Wildlife Sanctuary	Sangu	22.2277	92.1256	13 May 2015
178	Cyprinidae	*Opsarius barna*	MK572395	NRM	67225	Kullander, SO	10617	Chittagong	Small stream in Dopachari Wildlife Sanctuary	Sangu	22.2277	92.1255	13 May 2015
178	Cyprinidae	*Opsarius barna*	MK572391	NRM	68240	Kullander, SO	11298	Chittagong	Kokhiyon Jhiri stream, 11 km W of Ruma	Sangu	22.058	92.34182	14 May 2015
178	Cyprinidae	*Opsarius barna*	MK572397	NRM	67166	Kullander, SO	10481	Chittagong	Sangu River at Dhopachari	Sangu	22.2061	92.13399	13 May 2015
178	Cyprinidae	*Opsarius barna*	MK572390	NRM	67800	Kullander, SO	10927	Chittagong	Maheshkhali Island, small stream in Khalmadia Chora, 8 km north of Maheshkhali	Bara Matamuhu	21.5873	91.96645	11 May 2015
178	Cyprinidae	*Opsarius barna*	MK572393	NRM	66037	Kullander, SO	11833	Sylhet	Piyain River at Jaflong	Meghna	25.1844	92.01642	24 Mar 2016
178	Cyprinidae	*Opsarius barna*	MK572392	NRM	68230	Kullander, SO	11315	Chittagong	Kokhiyon Jhiri stream, 11 km W of Ruma	Sangu	22.058	92.34182	14 May 2015
178	Cyprinidae	*Opsarius barna*	MK572396	DU	6249	Kullander, SO	6249	Chittagong	Khakra Stream	Karnafuli	23.1309	91.99845	2015-04-24
179	Cyprinidae	*Opsarius tileo*	MK572399	DU	6170a	Kullander, SO	6170	Chittagong	Feni River in Ramgarh	Feni	23.0017	91.73424	2015-04-26
179	Cyprinidae	*Opsarius tileo*	MK572402	DU	6191	Rahman, MM	6191	Chittagong	Bangmara Stream	Karnafuli	23.0644	91.90538	2015-04-24
179	Cyprinidae	*Opsarius tileo*	MK572400	DU	6170	Kullander, SO	6170	Chittagong	Feni River in Ramgarh	Feni	23.0017	91.73424	2015-04-26
179	Cyprinidae	*Opsarius tileo*	MK572401	DU	6260	Rahman, MM	6260		Bangladesh, no data				
180	Cyprinidae	Raiamas bola	MK572539	DU	6280	Rahman, MM	6280		Atrai River	Atrai			
181	Cobitidae	*Lepidocephalichthys* cf *caudofurcat*	MK572290	NRM	66649	Kullander, SO	9962	Chittagong	Kamila stream, tributary to Karnafuli River, 22 km south of Rangamati	Karnafuli	22.5281	92.19861	28 Nov 2014
181	Cobitidae	*Lepidocephalichthys* sp	MK572288	NRM	69264	Kullander, SO	11585	Dhaka	Meghna River at Ashuganj Point	Meghna	24.0463	91.00562	20 Mar 2016
182	Cobitidae	*Lepidocephalichthys goalparensis*	MK572305	NRM	66188	Kullander, SO	12117	Sylhet	River at Bisnakandi, coming from Meghalaya, tributary to Meghna River	Meghna	25.1592	91.89299	25 Mar 2016
182	Cobitidae	*Lepidocephalichthys goalparensis*	MK572307	NRM	66187	Kullander, SO	12113	Sylhet	River at Bisnakandi, coming from Meghalaya, tributary to Meghna River	Meghna	25.1592	91.89299	25 Mar 2016
182	Cobitidae	*Lepadocephalichthys goalparensis*	MK572306	NRM	69330	Kullander, SO	11581	Sylhet	Kushiyara River left bank in Fenchuganj at junction with Juri River	Meghna	24.7054	91.95432	22 Mar 2016
182	Cobitidae	*Lepidocephalichthys goalparensis*	MK572304	NRM	69328	Kullander, SO	11599	Dhaka	Bhairab Fish Market, fish from Meghna River	Meghna	24.05	90.98333	20 Mar 2016
183	Cobitidae	*Lepidocephalichthys caudofurcatus*	MK572302	NRM	69329	Kullander, SO	11571	Sylhet	Hail Haor, Baikka Beel, at tourist center	Meghna	24.3619	91.70741	21 Mar 2016
183	Cobitidae	*Lepidocephalichthys caudofurcatus*	MK572301	NRM	69347	Kullander, SO	11638	Sylhet	Small canal near Jaintiapur on Sylhet-Jaflong road N2	Meghna	25.1519	92.11258	24 Mar 2016
183	Cobitidae	*Lepidocephalichthys caudofurcatus*	MK572303	NRM	69261	Kullander, SO	11604	Sylhet	Small roadside pool near Jaflongon Sylhet-Jaflong road N2	Meghna	25.178	92.04595	24 Mar 2016
184	Cobitidae	*Lepidocephalichthys irroratus*	MK572312	NRM	69766	Kullander, SO	12318	Sylhet	Kushiyara River drainage, Chatul floodplain, E of Golapganj	Meghna	24.843	92.05717	23 Mar 2016
184	Cobitidae	*Lepidocephalichthys irroratus*	MK572313	NRM	69741	Kullander, SO	12293	Chittagong	Titas River in Akhaura	Meghna	23.8843	91.20121	19 Mar 2016
185	Cobitidae	*Lepidocephalichthys* sp1	MK572297	NRM	67424	Kullander, SO	10751	Chittagong	Barochora stream, 4 km south of Cox's Bazar		21.3959	92.00372	8 May 2015
186	Cobitidae	*Lepidocephalichthys* sp3	MK572291	NRM	66737	Kullander, SO	10114	Rangpur	Tista barrage area, about 7 km E of Dimla Upazila	Tista River	26.1802	89.03997	2 May 2014
186	Cobitidae	*Lepidocephalicthys* sp3	MK572294	DU	6217	Kullander, SO	6217	Mymenshing	Sherpur market	Jamuna	25.0229	90.01728	2015-03-21
187	Cobitidae	*Lepidocephalichthys annandalei*	MK572299	NRM	66720	Kullander, SO	10118	Dhaka	Someshwari River in Durgapur	Jamuna	25.1092	90.67818	4 Apr 2014
187	Cobitidae	*Lepidocephalichthys annandalei*	MK572300	NRM	65632	Kullander, SO	12031	Sylhet	River at Bisnakandi, coming from Meghalaya, tributary to Surma River, riffles	Meghna	25.1753	91.88661	25 Mar 2016
188	Cobitidae	*Lepidocephalichthys annandalei*	MK572289	NRM	66503	Kullander, SO	10134	Rangpur	Atrai River at Dhaspara village, 8 km N of Khanshama	Padma	26.0003	88.71325	3 May 2014
189	Cobitidae	*Lepidocephalichthys guntea*	MK572311	NRM	66707	Kullander, SO	10128	Sylhet	Madopkundo water falls, hill streams about 5 km from Dakshinbagh railway station	Meghna	24.5547	92.224	26 Apr 2014
189	Cobitidae	*Lepidocephalichthys guntea*	MK572308	NRM	66721	Kullander, SO	10120	Dhaka	Someshwari River in Durgapur	Jamuna	25.1092	90.67818	4 Apr 2014
189	Cobitidae	*Lepidocephalichthys guntea*	MK572310	DU	6202	Kullander, SO	6202	Mymenshing	River	Jamuna			2015-03-21
190	Cobitidae	*Lepidocephalichthys* sp2	MK572296	NRM	67240	Kullander, SO	10472	Chittagong	Kuikhali, small pool close to trail from Kudong Cave		21.0938	92.16811	9 May 2015
190	Cobitidae	*Lepidocephalichthys* sp2	MK572293	NRM	66782	Kullander, SO	10233	Chittagong	Stream at southeastern margin of Chittagong University campus	Karnafuli	22.4603	91.7967	29 Nov 2014
190	Cobitidae	*Lepidocephalichthys* sp2	MK572295	NRM	67325	Kullander, SO	10464	Chittagong	Udalbania, stream in Dudpukuria-Dhopachori Wildlife Sanctuary	Karnafuli	22.3102	92.15844	12 May 2015
190	Cobitidae	*Lepidocephalichthys* sp2	MK572298	NRM	67979	Kullander, SO	11189	Chittagong	Udalbania, small stream left bank tributary to Karnafuli River, tobacco plantation	Karnafuli	22.3245	92.139	12 May 2015
190	Cobitidae	*Lepidocephalichthys* sp2	MK572292	DU	6169	Kullander, SO	6169	Chittagong	Taichala Stream	Karnafuli	23.1038	91.96689	2015-04-26
191	Cobitidae	*Neoeucirrhichthys maydelli*	MK572364	NRM	66727	Kullander, SO	10115	Rangpur	Atrai River at Dhaspara village, 8 km N of Khanshama	Padma	26.0003	88.71325	3 May 2014
192	Cobitidae	*Neoeucirrhichthys maydelli*	MK572362	NRM	66194	Kullander, SO	11944	Sylhet	River at Bisnakandi, coming from Meghalaya, tributary to Meghna River	Meghna	25.1592	91.89299	25 Mar 2016
192	Cobitidae	*Neoeucirrhichthys maydelli*	MK572363	NRM	69279	Kullander, SO	11633	Sylhet	Piyain River at Jaflong near Jaflong Bazar	Meghna	25.1543	92.02039	24 Mar 2016
193	Cobitidae	*Pangio pangia*	MK572428	NRM	68014	Kullander, SO	12059	Sylhet	Piyain River at Jaflong	Meghna	25.1844	92.01642	24 Mar 2016
193	Cobitidae	*Pangio pangia*	MK572425	NRM	66842	Kullander, SO	10072	Chittagong	Bangchora hill stream in Kaptai, near hydropower station	Karnafuli	22.4958	92.18671	28 Nov 2014
193	Cobitidae	*Pangio pangia*	MK572426	DU	6223	Kullander, SO	6223	Mymenshing	Old Bramaputra, BAU	Jamuna	24.7315	90.43496	2015-03-20
193	Cobitidae	*Pangio pangia*	MK572427	DU	6256	Kullander, SO	6256	Chittagong	Remacri khal	Sangu			2014-06-10
194	Cobitidae	*Cantophrys gongota*	MK572089	NRM	66516	Kullander, SO	10102	Sylhet	Pyiang River at Jaflong, about 60 km from Sylhet city	Meghna	25.1862	92.01667	25 Apr 2014
194	Cobitidae	*Cantophrys gongota*	MK572088	DU	6142	Rahman, MM	6142	Mymenshing	Itna Haor	Jamuna	24.5309	90.2721	2014-10-02
195	Balitoridae	*Balitora brucei*	MK572048	NRM	69039	Kullander, SO	11362	Sylhet	River at Bisnakandi, coming from Meghalaya, tributary to Surma River, riffles	Meghna	25.1753	91.88661	25 Mar 2016
195	Balitoridae	*Balitora brucei*	MK572047	NRM	69402	Kullander, SO	11678	Chittagong	Lal bridge, hillstream	Sangu	22.1863	92.21177	14 Feb 2016
195	Balitoridae	*Balitora brucei*	MK572050	DU	6226	Kullander, SO	6226	Mymenshing	Chellakhali Stream	Jamuna	25.2543	90.03265	2015-03-21
196	Botiidae	*Botia rostrata*	MK572074	NRM	66514	Kullander, SO	9986	Sylhet	Pyiang River at Jaflong, about 60 km from Sylhet city	Meghna	25.1862	92.01667	25 Apr 2014
196	Botiidae	*Botia rostrata*	MK572073	NRM	66594	Kullander, SO	9829	Dhaka	Fish market in Shonbari, Sreenagar	Padma	23.544	90.29635	2 Dec 2014
197	Botiidae	*Botia lohachata*	MK572072	NRM	66508	Kullander, SO	10103	Rangpur	Kortoa River, at Bogorbari point, under Teprigonj union, about 5 km N of Devigonj town	Padma	26.1375	88.71884	3 May 2014
197	Botiidae	*Botia lohachata*	MK572071	NRM	66593	Kullander, SO	9830	Dhaka	Fish market in Shonbari, Sreenagar	Padma	23.544	90.29635	2 Dec 2014
198	Botiidae	*Botia dario*	MK572070	NRM	67522	Kullander, SO	11141	Dhaka	Fish market in Shonbari, Sreenagar	Padma	23.544	90.29635	2 Dec 2014
198	Botiidae	*Botia dario*	MK572069	NRM	66515	Kullander, SO	9993	Sylhet	Pyiang River at Jaflong, about 60 km from Sylhet city	Meghna	25.1862	92.01667	25 Apr 2014
198	Botiidae	*Botia dario*	MK572068	DU	6051	Rahman, MM	6051	Rajshahi	Baghmara point , Shjadpur, Shirajgonj	Padma	24.1395	89.60121	2014-07-17
199	Nemacheilidae	*Schistura scaturigina*	MK572587	NRM	66738	Kullander, SO	9981	Rangpur	Kortoa River, at Bogorbari point, under Teprigonj union, about 5 km N of Devigonj town	Padma	26.1375	88.71884	3 May 2014
199	Nemacheilidae	*Schistura scaturigina*	MK572589	NRM	65787	Kullander, SO	12071	Sylhet	Surma River left bank, at Kheaghat point, 1,5 km upstream from Golapganj	Meghna	24.8612	91.99371	23 Mar 2016
199	Nemacheilidae	*Schistura scaturigina*	MK572588	NRM	66986	Kullander, SO	10304	Dhaka	Padma River near Srinagar	Padma			2 Dec 2014
199	Nemacheilidae	*Schistura scaturigina*	MK572586	DU	6174	Kullander, SO	6174	Chittagong	Bangmara Stream	Karnafuli	23.0644	91.90538	2015-04-24
199	Nemacheilidae	*Schistura scaturigina*	MK572590	DU	6236	Kullander, SO	6236	Chittagong	Taichala Stream	Karnafuli	23.1038	91.96689	2015-04-26
200	Nemacheilidae	*Schistura corica*	MK572361	NRM	66742	Kullander, SO	10110	Sylhet	Pyiang River at Jaflong, about 60 km from Sylhet city	Meghna	25.1862	92.01667	25 Apr 2014
200	Nemacheilidae	*Schistura corica*	MK572359	NRM	66219	Kullander, SO	12050	Sylhet	Piyain River at Jaflong	Meghna	25.1844	92.01642	24 Mar 2016
200	Nemacheilidae	*Schistura corica*	MK572360	NRM	69260	Kullander, SO	11597	Sylhet	Piyain River at Jaflong near Jaflong Bazar	Meghna	25.1543	92.02039	24 Mar 2016
200	Nemacheilidae	*Schistura corica*	MK572358	DU	6248	Kullander, SO	6248	Mymenshing	Chilakhali stream	Jamuna	25.2543	90.03265	2015-03-21
200	Nemacheilidae	*Schistura corica*	MK572357	DU	6209	Kullander, SO	6209	Mymenshing	Chilakhali stream	Jamuna	25.2543	90.03265	2015-03-21
201	Nemacheilidae	*Schistura* aff *corica*	MK572574	NRM	68227	Kullander, SO	11318	Chittagong	Kokhiyon Jhiri stream, 11 km W of Ruma	Sangu	22.058	92.34182	14 May 2015

202	Nemacheilidae	*Schistura* aff *sijuensis*	MK572583	NRM	65759	Kullander, SO	12119	Chittagong	Lal bridge, hillstream	Sangu	22.1863	92.21177	14 Feb 2016
202	Nemacheilidae	*Schistura* aff *sijuensis*	MK572581	NRM	67673	Kullander, SO	10715	Chittagong	Small stream tributary to Sangu River,under bridge near Munlai Para village, E of Ruma	Sangu	22.0328	92.41753	14 May 2015
202	Nemacheilidae	*Schistura* aff *sijuensis*	MK572585	NRM	67674	Kullander, SO	10716	Chittagong	Small stream tributary to Sangu River,under bridge near Munlai Para village, E of Ruma	Sangu	22.0328	92.41753	14 May 2015
202	Nemacheilidae	*Schistura sijuensis*	MK572582	NRM	69318	Kullander, SO	11656	Sylhet	River at Bisnakandi, coming from Meghalaya, tributary to Surma River, riffles	Meghna	25.1753	91.88661	25 Mar 2016
202	Nemacheilidae	*Schistura sijuensis*	MK572584	DU	6200	Kullander, SO	6200	Mymenshing	Chilakhali stream	Jamuna	25.2543	90.03265	2015-03-21
203	Nemacheilidae	*Schistura savona*	MK572577	NRM	66739	Kullander, SO	9988	Sylhet	Pyiang River at Jaflong, about 60 km from Sylhet city	Meghna	25.1862	92.01667	25 Apr 2014
203	Nemacheilidae	*Schistura savona*	MK572580	NRM	66814	Kullander, SO	10299	Chittagong	Hillstream at northwestern margin of Chittagong University campus, in and below rapids	Karnafuli	22.4738	91.78312	29 Nov 2014
203	Nemacheilidae	*Schistura savona*	MK572578	NRM	66728	Kullander, SO	10116	Dhaka	Someshwari River in Durgapur	Jumna River	25.1092	90.67818	4 Apr 2014
203	Nemacheilidae	*Schistura savona*	MK572573	NRM	66815	Kullander, SO	10297	Chittagong	Hillstream at northwestern margin of Chittagong University campus, in and below rapids	Karnafuli	22.4738	91.78312	29 Nov 2014
203	Nemacheilidae	*Schistura savona*	MK572579	DU	6199	Kullander, SO	6199	Mymenshing	Chilakhali stream	Jamuna	25.2543	90.03265	2015-03-21
204	Nemacheilidae	*Schistura* sp1	MK572576	DU	6210	Kullander, SO	6210	Mymenshing	Chilakhali stream	Jamuna	25.2543	90.03265	2015-03-21
204	Nemacheilidae	*Schistura* sp1	MK572575	DU	6210	Kullander, SO	6210	Mymenshing	Chilakhali stream	Jamuna	25.2543	90.03265	2015-03-21
205	Nemacheilidae	*Paracanthocobitis abutwebi*	MK572432	NRM	66822	Kullander, SO	10183	Chittagong	Hillstream at northwestern margin of Chittagong University campus, in and below rapids	Karnafuli	22.4738	91.78312	29 Nov 2014
205	Nemacheilidae	*Paracanthocobitis abutwebi*	MK572435	NRM	67929	Kullander, SO	10821	Chittagong	Udalbania, stream at entrance to Dudpukuria-Dhopachori Wildlife Sanctuary	Karnafuli	22.3105	92.15218	12 May 2015
205	Nemacheilidae	*Paracanthocobitis abutwebi*	MK572429	NRM	67004	Kullander, SO	10369	Chittagong	Ichemoti Stream in Rangunia, below bridge	Karnafuli	22.5394	91.89877	29 Nov 2014
205	Nemacheilidae	*Paracanthocobitis abutwebi*	MK572436	NRM	67672	Kullander, SO	10719	Chittagong	Small stream tributary to Sangu River,under bridge near Munlai Para village, E of Ruma	Sangu	22.0328	92.41753	14 May 2015
205	Nemacheilidae	*Paracanthocobitis abutwebi*	MK572434	NRM	68228	Kullander, SO	11316	Chittagong	Kokhiyon Jhiri stream, 11 km W of Ruma	Sangu	22.058	92.34182	14 May 2015
205	Nemacheilidae	*Paracanthocobitis abutwebi*	MK572433	NRM	69267	Kullander, SO	11507	Sylhet	Piyain River at Jaflong near Jaflong Bazar	Meghna	25.1543	92.02039	24 Mar 2016
206	Nemacheilidae	*Paracanthocobitis mackenziei*	MK572439	NRM	67078	Kullander, SO	10346	Dhaka	Turag River at Kamarpare, near Dhaka city	Padma	23.8985	90.38438	1 Dec 2014
206	Cobitidae	*Paracanthocobitis mackenziei*	MK572438	DU	6221	Kullander, SO	6221	Mymenshing	Old Bramaputra, BAU	Jamuna	24.7315	90.43496	2015-03-20
207	Clupeidae	*Gonialosa manmina*	MK572245	NRM	67609	Kullander, SO	11079	Chittagong	Rangamati, town fish market, fish principally from Kaptai Lake	Karnafuli	22.6497	92.18372	27 Nov 2014
207	Clupeidae	*Gonialosa manmina*	MK572244	NRM	65783	Kullander, SO	12067	Dhaka	Meghna River at Ashuganj Point	Meghna	24.0463	91.00562	20 Mar 2016
207	Clupeidae	*Gonialosa manmina*	MK572246	NRM	69498	Kullander, SO	11898	Dhaka	Meghna River at Ashuganj Point	Meghna	24.0463	91.00562	20 Mar 2016
207	Clupeidae	*Gonialosa manmina*	MK572249	NRM	66541	Kullander, SO	10200	Chittagong	Rangamati, town fish market, fish principally from Kaptai Lake	Karnafuli	22.6497	92.18372	27 Nov 2014
207	Clupeidae	*Gonialosa manmina*	MK572243	NRM	67609	Kullander, SO	11078	Chittagong	Rangamati, town fish market, fish principally from Kaptai Lake	Karnafuli	22.6497	92.18372	27 Nov 2014
207	Clupeidae	*Gonialosa manmina*	MK572248	NRM	67003	Kullander, SO	10029	Dhaka	Padma River near Srinagar	Padma			2 Dec 2014
207	Clupeidae	*Gonialosa manmina*	MK572247	NRM	65782	Kullander, SO	12066	Dhaka	Meghna River at Ashuganj Point	Meghna	24.0463	91.00562	20 Mar 2016
208	Clupeidae	*Gudusia chapra*	MK572252	NRM	67090	Kullander, SO	10246	Dhaka	Turag River at Kamarpare, near Dhaka city	Padma	23.8985	90.38438	1 Dec 2014
208	Clupeidae	*Gudusia chapra*	MK572251	NRM	66542	Kullander, SO	10195	Chittagong	Rangamati, town fish market, fish principally from Kaptai Lake	Karnafuli	22.6497	92.18372	27 Nov 2014
208	Clupeidae	*Gudusia chapra*	MK572250	NRM	67605	Kullander, SO	11131	Chittagong	Rangamati, fish landing pier, fish from Kaptai Lake	Karnafuli	22.6501	92.18571	27-28 Nov 2014
209	Clupeidae	*Tenualosa ilisha*	MK572613	NRM	67603	Kullander, SO	11162	Chittagong	Halda River at Madinagut Point	Karnafuli	22.4291	91.88801	30 Nov 2014
209	Clupeidae	*Tenualosa ilisha*	MK572614	NRM	67607	Kullander, SO	11157	Dhaka	Padma River near Srinagar	Padma			2 Dec 2014
209	Clupeidae	*Tenualosa ilisha*	MK572612	NRM	67603	Kullander, SO	11174	Chittagong	Halda River at Madinagut Point	Karnafuli	22.4291	91.88801	30 Nov 2014
209	Clupeidae	*Tenualosa ilisha*	MK572610	DU	6026	Rahman, MM	6026	Barisal	Barishal Fish Landing	Meghna	22.7396	90.43307	2014-11-01
209	Clupeidae	*Tenualosa ilisha*	MK572611	DU	6025	Rahman, MM	6025	Barisal	Barishal Fish Landing	Meghna	22.7396	90.43307	2014-11-01
210	Clupeidae	*Hilsa kelee*	MK572263	DU	6092	Rahman, MM	6092	Dhaka	Hatirpul Bazar		23.7507	90.39568	2015-04-06
211	Clupeidae	*Corica soborna*	MK572139	NRM	65762	Kullander, SO	12092	Sylhet	Kushiyara River left bank in Fenchuganj at junction with Juri River	Meghna	24.7054	91.95432	22 Mar 2016
211	Clupeidae	*Corica soborna*	MK572137	NRM	66929	Kullander, SO	10290	Chittagong	Rangamati, fish landing pier, fish from Kaptai Lake	Karnafuli	22.6501	92.18571	27-28 Nov 2014
211	Clupeidae	*Corica soborna*	MK572138	NRM	66989	Kullander, SO	10086	Dhaka	Padma River near Srinagar	Padma			2 Dec 2014
211	Clupeidae	*Corica soborna*	MK572140	NRM	67040	Kullander, SO	10211	Chittagong	Halda River at Madinagut Point	Karnafuli	22.4291	91.88801	30 Nov 2014
212	Engraulididae	*Coilia dussumieri*	MK572136	NRM	66861	Kullander, SO	10194	Chittagong	Halda River at Madinagut Point	Karnafuli	22.4291	91.88801	30 Nov 2014
213	Engraulididae	*Setipinna phasa*	MK572592	NRM	67613	Kullander, SO	10950	Chittagong	Halda River at Madinagut Point	Karnafuli	22.4291	91.88801	30 Nov 2014
214	Pristigasteridae	*Ilisha melastoma*	MK572272	DU	6255	Rahman, MM	6255		No data				
215	Sillaginidae	*Sillaginopsis domina*	MK572593	NRM	66882	Kullander, SO	10163	Chittagong	Halda River at Madinagut Point	Karnafuli	22.4291	91.88801	30 Nov 2014
215	Sillaginidae	*Sillaginopsis domina*	MK572594	NRM	67595	Kullander, SO	11168	Chittagong	Halda River at Madinagut Point	Karnafuli	22.4291	91.88801	30 Nov 2014
216	Megalopidae	*Megalops cyprinoides*	MK572326	NRM	67962	Kullander, SO	11273	Chittagong	Donumia Stream, 10 km north of Teknaf town, 70 km south of Cox's Bazar	Naf	20.9233	92.26298	9 May 2015
217	Anguillidae	*Anguilla bengalensis*	MK572029	NRM	66849	Kullander, SO	10062	Chittagong	Bangchora hill stream in Kaptai, near hydropower station	Karnafuli	22.4958	92.18671	28 Nov 2014
217	Anguillidae	*Anguilla bengalensis*	MK572027	NRM	67331	Kullander, SO	10547	Chittagong	Sangu River at Dhopachari	Sangu	22.2061	92.13399	13 May 2015
217	Anguillidae	*Anguilla bengalensis*	MK572030	NRM	66565	Kullander, SO	9794	Chittagong	Woagga Stream, 4 km W of Kaptai National Park	Karnafuli	22.5091	92.14182	28 Nov 2014
217	Anguillidae	*Anguilla bengalensis*	MK572031	NRM	68254	Kullander, SO	11327	Chittagong	Small stream about 2 km north of Teknaf on Cox's Bazar-Teknaf Highway	Naf	20.8821	92.29758	10 May 2015
217	Anguillidae	*Anguilla bengalensis*	MK572028	NRM	66850	Kullander, SO	10067	Chittagong	Bangchora hill stream in Kaptai, near hydropower station	Karnafuli	22.4958	92.18671	28 Nov 2014
218	Ophichthidae	*Pisodonophis boro*	MK572488	NRM	67616	Kullander, SO	11062	Dhaka	Local fish market in Dhaka				1 Dec 2014
218	Ophichthidae	*Pisodonophis boro*	MK572492	NRM	66857	Kullander, SO	10074	Chittagong	Bangchora hill stream in Kaptai, near hydropower station	Karnafuli	22.4958	92.18671	28 Nov 2014
218	Ophichthidae	*Pisodonophis boro*	MK572491	NRM	66856	Kullander, SO	10075	Chittagong	Bangchora hill stream in Kaptai, near hydropower station	Karnafuli	22.4958	92.18671	28 Nov 2014
218	Ophichthidae	*Pisodonophis boro*	MK572489	NRM	67691	Kullander, SO	10757	Chittagong	Small stream tributary to Sangu River,under bridge near Munlai Para village, E of Ruma	Sangu	22.0328	92.41753	14 May 2015
218	Ophichthidae	*Pisodonophis boro*	MK572490	NRM	66873	Kullander, SO	10160	Chittagong	Halda River at Madinagut Point	Karnafuli	22.4291	91.88801	30 Nov 2014
219	Notopteridae	*Notopterus notopterus*	MK572369	NRM	67614	Kullander, SO	11138	Chittagong	Rangamati, fish landing pier, fish from Kaptai Lake	Karnafuli	22.6501	92.18571	27-28 Nov 2014
219	Notopteridae	*Notopterus notopterus*	MK572368	NRM	67612	Kullander, SO	11148	Dhaka	Fish market in Shonbari, Sreenagar	Padma	23.544	90.29635	2 Dec 2014
220	Notopteridae	*Chitala chitala*	MK572123	DU	6066	Rahman, MM	6066	Dhaka	Hatirpul Bazar		23.7507	90.39568	2015-04-06
221	Synbranchidae	*Monopterus cuchia*	MK572332	NRM	68315	Kullander, SO	12186	Sylhet	Kushiyara River drainage, Chatul floodplain, E of Golapganj	Meghna	24.843	92.05717	23 Mar 2016
221	Synbranchidae	*Monopterus cuchia*	MK572331	NRM	68891	Kullander, SO	12181	Chittagong	Shorail, roadside ditch 8 km north of Brahmanbaria	Meghna	24.0471	91.10258	19 Mar 2016
222	Adrianichthyidae	*Oryzias* sp1	MK572413	NRM	66563	Kullander, SO	9774	Chittagong	Woagga Stream, 4 km W of Kaptai National Park	Karnafuli	22.5091	92.14182	28 Nov 2014
222	Adrianichthyidae	*Oryzias* sp1	MK572408	NRM	66895	Kullander, SO	10147	Chittagong	Halda River at Madinagut Point	Karnafuli	22.4291	91.88801	30 Nov 2014
223	Adrianichthyidae	*Oryzias* sp4	MK572410	NRM	67882	Kullander, SO	10886	Chittagong	Donumia Stream, 10 km north of Teknaf town, 70 km south of Cox's Bazar	Naf	20.9233	92.26298	9 May 2015
224	Adrianichthyidae	*Oryzias* sp3	MK572411	NRM	67187	Kullander, SO	10526	Chittagong	Lower part of small stream tributary to Naf River, about 3 km north of Teknaf	Naf	20.8986	92.27865	10 May 2015
224	Adrianichthyidae	*Oryzias* sp3	MK572412	NRM	67185	Kullander, SO	10527	Chittagong	Lower part of small stream tributary to Naf River, about 3 km north of Teknaf	Naf	20.8986	92.27865	10 May 2015
225	Adrianichthyidae	*Oryzias* sp2	MK572409	NRM	67779	Kullander, SO	10981	Chittagong	Udalbania, small stream left bank tributary to Karnafuli River, tobacco plantation	Karnafuli	22.3245	92.139	12 May 2015
225	Adrianichthyidae	*Oryzias* sp2	MK572407	NRM	67927	Kullander, SO	10865	Chittagong	Udalbania, stream at entrance to Dudpukuria-Dhopachori Wildlife Sanctuary	Karnafuli	22.3105	92.15218	12 May 2015
226	Adrianichthyidae	*Oryzias dancena*	MK572414	NRM	69098	Kullander, SO	11453	Dhaka	Turag River	Meghna			5 Mar 2016
226	Adrianichthyidae	*Oryzias dancena*	MK572415	NRM	66177	Kullander, SO	11960	Sylhet	Fenchuganj, roadside ditch in Garuli floodplain, 4 km south of Sylhet	Meghna	24.6945	91.94553	22 Mar 2016
226	Adrianichthyidae	*Oryzias dancena*	MK572416	NRM	69089	Kullander, SO	11444	Dhaka	Turag River	Meghna			5 Mar 2016

227	Gerreidae	*Gerres setifer*	MK572216	NRM	67149	Kullander, SO	10652	Chittagong	Maheshkhali Island, Khaler Uttarkul, Barachora Stream	Bara Matamuhu	21.5141	91.96584	11 May 2015
227	Gerreidae	*Gerres setifer*	MK572218	NRM	67148	Kullander, SO	10656	Chittagong	Maheshkhali Island, Khaler Uttarkul, Barachora Stream	Bara Matamuhu	21.5141	91.96584	11 May 2015
227	Gerreidae	*Gerres setifer*	MK572217	DU	9015	Kullander, SO	9015		Dakchara chara	Ichamati			2014-11-01
228	Zenarchopterida	*Dermogenys burmanica*	MK572167	NRM	66908	Kullander, SO	10008	Chittagong	Halda River at Madinagut Point	Karnafuli	22.4291	91.88801	30 Nov 2014
228	Zenarchopterida	*Dermogenys burmanica*	MK572171	NRM	67688	Kullander, SO	10675	Chittagong	Small stream tributary to Sangu River,under bridge near Munlai Para village, E of Ruma	Sangu	22.0328	92.41753	14 May 2015
228	Zenarchopterida	*Dermogenys burmanica*	MK572165	NRM	66868	Kullander, SO	10018	Chittagong	Hillstream at northwestern margin of Chittagong University campus, in and below rapids	Karnafuli	22.4738	91.78312	29 Nov 2014
228	Zenarchopterida	*Dermogenys burmanica*	MK572170	NRM	67833	Kullander, SO	10833	Chittagong	Kudung Cave in Teknaf Game Reserve		21.0923	92.16972	9 May 2015
228	Zenarchopterida	*Dermogenys burmanica*	MK572169	NRM	67200	Kullander, SO	11322	Chittagong	Lower part of small stream tributary to Naf River, about 3 km north of Teknaf	Naf	20.8986	92.27865	10 May 2015
228	Zenarchopterida	*Dermogenys burmanica*	MK572163	NRM	67201	Kullander, SO	10450	Chittagong	Lower part of small stream tributary to Naf River, about 3 km north of Teknaf	Naf	20.8986	92.27865	10 May 2015
228	Zenarchopterida	*Dermogenys burmanica*	MK572164	NRM	66844	Kullander, SO	10069	Chittagong	Bangchora hill stream in Kaptai, near hydropower station	Karnafuli	22.4958	92.18671	28 Nov 2014
228	Zenarchopterida	*Dermogenys burmanica*	MK572166	NRM	67200	Kullander, SO	10447	Chittagong	Lower part of small stream tributary to Naf River, about 3 km north of Teknaf	Naf	20.8986	92.27865	10 May 2015
229	Belonidae	*Xenentodon cancila*	MK572631	NRM	69003	Kullander, SO	11396	Sylhet	Hail Haor, Baikka Beel, at tourist center	Meghna	24.3619	91.70741	21 Mar 2016
229	Belonidae	*Xenentodon cancila*	MK572630	NRM	67582	Kullander, SO	11106	Chittagong	Rangamati, fish landing pier, fish from Kaptai Lake	Karnafuli	22.6501	92.18571	27-28 Nov 2014
229	Belonidae	*Xenentodon cancila*	MK572629	NRM	67582	Kullander, SO	11105	Chittagong	Rangamati, fish landing pier, fish from Kaptai Lake	Karnafuli	22.6501	92.18571	27-28 Nov 2014
230	Hemiramphidae	*Hyporhamphus limbatus*	MK572268	NRM	66547	Kullander, SO	10206	Chittagong	Rangamati, town fish market, fish principally from Kaptai Lake	Karnafuli	22.6497	92.18372	27 Nov 2014
230	Hemiramphidae	*Hyporhamphus limbatus*	MK572269	NRM	66927	Kullander, SO	11321	Chittagong	Rangamati, fish landing pier, fish from Kaptai Lake	Karnafuli	22.6501	92.18571	27-28 Nov 2014
230	Hemiramphidae	*Hyporhamphus limbatus*	MK572266	NRM	69309	Kullander, SO	11640	Dhaka	Meghna River at Ashuganj Point	Meghna	24.0463	91.00562	20 Mar 2016
230	Hemiramphidae	*Hyporhamphus limbatus*	MK572267	NRM	66927	Kullander, SO	10259	Chittagong	Rangamati, fish landing pier, fish from Kaptai Lake	Karnafuli	22.6501	92.18571	27-28 Nov 2014
231	Mugilidae	*Liza melanoptera*	MK572314	NRM	66558	Kullander, SO	9805	Chittagong	Fishery Ghat, fish landing in Chittagong				30 Nov 2014
232	Mugilidae	*Planiliza parsia*	MK572493	NRM	67632	Kullander, SO	11073	Chittagong	Rangamati, town fish market, fish principally from Kaptai Lake	Karnafuli	22.6497	92.18372	27 Nov 2014
232	Mugilidae	*Planiliza parsia*	MK572494	NRM	67184	Kullander, SO	10484	Chittagong	Lower part of small stream tributary to Naf River, about 3 km north of Teknaf on Cox's Baza	Naf	20.8986	92.27865	10 May 2015
233	Mugilidae	*Minimugil cascasia*	MK572330	NRM	65786	Kullander, SO	12070	Sylhet	Surma River left bank, at Kheaghat point, 1,5 km upstream from Golapganj	Meghna	24.8612	91.99371	23 Mar 2016
233	Mugilidae	*Minimugil cascasia*	MK572329	NRM	67001	Kullander, SO	10031	Dhaka	Padma River near Srinagar	Padma			2 Dec 2014
234	Mugilidae	*Rhinomugil corsula*	MK572548	NRM	66880	Kullander, SO	10162	Chittagong	Halda River at Madinagut Point	Karnafuli	22.4291	91.88801	30 Nov 2014
234	Cyprinidae	*Puntius chola*	MK572529	NRM	65780	Kullander, SO	12110	Sylhet	Kushiyara River left bank in Fenchuganj at junction with Juri River	Meghna	24.7054	91.95432	22 Mar 2016
234	Mugilidae	*Rhinomugil corsula*	MK572549	NRM	67617	Kullander, SO	11158	Chittagong	Halda River at Madinagut Point	Karnafuli	22.4291	91.88801	30 Nov 2014
234	Mugilidae	*Rhinomugil corsula*	MK572547	NRM	66999	Kullander, SO	10034	Dhaka	Padma River near Srinagar	Padma			2 Dec 2014
234	Mugilidae	*Rhinomugil corsula*	MK572550	NRM	67174	Kullander, SO	10554	Chittagong	Sangu River at Dhopachari	Sangu	22.2061	92.13399	13 May 2015
235	Teraponidae	*Terapon jarbua*	MK572615	NRM	67591	Kullander, SO	11036	Chittagong	Fishery Ghat, fish landing in Chittagong				30 Nov 2014
235	Terapontidae	*Terapon jarbua*	MK572616	DU	9014	Rahman, SO	9014		Noakhali Khal	Noakhali			2014-11-01
236	Ambassidae	*Parambassis lala*	MK572446	NRM	66614	Kullander, SO	9862	Dhaka	Fish market in Shonbari, Sreenagar	Padma	23.544	90.29635	2 Dec 2014
236	Ambassidae	*Parambassis lala*	MK572445	NRM	66648	Kullander, SO	9998	Chittagong	Kamila stream, tributary to Karnafuli River, 22 km south of Rangamati	Karnafuli	22.5281	92.19861	28 Nov 2014
236	Ambassidae	*Parambassis lala*	MK572444	NRM	68251	Kullander, SO	11281	Dhaka	Market at Ashulia Point, Turag River	Padma			1 Dec 2014
236	Ambassidae	*Parambassis lala*	MK572443	NRM	68817	Kullander, SO	11406	Sylhet	Hakaluki Haor in Borolek	Meghna	24.6617	92.03478	26 Mar 2016
236	Ambassidae	*Parambassis lala*	MK572447	DU	6208	Rahman, SO	6208	Mymenshing	Sherpur market	Jamuna	25.0229	90.01728	2015-03-21
236	Ambassidae	*Parambassis lala*	MK572442	DU	6227	Rahman, SO	6227	Mymenshing	Sherpur market	Jamuna	25.0229	90.01728	2015-03-21
237	Ambassidae	*Chanda nama*	MK572097	NRM	67031	Kullander, SO	10172	Chittagong	Halda River at Madinagut Point	Karnafuli	22.4291	91.88801	30 Nov 2014
237	Ambassidae	*Chanda nama*	MK572099	NRM	66186	Kullander, SO	12136	Sylhet	River at Bisnakandi, coming from Meghalaya, tributary to Meghna River, sandy bed stream	Meghna	25.1592	91.89299	25 Mar 2016
237	Ambassidae	*Chanda nama*	MK572096	NRM	66974	Kullander, SO	10022	Dhaka	Padma River near Srinagar	Padma			2 Dec 2014
237	Ambassidae	*Chanda nama*	MK572095	NRM	66671	Kullander, SO	9914	Dhaka	Turag River at Kamarpare, near Dhaka city	Padma	23.8985	90.38438	1 Dec 2014
237	Ambassidae	*Chanda nama*	MK572098	NRM	67796	Kullander, SO	10967	Chittagong	Maheshkhali Island, small stream in Khalmadia Chora, 8 km north of Maheshkhali Upazila	Bara Matamuhu	21.5873	91.96645	11 May 2015
237	Ambassidae	*Chanda nama*	MK572100	DU	6135	Rahman, SO	6135	Mymenshing	Itna Haor	Jamuna	24.5309	91.10439	2014-10-02
238	Ambassidae	*Parambassis ranga*	MK572451	NRM	69069	Kullander, SO	11466	Sylhet	Hail Haor, Baikka Beel, at tourist center	Meghna	24.3619	91.70741	21 Mar 2016
238	Ambassidae	*Parambassis ranga*	MK572452	NRM	69070	Kullander, SO	11417	Sylhet	Hail Haor, Baikka Beel, at tourist center	Meghna	24.3619	91.70741	21 Mar 2016
238	Ambassidae	*Parambassis ranga*	MK572455	NRM	68818	Kullander, SO	11408	Sylhet	Hakaluki Haor in Borolek	Meghna	24.6617	92.03478	26 Mar 2016
238	Ambassidae	*Parambassis ranga*	MK572448	NRM	66926	Kullander, SO	10258	Chittagong	Rangamati, fish landing pier, fish from Kaptai Lake	Karnafuli	22.6501	92.18571	27-28 Nov 2014
238	Ambassidae	*Parambassis ranga*	MK572456	NRM	68225	Kullander, SO	11343	Chittagong	Kokhiyon Jhiri stream, 11 km W of Ruma	Sangu	22.058	92.34182	14 May 2015
238	Ambassidae	*Parambassis ranga*	MK572450	NRM	69074	Kullander, SO	11472	Sylhet	Kushiyara River left bank in Fenchuganj at junction with Juri River	Meghna	24.7054	91.95432	22 Mar 2016
238	Ambassidae	*Parambassis ranga*	MK572457	NRM	66910	Kullander, SO	10438	Chittagong	Rangamati, fish landing pier, fish from Kaptai Lake	Karnafuli	22.6501	92.18571	27-28 Nov 2014
238	Ambassidae	*Parambassis ranga*	MK572453	NRM	69077	Kullander, SO	11414	Sylhet	Piyain River at Jaflong	Meghna	25.1844	92.01642	24 Mar 2016
238	Ambassidae	*Parambassis ranga*	MK572458	NRM	66911	Kullander, SO	10433	Chittagong	Rangamati, fish landing pier, fish from Kaptai Lake	Karnafuli	22.6501	92.18571	27-28 Nov 2014
238	Ambassidae	*Parambassis ranga*	MK572454	NRM	69076	Kullander, SO	11411	Sylhet	Piyain River at Jaflong	Meghna	25.1844	92.01642	24 Mar 2016
238	Ambassidae	*Parambassis ranga*	MK572449	DU	6228	Rahman, SO	6228	Mymenshing	Sherpur market	Jamuna	25.0229	90.01728	2015-03-21
239	Ambassidae	*Parambassis baculis*	MK572440	NRM	69073	Kullander, SO	11465	Sylhet	Kushiyara River left bank in Fenchuganj at junction with Juri River	Meghna	24.7054	91.95432	22 Mar 2016
240	Ambassidae	*Parambassis bistigmata*	MK572441	NRM	69056	Kullander, SO	11421	Sylhet	Piyain River at Jaflong	Meghna	25.1844	92.01642	24 Mar 2016
241	Cichlidae	*Oreochromis niloticus*	MK572406	NRM	66765	Kullander, SO	10425	Chittagong	Roadside ditch in Sultanpur	Karnafuli	22.5422	91.8997	29 Nov 2014
241	Cichlidae	*Oreochromis niloticus*	MK572405	NRM	66766	Kullander, SO	10386	Chittagong	Roadside ditch in Sultanpur	Karnafuli	22.5422	91.8997	29 Nov 2014
242	Anabantidae	*Anabas cobojius*	MK572023	NRM	67569	Kullander, SO	11085	Chittagong	Rangamati, town fish market, fish principally from Kaptai Lake	Karnafuli	22.6497	92.18372	27 Nov 2014
242	Anabantidae	*Anabas cobojius*	MK572024	NRM	67592	Kullander, SO	11027	Chittagong	Fishery Ghat, fish landing in Chittagong				30 Nov 2014
242	Anabantidae	*Anabas cobojius*	MK572025	DU	9011	Rahman, SO	9011	Chittagong	East Sonarpara				2014-10-30
243	Anabantidae	*Anabas testudineus*	MK572026	DU	6102	Rahman, MM	6102	Dhaka	Hatirpul Bazar		23.7507	90.39568	2015-04-06
